# Photoinduced Synthesis of Methylated Marine Cyclopeptide Galaxamide Analogs with Isoindolinone as Anticancer Agents

**DOI:** 10.3390/md20060379

**Published:** 2022-06-05

**Authors:** Shimei Xiao, Zhiqiang Wang, Huanli Zhang, Lei Zhao, Qingran Chang, Xiong Zhang, Rui Yan, Xiaodan Wu, Yingxue Jin

**Affiliations:** Key Laboratory of Photochemical Biomaterials and Energy Storage Materials, College of Chemistry & Chemical Engineering, Harbin Normal University, Harbin 150025, China; xxssmm987@163.com (S.X.); hxzhl0923@163.com (H.Z.); a18646354097@163.com (L.Z.); 18324507787@163.com (Q.C.); xiongzhang1204@163.com (X.Z.); yanrui-1981@163.com (R.Y.); smile_200325@163.com (X.W.)

**Keywords:** cyclic peptide, photoinduced cyclization, galaxamide, antitumor, stereochemistry

## Abstract

The methylation of amino acid residues has played an important role in the biological function of bioactive peptides. In this paper, various methyl-modified and stereostructural-modified marine cyclopeptide galaxamide analogs with isoindolinone were synthesized by a photoinduced single electron transfer cyclization reaction. It was found that the single-methyl substitution was beneficial for the bioactivity of cyclic analogs with isoindolinone fragments, and the influence of methylation on bioactivity is uncertain and is sometimes case-specific. The compound with a single methyl group at Gly^5^ (compound **8**) showed the strongest antiproliferative activity against HepG-2 cells. The tumor cell apoptosis, cell cycle, mitochondrial membrane potential, intracellular Ca^2+^ concentration and lactate dehydrogenase activity have been studied extensively to evaluate the antitumor potential of compound **8**. Western blotting tests showed that compound **8** could decrease the MDM2 level and increase p53 levels efficiently. Careful molecular docking suggested that cyclic peptide 8 could bind firmly with MDM2 oncoprotein, indicating that MDM2 may be a potential drug target of the prepared peptides.

## 1. Introduction

The treatment and prevention of cancer have been a major problem plaguing the medical community, and exploring new anticancer drugs has been the goal of mankind’s relentless pursuit. Cyclic peptides, which exist widely in nature (especially marine organisms), have been found with antitumor, anti-bacterial, anti-inflammatory and anti-viral activities [1]. Thus far, more than 100 cyclic peptide drugs have been developed and utilized in the clinic [2]. Cyclic peptides display stronger metabolic stability than linear peptides, allowing them to have longer effects in vivo because of their constrained ring structure, which prevents cleaving by peptidase [3]. The restricted rotation of the C-N amide bond and constrained cyclic conformation also give the cyclopeptides strong rigidity, thus facilitating their binding to the target protein. Moreover, because of their medium molecular weights, small cyclic peptides have been recognized to be able to combine the advantages of small molecules (easy absorption) and polypeptides (high specificity), and with the great efforts of plant chemists and marine biologists, more and more biologically active natural cyclic peptides have been found, providing important resources for drug development [3,4].

Galaxamide is a cyclic pentapeptide isolated from the seaweed *Galaxaura filamentosa* in Xisha Islands, South China Sea, in 2008, consisting of five leucine residues with two N-methyl groups. Despite its simple chemistry structure, galaxamide showed strong in vitro antitumor activities against renal carcinoma GRC-1 cell lines and human hepatocellular carcinoma HepG-2 cell lines [5], and many analogs of galaxamide have been observed with promising antitumor activity [6,7,8,9]. For example, Xiao et al. have modified galaxamide by replacing L-leucine with D-phenylalanine and L-phenylalanine and found that the introduction of D-phenylalanine enhances its antitumor activity [5], probably due to the fact that phenylalanine changes the conformation and enhances conformational constraint, making it more strongly bound to the target protein.

**Figure 1:**
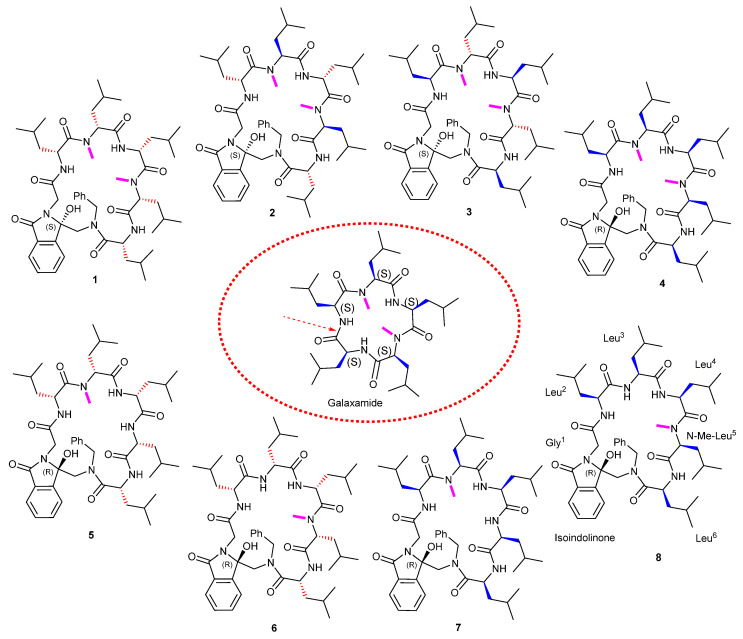
Galaxamide and the designed analogs (compounds **1**–**4**: each cyclic peptide contains two N-methyl groups with interlaced D/L amino acid residues; compounds **5**–**8**: each one contains a N-methyl group with all-D or all-L-amino acid residues). The number of amino acids is shown in compound **8** as a representative.

Meanwhile, N-methylation has been reported to be able to influence the bioactivity of peptides significantly due to the steric hinder and the blocking of the formation of hydrogen bonds between the polypeptides and proteolytic enzyme cleavage sites [10,11]. Our group has modified galaxamide recently by inserting an unnatural isoindolinone fragment groups and removing the methyl groups [12]. We found that after removing the two N-methyl groups of galaxamide and introducing a rigid isoindolinone fragment into the skeleton by intramolecular photoinduced single electron transfer cyclization reaction (IPETC), the obtained cyclic peptide analogs still maintained significant antitumor activity against HepG-2 cells, and the peptide containing the D-leucine fraction showed stronger bioactivity than that with L-leucine residues, suggesting that the isoindolinone fragment together with the D-residues may play an important role in treatment. A similar phenomenon has been observed in our research on analogs of the natural cyclic peptide phakellistatin 2 [13]. Inspired by the previous findings, we tried to further modify galaxamide to explore novel antitumor cyclopeptides here. Specifically, the isoindolone fragment was embedded into the cyclic skeleton by the IPETC method, and the L-amino acid residues were changed into interlaced D/L residues based on the finding that the interlaced D/L amino acid residues were beneficial for the bioactivity of some cyclic peptides [14], thus designing compounds **1**–**4**. Considering the importance of the methyl group, as mentioned earlier, we further designed analogs **5**–**8** by changing the number and position of the N-methyl groups (N-methyl-Leu^3^ or N-methyl-Leu^5^), as shown in Figure 1. The cyclopeptides (**1**–**8**) have been synthesized by our IPETC reaction. Their in vitro bioactivity against human hepatocellular carcinoma HepG-2 cells was studied by MTT assay. The influence of representative compound **8** on cell apoptosis, cell cycle and the expression level of oncoprotein MDM2, together with the molecular docking, were studied in detail. Due to the important influence of stereo-configuration on biological activity, we also elucidated the configurations by experimental and theoretical circular dichroism analysis.

## 2. Results and Discussion

### 2.1. Synthesis

**Scheme 1:**
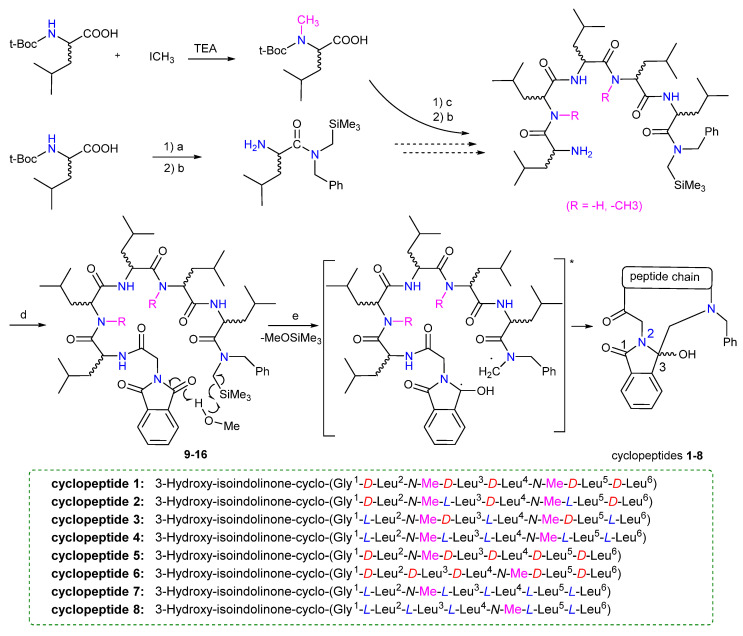
Liquid-phase synthesis of linear peptides **9**–**16** and cyclopeptides **1**–**8**. (**a**) BnTMSA, EEDQ, DCM; (**b**) TFA, DCM, rt; (**c**) (N-Boc-aa)_n_, EEDQ, DCM; (**d**) N-phthalimido-glycine acid chloride, DCM, rt; (**e**) >290 nm UV-light radiation, MeOH. The concentrations of **9**–**16** were 5 × 10^−3^ mol/L.

Galaxamide is defined as cyclo-(*L*-Leu-*L*-*N*-Me-Leu-*L*-Leu-*L*-Leu-*L*-*N*-Me-Leu-*L*-Leu). The structural modification of galaxamide is initiated by splitting the amide bond in the peptide sequence, followed by the introduction of phthalimide and photoinduced cyclization of linear peptides, as reported in our previous research [12,13]. In the present study, the photoinduced substrate linear peptide is prepared by a liquid-phase method, as shown in Scheme 1.

By using *t*-BOC-leucine and (*N*-trimethylsilylmethyl) benzylamine as the starting material, the carboxy-amidated *N*-Boc amino acids were prepared in the presence of the condensing agent *N*-ethoxycarbonyl-2-ethoxy-1,2-dihydroquinoline (EEDQ), and the linear peptides were expanded by liquid-phase synthesis using trifluoroacetic acid (TFA) as a deprotecting agent, during which the *N*-Me-leucine was introduced according to the design requirements. *N*-Me-leucine used here was prepared by adopting the *t*-BOC- leucine and iodomethane as raw materials in the presence of TEA. Then, the nitrogen-terminal amino groups were masked with phthaloylglycyl chloride to obtain *N*-phthalimido-terminally trimethylsilyl peptides **9**–**16** under dilute conditions. The intermediates were synthesized in high yields, and the crude reaction products were used directly in the reactions without further purification (using thin-layer chromatography to check the reactions). The dose ratio of linear peptides, *N*-Boc-amino acids and EEDQ in the amidation reaction was maintained at 1:1.1:1.1 to ensure complete reactions. Then, the photoreaction was performed in dilute methanol solution (10^−3^ M), where the photoreaction precursor linear peptide **9**–**16** was irradiated with a Pyrex glass filter (λ > 290 nm) for 30 min under nitrogen protection with a medium-pressure mercury lamp (Scheme 1). The solution was cooled by circulating water in a cylindrical glass jacket around the photoreactor. Methanol was used as a hydrogen donor, and the polar solvation effect was helpful for the stability of the leaving group SiMe_3_^+^.

During the photoinduced process, the chain-like molecule (linear peptide) was excited and formed intramolecular zwitterionic biradicals after the departure of MeOSiMe_3_, and then the donator- and acceptor-free radicals at each end connected with each other to produce the cyclic compound [12,13]. After coupling, the C_3_ carbonyl peak of phthalimido at δ 180 ppm disappeared in the ^13^C NMR spectrum, followed by a new sp^3^ hybridized C_3_ peak at δ 85 ± 5 ppm, which is the characteristic NMR peak position of C_3_ after the IPETC reaction. The chemical shifts of C-3 in ^13^C NMR of all peptides **1**–**8** were around δ 85 ± 5 ppm in our paper, which suggested the successful preparation of the cyclopeptides. The resulting mixture was purified by column chromatography to give the galaxamide analogs 3-hydroxy-isoindolinone-cyclopeptides (**1**–**8**). The specific preparation process and characterization data can be found in the Supporting Materials.

### 2.2. Determination of Absolute Configurations (ACs)

The carbonyl carbon atom in phthalimide fragment is latently chiral, and once a photoinduced cyclization is completed, carbonyl carbon atom would convert into a chiral carbon. We initially tried to determine the C-3 configuration by X-ray crystal diffraction analysis, but failed to obtain the single crystals. Herein we investigated the stereostructures by experimental electronic circular dichroism (ECD) combined with the quantum chemical calculations. The combination of experimental and theoretical ECD has been widely used to elucidate the absolute configuration of chiral structures [15,16].

**Figure 2:**
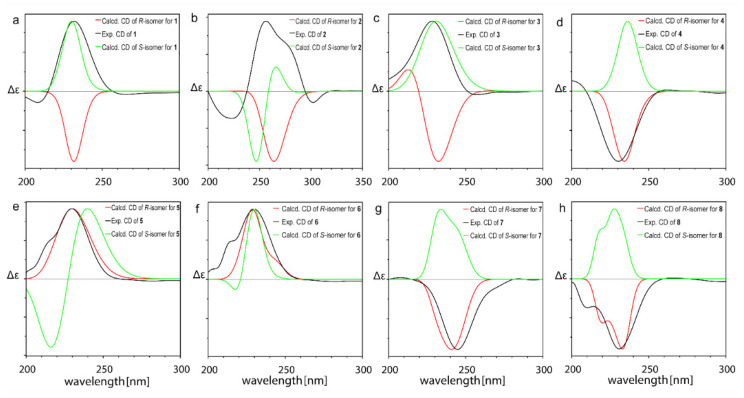
Experimental and calculated (TDDFT/ωB97XD/cc-pVDZ) ECD of the prepared compounds **1** (**a**), **2** (**b**), **3** (**c**), **4** (**d**), **5** (**e**), **6** (**f**), **7** (**g**), and **8** (**h**).

In this paper, conformational search was firstly carried out to define stable conformations prior to ECD calculations. Due to the conformational restriction of the N-methyl group and isoindolone fragment, only a few stable low energy conformers for each configuration were found (as shown in Table S1). The obtained molecules were directly optimized with B3LYP/6–31G (d, p) and the CD spectra were simulated at the ωB97XD/cc-pVDZ level based on the time-dependent density functional theory method (TDDFT) [17,18]. The AC of C-3 position can be distinguished when the simulated spectrum of a selected configuration is consistent with the experimental one. The simulated and experimental ECD spectra of the 8 cyclic compounds in methanol (2.0 × 10^−5^ M) in the 200–350 nm range are presented in Figure 2. The stable structures were presented in Figure S1.

The calculated ECD spectrum of the S conformer for cyclopeptide **1** matches well with the experimental cotton effects (CEs), while the simulated ECD of the *R*-configuration shows an obvious positive CE at 232 nm, suggesting an *S*-configuration for **1** (Figure 2a). The theoretical ECD of compound **2** shows some deviations from the experimental spectrum, yet the experimental negative CE at 225 nm, the strong positive CE at 255 nm and the weak negative CE at 302 nm were reproduced by the theoretical simulation of S-isomer at a negative CE at 248 nm, a positive CE at 267 nm and a weak negative CE at 290 nm, while the simulated *R*-isomer showed a remarkable strong negative CE at 263 nm, opposite to the experimental one and indicating an *S*-configuration (Figure 2b). The sign of the experimental CEs of **3** at 230 nm is well reproduced by the calculated ECD spectra of the *S* conformer at 232 nm, also indicating an *S*-configuration (Figure 2c). The experimental ECD of cyclopeptide **4** (black line) in Figure 2d shows an obvious negative CE at 231 nm, and the calculated ECD spectra of the R-conformer (red line) are consistent with the sign of the experimental CE at 234 nm, which clearly supports the assignment of a C-3-*R* configuration to cyclic peptide **4**. The experimental ECD of compound **5** in Figure 2e shows a broad positive CE at 229 nm with a shoulder at 212 nm, and the calculated ECD spectrum of the *R*-conformer also presents a broad CE at 230 nm without a negative CE, while the theoretical ECD of the *S*-conformer presents an obvious negative CE at 217 nm, opposite to the positive experimental shoulder at 212 nm and indicating an *R*-configuration for cyclopeptide **5**. Similarly, the experimental ECD of cyclopeptide **6** presents a broad positive CE at 230 nm with a shoulder at 213 nm, and the calculated spectrum of the *R*-conformer shows a broad positive CE at 229 nm (Figure 2f), while the simulated ECD for the *S*-conformer displays a negative CE at 218 nm, which also indicates an *R*-configuration.

The sign of the experimental CEs of **7** at 245 nm is well reproduced by calculated ECD spectra of the *R* conformer at 241 nm, also suggesting an *R-*configuration (Figure 2g). The experimental ECD of **8** shows a remarkable negative CE at 231 nm with a shoulder at 210 nm, both of which are well-reproduced by the simulation of an *R-*configuration at 233 nm and 220 nm, yet the calculated S-configuration shows opposite positive CEs, presenting an *R*-configuration (Figure 2h).

### 2.3. MTT Method to Detect the Effect of Drugs on Cell Proliferation

The antitumor activity of the prepared cyclic peptides against human hepatocellular carcinoma cells (HepG-2), human cervical cancer cell lines (HeLa) and human breast cancer cells (MDA-MB-231) was examined by MMT assay, and the mouse fibroblast L929 cells were used as the model cells to evaluate the toxicity of cyclopeptides to normal cell lines. Due to the absence of an authentic standard, taxol was chosen as a control because it is a widely used chemotherapeutic drug for the treatment of various cancers, including liver cancer.

The IC_50_ values of the synthesized compounds against the three cell lines are shown in Table 1. Most of them showed moderate antiproliferative activity, except for cyclopeptide **8**, which presented the strongest bioactivity among the prepared cyclopeptides with an IC_50_ of 11.1 μM. The IC_50_ value of paclitaxel against HepG-2 cells was 40.9 μM in our parallel experiment. It can be found that most of our compounds showed superior or parallel activity at the cellular level compared to the positive control, indicating the potential for clinical application. Compound **8** has the most significant IC_50_ of 11 μM against HepG-2 cells, implying that this compound could be considered a valuable lead for future structural modification. It could also be found that compounds **5**, **6**, **7** and **8** with double methyl groups showed enhanced antiproliferative activity against HepG-2 cells compared to compounds **1**, **2**, **3** and **4** with single methyl groups, indicating that the incorporation of methyl amino acids may be unfavorable for biological activity in our case. In addition, the chiral change of amino acids in compounds **1–4** did not significantly change the antiproliferative activity, suggesting that the spatial adjustment of such hydrophobic compounds may not greatly influence the binding to the target. We speculated that the chiral change may not greatly affect the hydrophobic interface for the bi-methyl substituted cyclopeptides. Indeed, as we display below, the prepared peptides containing isoindolinone may target an MDM oncoprotein to block the p53-MDM2 interactions and realize its antitumor activities. Because p53-MDM2 interactions involve broad hydrophobic protein-protein interactions, the slight change in the hydrophobic interface of small cyclic peptides may not influence the binding between cyclopeptides and MDM2 protein.

Moreover, a moderate antiproliferative ability was observed for all compounds against MDA-MB-231 cells. However, the weaker cytotoxicity to L929 cells revealed that they have a degree of selectivity towards cancer cells but not normal cells, which is to say, the prepared cyclic compounds were regarded as potent and broad-spectrum antitumor agents. While the compounds discussed in this paper have relatively modest activity, we believe that this research has disclosed some clues for peptide-based drug design; that is, that methylation is not always effective in enhancing the bioactivity of small cyclic peptides and also that broader molecular surface area leads to a stronger binding force, which might give useful clues for the study of protein-protein interactions.

### 2.4. Apoptosis Detection by Flow Cytometry Analysis

According to the preliminary MTT assay, compound **8** was observed to have promising antiproliferative activity; hence, we tried to further study the mechanism underlying cell death. It is recognized that inducing apoptosis is one of the most important ways to lead to cell death by chemotherapy [19]. Here, we performed a phosphatidylserine cytosine analysis to check the induction of apoptosis by compound **8** on HepG-2 cells.

HepG-2 cells were treated with 0, 5.5, 11 and 22 µM of compound **8** and incubated for 48 h, then double-stained carefully by membrane-linked protein Annexin-V-fluorescein isothiocyanate (FITC) and propidium iodide (PI) for flow cytometry analysis. Generally, in the early stages of apoptosis, phosphatidylserine flips from the inner cell membrane to the surface of the cell membrane. The exposed phosphatidylserine is recognized by an extracellular calcium-dependent phospholipid-binding protein Annexin-V; hence, FITC-labeled Annexin-V can bind specifically to phosphatidylserine and be used to detect apoptosis cells by flow cytometry [20]. Propidium iodide (PI) is a nucleic acid dye that can only cross the membrane of advanced apoptosis and necrotic cells, reddening the nucleus. Therefore, when the drug-treated cells are double-stained by the two fluorescent dyes, early and late apoptosis and necrotic cells can be clearly distinguished.

**Figure 3:**
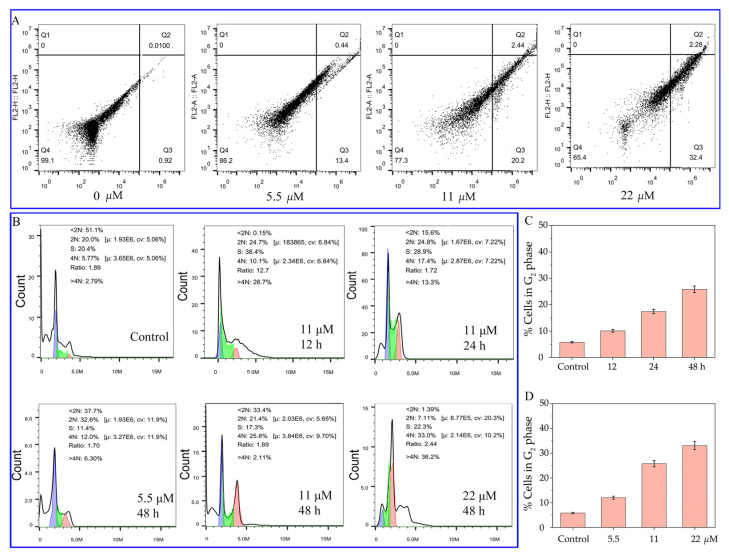
(**A**) The apoptosis detection of HepG-2 cells treated with compound **8**. (**B**) Effect of compound **8** on DNA content of HepG-2 cells checked by flow cytometry. (**C**) Bar graph of HepG-2 cells in G2 phase after drug treatment (11 µM) within different time periods. (**D**) Bar graph of HepG-2 cells in G2 phase after treatment with different concentrations of compound **8**.

As shown in Figure 3, normal cultured HepG-2 cells (0 h) had good viability (in the lower left region) (Figure 3A). With increasing drug concentration, the percentage of late apoptosis and necrotic cells (upper right quadrant) remained stable in the range of 0.44–2.28%, yet the percentage of apoptosis cells increased from 0.92% to 32.4%. We could conclude that compound **8** mainly leads to the early apoptosis of HepG-2 cells, displaying a certain concentration-dependent activity.

### 2.5. Effect of Compound ***8*** on Cell Cycle

Compound **8** exhibits a significant ability to inhibit proliferation, and one of the important ways in which antitumor drugs affect cell proliferation is through regulation of the cell cycle. The four phases in the cell cycle, mitosis (M), gap 1 (G1), synthesis of DNA (S), and gap 2 (G2), can be analysized by a commonly used dye, propidium iodide (PI), which could intercalate into the major groove of double-stranded DNA and produce a highly fluorescent adduct that can be detected due to its broad emission around 600 nm. In this research, the drug-treated tumor cells were fixed overnight at 4 °C with 70% ice-cold ethanol and then treated with Rnase A/PI for 60 min at 37 °C and analyzed by flow cytometry [21].

As shown in Figure 3B, a significant dose-dependent cell cycle arrest was observed in the G2/M phase of treated cells after 48 h of treatment with the desired concentration (0, 5.5, 11, 22 μM) of compound **8**. The G2/M phase cells increased from 12.0% to 33.0% along with the increase in concentration (Figure 3C) in a manner obviously different from the control group (5.77%). In addition, upon fixing the drug concentration to 11 μM, the G2/M phase cells increased from 5.77% to 10.1%, 17.4% and 25.8%, respectively, with increasing culture time (12, 24 and 48 h). The above analysis suggests that compound **8** could induce cycle arrest in the G2/M phase in both a time-dependent and concentration-dependent manner, subsequently leading to cell apoptosis and producing the antiproliferative effect.

### 2.6. Effect of Compound ***8*** on Mitochondrial Membrane Potential

An important event in the early apoptosis stage is the decrease in mitochondrial membrane potential (MMP), which is always ahead of the presence of other apoptosis features, such as cell shrinkage, DNA breakage and chromatin condensation [22]; hence, analysis of MMP can be used to monitor drug-induced early apoptosis. It has been found that cationic fluorescent probes can selectively accumulate in the mitochondria of living cells. A classical fluorescent dye, rhodamine 123, is selectively sequestered by active mitochondrial respiration, but is washed out in cells with decreased MMP, so that the dye fluorescence intensity reflects the level of mitochondrial activity and can be applied in the monitoring of MMP [23].

**Figure 4:**
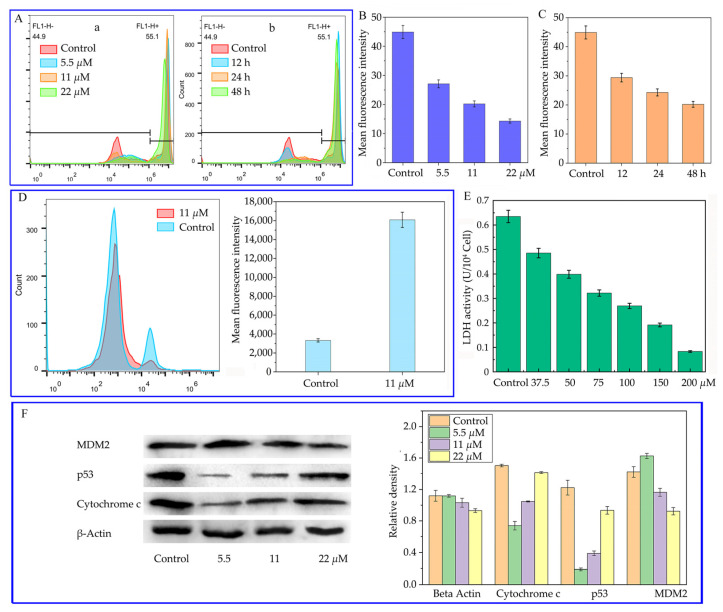
(**A**) Effect of compound **8** on the mitochondrial membrane potential of HepG-2 cells. (**B**) The histogram for mean mitochondrial fluorescent intensity of cells treated with solvent and desired concentrations (5.5, 11, 22 μM) of compound **8**. *p* < 0.01. (**C**) The histogram for the mean mitochondrial fluorescent intensity of cells after treatment of compound **8** (11 μM) for different times. *p* < 0.01. (**D**) The effect of compound **8** on the level of cytosolic Ca^2+^. (**E**) Effect of compound **8** on the activity of lactate dehydrogenase in HepG-2 cells. (**F**) Effect of peptide **8** on the levels of MDM2, p53 and cytochrome c in HepG-2 cells (the statistical results were based on the gray value).

In this study, we have studied the change of MMP of HepG-2 cells after treatment with different concentrations (0, 5.5, 11 and 22 µM within 48 h) and different time periods (12, 24 and 48 h with 11 µM) of compound **8**. Cells were stained with rhodamine 123 and analyzed by flow cytometry. Figure 4A shows the mean fluorescence intensity of HepG-2 cells after treatment with compound **8**. It can be observed that the fluorescence intensity in the drug groups decreases obviously compared to the control group (only treatment with the solvent), suggesting that the mitochondrial membrane potential of drug-treated cells was reduced. Figure 4B shows a histogram of the mean fluorescence intensity of Figure 4A(a), which directly gives the decreased fluorescence intensity from 44.9% to 27.1%, 20.2% and 14.3%. Figure 4C shows the histogram of the mean fluorescence intensity of Figure 4A(b), which also reveals the decreased fluorescence intensity from 44.9% to 29.4%, 24.3% and 20.2%. The aforementioned analysis clearly discloses that the cyclic peptides can affect the mitochondrial membrane potential and induce apoptosis, consistent with the findings in cell cycle analysis.

### 2.7. Effect of Compound ***8*** on Intracellular Ca^2+^ Levels and Lactate Dehydrogenase Activity

Intracellular calcium signaling has been found to be able to regulate various processes. It has been clear that subtle changes in Ca^2+^ concentration, such as intracellular calcium overload, can regulate and trigger apoptosis by inducing the production of mitochondrial trifunctional protein [24]. In this paper, we tried to study the influence of our prepared cyclic peptide on the intracellular calcium ion concentration. The cell-permeable fluorescent dye, Fluo-3 AM, is the most commonly used indicator to detect intracellular calcium ion concentration. Fluo-3 AM does not emit fluorescence without calcium ions, yet after entering the cells, it is hydrolyzed by esterase into Fluo-3, which binds with calcium ions in cells and emits strong fluorescence (526 nm) after 488 nm excitation [25]. Based on this principle, intracellular calcium ion concentrations can be detected by flow cytometry. In this paper, HepG-2 cells treated with solvent and the desired concentration of compound **8** for 24 h were collected and co-incubated with Fluo-3 AM. After loading, intracellular Ca^2+^ levels were measured by flow cytometry. As shown in Figure 4D, the fluorescence intensity of tumor cells increased obviously after drug treatment, which was 4.83 times higher than that of the control group, suggesting that compound **8** could increase intracellular calcium ion concentrations and mediate cell apoptosis.

In addition, lactate dehydrogenase (LDH) has been observed to be highly expressed in many tumors. The accumulation of lactate dehydrogenase can produce lactate metabolites and cause the deterioration of tumor cells [26]. By inhibiting lactate dehydrogenase activity, the energy supply of tumor cells would be blocked, thus reducing their metastasis and progression. Therefore, in this paper, we also investigated the inhibitory activity of compound **8** on lactate dehydrogenase in HepG-2 cells. It has been known that LDH could catalyze lactic acid to generate pyruvate, which could react with 2,4-dinitrophenylhydrazine (DNPH) to form dinitrophenyl-pyruvate and form a reddish-brown product after reacting with an alkaline solution. The obtained product can be assessed by visible spectrophotometry. Nowadays, an LDH activity assay kit (Beijing Solarbio Science & Technology, Beijing, China) based on the aforementioned principle can be conveniently used to determine the cellular lactate dehydrogenase activity. As shown in Figure 4E, treatment of HepG-2 cells with compound **8** resulted in a significant decrease in lactate dehydrogenase activity, disclosing a significant inhibition of lactate dehydrogenase in HepG-2 cells.

Moreover, as mentioned previously and in others’ reports [6], the apoptosis of HepG-2 induced by galaxamide might be related to a mitochondria-mediated pathway. It has been known that with the decrease in the mitochondrial membrane potential, the cytochrome C in mitochondrion is released to the cytosol and can be detected [27]. Therefore, we have also checked the content of cytochrome C in the cytosol by Western blotting to further verify the mitochondrial apoptotic pathway. As shown in Figure 4F, with the increase in peptide concentrations, the level of cytochrome C in the cytosol increased significantly compared with that of the control group, verifying that the cyclopeptides exerted an influence on this mitochondrial-dependent pathway.

### 2.8. The Influence on MDM2 and p53 Expression of Compound ***8***

The p53 tumor suppressor plays a key role in preventing tumor development [28]. In cancers retaining wild-type p53, the function of p53 can be suppressed by a variety of factors, and the murine double minute 2 (MDM2) protein is an important endogenous cellular inhibitor of p53 [29]. Therefore, inhibiting or blocking MDM2 protein should be an efficient strategy for the development of an antitumor agent. Actually, various MDM2 inhibitors have been studied and screened in recent years, including small molecules and polypeptides [30]. Many cyclic stapled peptides have been found with the promising binding ability to MDM2 protein that could efficiently inhibit p53-MDM interactions to re-activate the tumor, suppressing potential of p53 [31,32,33]. Considering that the isoindolinone fragment is always present in the structure of MDM2 inhibitors [34,35] and plays an important role in binding to the target protein, we tried to detect the MDM2 and p53 protein levels by Western blotting to assess the inhibiting ability of cyclic peptides containing isoindolinone fragments to MDM2 oncoprotein.

The expression of p53 and MDM2 proteins in HepG-2 cells were checked after treatment with compound **8** for 48 h, and beta-actin was used as the control. It was obvious that the level of beta-actin remained stable with increasing drug concentration, yet the level of MDM2 decreased gradually, leading to the increase in p53 protein expression in HepG-2 cells (Figure 4F). Our research clearly showed that the galaxamide analogs containing isoindolinone fragments could inhibit MDM2 function and re-activate the tumor suppressor function of p53, and the MDM2 oncoprotein may be regarded as a potential drug target for galaxamide analogs.

### 2.9. Molecular Docking to MDM2 Protein

The p53-MDM2 protein-protein interactions mainly involved broad van der Waals interactions between the Phe19, Trp23, and Leu26 in p53 and the hydrophobic gap in MDM2 composed of Met50, Lys51, Gly58, Ile61, Met62, His73, Val75, Val93 and His96 [31]. The earlier Western blotting experiment has found that the cyclopeptides could decrease the MDM2 level and increase the p53 level. To further investigate the binding mode between the cyclopeptide and MDM2 protein, we have here tried to perform a molecular docking of compound **8** with the hydrophobic gap in MDM2 to evaluate the binding energy, hydrogen bond number and spatial location by AutoDock Tools. Generally, a small binding energy indicates a strong interaction between drugs and targets, and a binding energy less than −5.0 kcal/mol means that the ligand molecule has a good binding ability [36].

Table 2 shows the molecular docking binding energy between the eight cyclopeptides and MDM2 protein (PDB ID: 3EQS). The structure of cyclic peptide **8** used in docking is based on the optimized structure at DTF/B3LYP/6–31G (d, *p*) level, as mentioned earlier. It can be found that cyclopeptide **8** presents the strongest interactions with MDM2 protein with a binding energy of −10.33 kcal mol^−1^ compared with that of other compounds, consistent with the experimental finding for the strongest inhibitability of cyclopeptide **8**. It was also found that the single methyl-substituted analogs (**6**, **7** and **8**) showed stronger binding affinity than the di-methyl substituted analogs **1**–**4**, also consistent with the experimental observations, implying the bi-methyl substitution may not be beneficial for binding. Moreover, compounds **4** and **8** feature the same chiral amino acids (all *S*-configurations) yet different numbers of methyl groups (two methyl groups for compound **4** and one for compound **8**). However, compound **8** presented an eight times stronger antitumor activity than **4**, similar to the binding energy.

**Figure 5:**
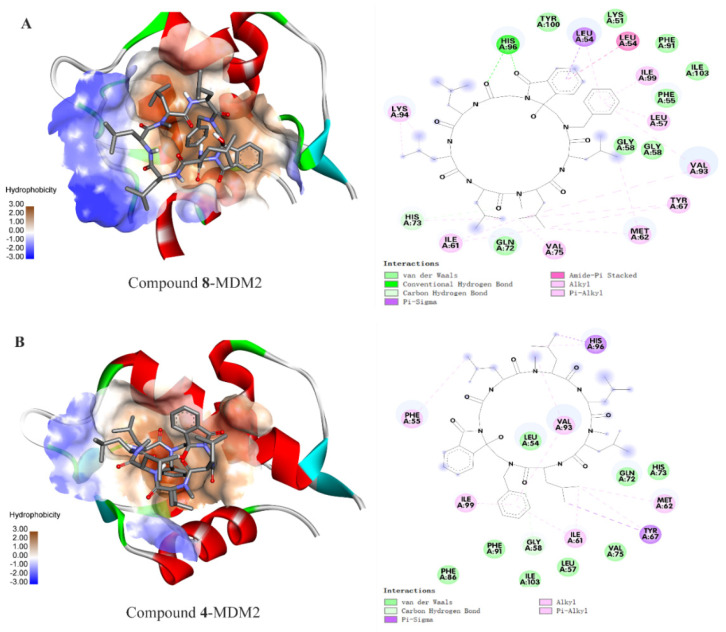
(**A**) The 2D and 3D docking mode between MDM2 and the compound **4**; (**B**) The docking mode between MDM2 and the compound **8**. The peptide **8** presents a more strengthening steric structure, and compound **4** is more crouching with a smaller molecular surface area that is not able to contact with the target widely.

Why do the two compounds present different bioactivities just because of a little difference in their methyl groups? Notwithstanding the moderate bioactivities of the prepared compounds, we believe it is meaningful to find the explanation from the docking mode between the two compounds and MDM2. Figure 5 shows the forces and the binding sites between cyclopeptides (**4** and **8**) and MDM2 (3EQS). The strong binding for compound **8** could be explained from many aspects (Figure 5A). Firstly, a conventional hydrogen bond between the carbonyl groups in compound **8** and the His96 of MDM2, and broad van der Waals interactions between the cyclic peptide and Lys51, Phe55, Gly58, Phe91, Tyr100, and Ile103 in MDM2 mainly account for the tight bindings. Moreover, the interactions between the isoindolinone group and Leu54 through amide-π and σ-π interactions further enhance the binding force. Some other hydrophobic interactions between the abundant isopropyl side chain of cyclopeptide and Val75, Ile61, Met62, Tyr67, Val93 and Lys94, and between the phenyl groups of cyclopeptide and Ile99, Leu57, and Val 93, also contribute to the binding. Combined with the observation of 3D docking in the hydrophobic cavity, we can conclude that the isoindolinone and phenyl play an important role in molecular binding. Moreover, there is no observable binding for a single methyl group in compound **8** with MDM2 protein, suggesting that the methylation may exert its function by influencing the steric structure of the peptide.

As a comparison, the docking of compound **4** with MDM2 protein was also discussed in Figure 5B. The weak binding energy for compound **4** may be attributed to the absence of the conventional hydrogen bond and the interactions between isoindolinone and the target. Only some weak hydrophobic interactions between the cyclic peptide **4** and MDM2 can be observed. Therefore, based on the above analysis, we concluded that the introduction of isoindolinone into galaxamide could facilitate the binding of cyclic peptide analogs to MDM2 protein, and the methylation is not always effective in enhancing the bioactivity of small cyclic peptides. Moreover, it can be observed visually that compound **8** presents a more strengthening steric structure and could interact with the target protein broadly, yet compound **4** is more crouching with a smaller molecular surface area that is not able to contact with the target widely. As mentioned earlier, the binding of p53 with MDM2 involves broad protein-protein interaction (PPI) through the hydrophobic forces between various amino acid residues around the contact area [31], and those peptides mimicking p53 to bind MDM2 through broad PPI have been found to have excellent antitumor activity and promising applicable prospects. Therefore, while the compounds discussed in this paper have relatively modest activity, our findings have provided powerful evidence that a larger molecular surface area would lead to a stronger binding force and might give useful clues for the study of protein-protein interactions.

## 3. Materials and Methods

### 3.1. General

Boc-L-leucine, Boc-D-leucine, di-tert-butyl ecarbonate, N-ethoxycarbonyl-2-ethoxy-1,2-dihydroquinoline (EEDQ), trifluoroacetic acid (TFA), N-[(trimethylsilyl)methyl]benzylamine, and phthalylglycyl chloride were purchased from Sinopharm Chemical Reagent Co. Dulbecco’s modified Eagle medium (DMEM), penicillin, streptomycin and fetal bovine serum (FBS) were purchased from Beijing Dingguo Biotechnology Co. (Beijing, China). Fluo-3 AM, rhodamine 123, RNase and propidium iodide (PI) were purchased from Beyotime Biotechnology (Jiangsu, China). A Lactate Dehydrogenase Kit was bought from Beijing Solarbio Science & Technology (Beijing, China). Mass spectra were recorded on a JEOL JMS-700 spectrometer. AMX400 NMR (Bruker, Bremen, Germany) was used to record ^1^H and ^13^C NMR spectra. A 450 W medium-pressure mercury lamp (HANOVIA Specialty Lighting LLC, Fairfield, NJ, USA) filtered by Pyrex glass (λ > 290 nm) was used for photoinduced excitation in a JRS-7825-34 photoreactor (Julabo, Seelbach, Germany). Hep G2 (human liver cancer cell line ATCC HB-8065), MD-MAB-231 (human breast cancer cell line ATCC HTB-26) and L929 (mouse fibroblast cell line ATCC CCL-1) cells were provided by the Harbin Institute of Technology, Harbin, China.

### 3.2. Synthesis of the Photoreaction Precursor Linear Peptides (***9***–***16***)

The synthesis of linear peptides is similar to our previous report [37].

Generally, *N*-Boc-amino acid (10 mmol) was used as the starting material, N-benzyl-1-(trimethylsilyl)-methanamine (1 equivalent, 10 mmol) was used as a masking agent for the carboxyl end group, 2-ethoxy-1-ethoxycarbonyl-1,2-dihydroquinoline (EEDQ) (1.5 molar equivalents, 15 mmol in 10 mL of THF) was used as a condensing agent, and trifluoroacetic acid (TFA) (3 molar equivalents) was used as a deprotecting agent to prepare the N-benzyl-1-(trimethylsilyl)-methanamine-substituted linear peptides (Scheme 1). Finally, the electron acceptor phthalimide glycine (1.1 molar equivalent, using dichloromethane as solvent) was introduced to obtain the photoreaction precursor N-phthalimide-peptides (**9**–**16**).

#### 3.2.1. N-Phthalimido-Gly-Leu-N-Me-Leu-Leu-N-Me-Leu-Leu-Si(CH_3_)_3_ (**9**)

White solid (yield 78%). ^1^HNMR(CDCl_3_) ***δ***: 0.01~0.20 (m, 9H, SiMe_3_), 0.90~0.95 (m, 30H, CH_3_), 1.19~1.35 (m, 5H, CH(CH_3_)_2_),1.41~1.62 (m, 10H, CH_2_CH(CH_3_)_2_), 2.93~3.04 (m, 2H, CH_2_SiMe_3_), 3.12~3.26 (m, 6H, NCH_3_), 4.43~5.51 (m, 9H, CHCH_2_CH(CH_3_)_2_ and NCH_2_CO and CH_2_Ph), 7.25~7.38 (m, 5H, ArH), 7.71~7.89 (m, 4H, Phthaloyl); ^13^CNMR(CDCl_3_) ***δ***: −1.3, 22.4, 22.8, 22.9, 23.3, 23.4, 23.5, 24.6, 24.7, 24.8, 24.9, 30.7, 37.9, 38.8, 41.6, 41.7, 42.5, 46.7, 47.6, 53.2, 53.8, 61.4, 123.4, 126.8, 127.7, 128.8, 132.2, 133.9, 136.5, 165.8, 166.2, 167.8, 167.9, 170.6, 173.1. HRMS (ESI) *m*/*z* calcd for C_53_H_83_N_7_O_8_SiNa^+^ (M + Na)^+^ 996.59646, found 996.59668.

#### 3.2.2. N-Phthalimido-Gly-Leu-N-Me-Leu-Leu-N-Me-Leu-Leu-Si(CH_3_)_3_ (**10**)

White solid (yield 72%). ^1^HNMR(CDCl_3_) ***δ***: 0.03~0.18 (m, 9H, SiMe_3_), 0.66~0.99 (m, 30H, CH_3_), 1.25~1.42 (m, 5H, CH(CH_3_)_2_),1.59~1.82 (m, 10H, CH_2_CH(CH_3_)_2_), 2.80~2.88 (m, 2H, CH_2_SiMe_3_), 2.96~3.07 (m, 6H, NCH_3_), 4.38~5.30 (m, 9H, CHCH_2_CH(CH_3_)_2_ and NCH_2_CO and CH_2_Ph), 7.12~7.38 (m, 5H, ArH), 7.74~7.88 (m, 4H, Phthaloyl); ^13^CNMR(CDCl_3_) ***δ***:−1.1, 21.6, 21.8, 22.2, 23.0, 23.2, 23.5, 24.8, 24.9, 24.9, 30.6, 35.3, 36.2, 38.6, 40.5, 40.6, 41.0, 41.5, 47.7, 48.6, 48.7, 53..1, 54.4, 54.6, 123.5, 126.9, 1275, 128.8, 132.1, 134.0, 137.0, 166.3, 167.6, 170.3, 170.7, 171.8, 171.9, 173.5, 173.5. HRMS (ESI) *m*/*z* calcd for C_53_H_83_N_7_O_8_SiNa^+^ (M + Na)^+^ 996.59646, found 996.59717.

#### 3.2.3. N-Phthalimido-Gly-Leu-N-Me-Leu-Leu-N-Me-Leu-Leu-Si(CH_3_)_3_ (**11**)

White solid (yield 75%). ^1^HNMR(CDCl_3_) ***δ***: 0.03~0.18 (m, 9H, SiMe_3_), 0.90~0.99 (m, 30H, CH_3_), 1.27~1.41 (m, 5H, CH(CH_3_)_2_),1.60~1.82 (m, 10H, CH_2_CH(CH_3_)_2_), 2.76~2.79 (m, 2H, CH_2_SiMe_3_), 2.94~3.07 (m, 6H, NCH_3_), 4.39~5.31 (m, 9H, CHCH_2_CH(CH_3_)_2_ and NCH_2_CO and CH_2_Ph), 7.27~7.38 (m, 5H, ArH), 7.74~7.87 (m, 4H, Phthaloyl); ^13^CNMR(CDCl_3_) ***δ***:−1.1, 21.6, 21.8, 22.1, 23.0, 23.2, 23.3, 23.5, 24.8, 24.8, 24.9, 30.6, 35.4, 36.2, 40.5, 41.0, 41.5, 47.7, 47.8, 48.7, 53.1, 54.4, 54.5, 67.1, 123.5, 126.9, 127.5, 128.8, 132.1, 134.0, 137.0, 167.6, 170.3, 170.7, 171.8, 171.9, 173.5. HRMS (ESI) *m*/*z* calcd for C_53_H_83_N_7_O_8_SiNa^+^ (M + Na)^+^ 996.59646, found 996.59717.

#### 3.2.4. N-Phthalimido-Gly-Leu-N-Me-Leu-Leu-N-Me-Leu-Leu-Si(CH_3_)_3_ (**12**)

White solid (yield 73%). ^1^HNMR(CDCl_3_) ***δ***: 0.04~0.17 (m, 9H, SiMe_3_), 0.88~0.95 (m, 30H, CH_3_), 1.28~1.31 (m, 5H, CH(CH_3_)_2_),1.44~1.66 (m, 10H, CH_2_CH(CH_3_)_2_), 2.89~2.93 (m, 2H, CH_2_SiMe_3_), 3.05~3.46 (m, 6H, NCH_3_), 4.20~5.52 (m, 9H, CHCH_2_CH(CH_3_)_2_ and NCH_2_CO and CH_2_Ph), 7.24~7.38 (m, 5H, ArH), 7.71~7.87 (m, 4H, Phthaloyl); ^13^CNMR(CDCl_3_) ***δ***:−1.3, 21.7, 22.1, 22.8, 23.3, 23.5, 24.5, 24.6, 24.7, 24.8, 330.7, 38.7, 41.5, 41.7, 42.5, 46.7, 47.6, 51.0, 53.2, 53.8, 61.5, 123.4, 126.8, 127.7, 128.8, 132.2, 133.9, 134.2, 136.5, 165.9, 166.2, 167.8, 170.7, 171.2, 173.0, 173.1. HRMS (ESI) *m*/*z* calcd for C_53_H_83_N_7_O_8_SiNa^+^ (M + Na)^+^ 996.59646, found 996.59717.

#### 3.2.5. N-Phthalimido-Gly-Leu-Leu-Leu-N-Me-Leu-Leu-Si(CH_3_)_3_ (**13**)

White solid (yield 73%). ^1^HNMR(CDCl_3_) ***δ***: 0.05~0.19 (m, 9H, SiMe_3_), 0.88~0.97 (m, 30H, CH_3_), 1.26~1.30 (m, 5H, CH(CH_3_)_2_),1.58~1.67 (m, 10H, CH_2_CH(CH_3_)_2_), 2.79~3.02 (m, 2H, CH_2_SiMe_3_), 3.22~3.27 (m, 3H, NCH_3_), 4.21~5.23 (m, 9H, CHCH_2_CH(CH_3_)_2_ and NCH_2_CO and CH_2_Ph), 7.26~7.40 (m, 5H, ArH), 7.72~7.89 (m, 4H, Phthaloyl); ^13^CNMR(CDCl_3_ ***δ***:−1.3, 21.8, 22.6, 22.6, 22.7, 232, 23.2, 23.5, 24.6, 24.7, 24.8, 30.9, 39.0, 441.6, 42.0, 42.2, 42.7, 46.9, 51.3, 51.7, 53.2, 53.5, 61.6, 123.4, 126.7, 127.7, 128.9, 132.0, 132.3, 136.7, 166.1, 167.7, 167.8, 170.5, 171.4, 173.1. HRMS (ESI) *m*/*z* calcd for C_52_H_81_N_7_O_8_SiNa^+^ (M + Na)^+^ 982.58081, found 982.58154.

#### 3.2.6. N-Phthalimido-Gly-Leu-N-Me-Leu-Leu-Leu-Leu-Si(CH_3_)_3_ (**14**)

White solid (yield 68%). ^1^HNMR(CDCl_3_) ***δ***: 0.05~0.15 (m, 7H, SiMe_3_), 0.16~0.22 (m, 2H, SiMe_3_),0.91~1.01 (m, 30H, CH_3_), 1.36~1.43 (m, 5H, CH(CH_3_)_2_),1.63~1.94 (m, 10H, CH_2_CH(CH_3_)_2_), 3.09~3.14 (m, 3H, NCH_3_), 3.14~3.33 (m, 2H, CH_2_SiMe_3_), 4.14~5.25 (m, 9H, CHCH_2_CH(CH_3_)_2_ and NCH_2_CO and CH_2_Ph), 7.27~7.38 (m, 4H, ArH), 7.90~7.91 (m, 5H, Phthaloyl); ^13^CNMR(CDCl_3_) ***δ***: −2.0, 22.4, 22.6, 22.8, 22.9, 22.9, 23.1, 23.2, 24.2, 24.4, 24.7, 25.6, 30.8, 28.4, 40.6, 41.5, 41.8, 42.2, 46.6, 47.3, 50.5, 51.0, 53.0, 60.5, 123.0, 126.9, 128.4, 128.7, 132.3, 134.2, 137.1, 167.4, 170.0, 170.9, 171.5, 171.8, 171.9, 172.2. HRMS (ESI) *m*/*z* calcd for C_52_H_81_N_7_O_8_SiNa^+^ (M + Na)^+^ 982.58081, found 982.58148.

#### 3.2.7. N-Phthalimido-Gly-Leu-Leu-Leu-N-Me-Leu-Leu-Si(CH_3_)_3_ (**15**)

White solid (yield 77%). ^1^HNMR(CDCl_3_) ***δ***: 0.05~0.15 (m, 7H, SiMe_3_), 0.16~0.20 (m, 2H, SiMe_3_), 0.87~0.96 (m, 30H, CH_3_), 1.27~1.29 (m, 5H, CH(CH_3_)_2_),1.47~1.86 (m, 10H, CH_2_CH(CH_3_)_2_), 2.79~2.83 (m, 3H, NCH_3_), 3.22~3.26 (m, 2H, CH_2_SiMe_3_), 4.39~5.30 (m, 9H, CHCH_2_CH(CH_3_)_2_ and NCH_2_CO and CH_2_Ph), 7.30~7.40 (m, 5H, ArH), 7.74~7.76 (m, 2H, Phthaloyl), 7.80~7.90 (m, 2H, Phthaloyl); ^13^CNMR(CDCl_3_) ***δ***: -1.3, 22.6, 22.6, 22.7, 22.7, 23.1, 23.3, 23.5, 24.6, 24.7, 24.8, 30.9, 39.0, 40.6, 41.5, 41.6, 46.9, 50.9, 51.3, 51.7, 53.2, 53.5, 61.4, 123.4, 126.7, 127.7, 128.9, 132.3, 133.9, 134.2, 136.7, 166.1, 167.8, 170.5, 171.2, 171.4, 171.8. HRMS (ESI) *m*/*z* calcd for C_52_H_81_N_7_O_8_SiNa^+^ (M + Na)^+^ 982.58081, found 982.58118.

#### 3.2.8. N-Phthalimido-Gly-Leu-N-Me-Leu-Leu-Leu-Leu-Si(CH_3_)_3_ (**16**)

White solid (yield 74%). ^1^HNMR(CDCl_3_) ***δ***: 0.05~0.13 (m, 6H, SiMe_3_), 0.14~0.18 (m, 3H, SiMe_3_),0.89~0.98 (m, 30H, CH_3_), 1.27~1.45 (m, 5H, CH(CH_3_)_2_),1.54~1.76 (m, 10H, CH_2_CH(CH_3_)_2_), 2.98~3.01 (m, 2H, CH_2_SiMe_3_), 3.16~3.21 (m, 3H, NCH_3_), 4.17~5.20 (m, 9H, CHCH_2_CH(CH_3_)_2_ and NCH_2_CO and CH_2_Ph), 7.22~7.35 (m, 5H, ArH), 7.71~7.72 (m, 2H, Phthaloyl), 7.85~7.86 (m, 2H, Phthaloyl); ^13^CNMR(CDCl_3_) ***δ***: −1.3, 22.6, 22.6, 22.7, 22.7, 23.1, 23.3, 23.5, 24.6, 24.7, 24.8, 30.9, 39.0, 40.6, 41.5, 41.6, 46.9, 50.9, 51.3, 51.7, 53.2, 53.5, 61.4, 123.4, 126.7, 127.7, 128.9, 132.3, 133.9, 134.2, 136.7, 166.1, 167.8, 170.5, 171.2, 171.4, 171.8. HRMS (ESI) *m*/*z* calcd for C_52_H_81_N_7_O_8_SiNa^+^ (M + Na)^+^ 982.58081, found 982.58148.

### 3.3. Preparation of Cyclic Peptides **1**–**8**

A total of 0.5 g of linear peptide 9 was dissolved in methanol (250 mL) and purged with nitrogen for 15 min. The solution was cooled by circulating water in a cylindrical glass jacket around the photoreactor and was irradiated with UV light (Pyrex tube filtered light λ > 290 nm) for 30 min to generate bicyclopeptides 4a-4i in methanol as a hydrogen donor and because the polar solvation effect would be helpful for the stability of the leaving group SiMe_3_^+^. The resulting reaction mixture was purified by column chromatography to give 3-hydroxy-isoindolinone-cyclopeptides (**1**–**8**).

#### 3.3.1. 3-Hydroxy-isoindolinone-cyclo-(Gly-Leu-N-Me-Leu-Leu-N-Me-Leu-Leu) (**1**)

White solid (yield 51%). ^1^HNMR(CDCl_3_) ***δ***: 0.90~0.99 (m, 30H, CH_3_), 1.28~1.31 (m, 5H, CH(CH_3_)_2_),1.44~1.66 (m, 10H, CH_2_CH(CH_3_)_2_), 2.92~3.26 (m, 6H, NCH_3_), 3.48~3.57 (m, 2H, NCH_2_C(OH)), 4.13~4.99 (m, 9H, CHCH_2_CH(CH_3_)_2_ and NCH_2_CO and CH_2_Ph), 7.24~7.39 (m, 5H, ArH), 7.54~7.61 (m, 4H, Phthaloyl); ^13^CNMR(CDCl_3_) ***δ***:21.5, 22.0, 22.2, 22.7, 22.8, 23.0, 23.4, 24.5, 24.6, 24.7, 24.8, 29.7, 38.4, 40.6, 46.8, 47.2, 49.2, 49.8, 51.7, 60.2, 63.1, 89.2, 122.0, 125.9, 126.4, 127.8, 128.9, 129.0, 130.0, 137.0, 142.7, 169.0, 170.7, 172.7, 174.9. HRMS (ESI) *m*/*z* calcd for C_50_H_75_N_7_O_8_Na^+^ (M + Na)^+^ 924.55693, found 924.55750.

#### 3.3.2. 3-Hydroxy-isoindolinone-cyclo-Gly-Leu-N-Me-Leu-Leu-N-Me-Leu-Leu (**2**)

White solid (yield 48%). ^1^HNMR(CDCl_3_) ***δ***: 0.73~1.12 (m, 30H, CH_3_), 1.30~1.36 (m, 5H, CH(CH_3_)_2_), 1.45~1.73 (m, 10H, CH_2_CH(CH_3_)_2_), 2.77~3.18 (m, 6H, NCH_3_), 3.48~3.80 (m, 2H, NCH_2_C(OH)), 4.17~4.22 (m, 2H, NCH_2_CO), 4.83~5.39 (m, 7H, CHCH_2_CH(CH_3_)_2_ and CH_2_Ph), 7.25~7.37 (m, 5H, ArH), 7.54~7.63 (m, 4H, Phthaloyl); ^13^CNMR(CDCl_3_)***δ***: 20.7, 21.4, 21.5, 21.7, 22.4, 22.8, 23.2, 23.5, 23.5, 24.6, 24.7, 24.8, 25.6, 29.6, 31.7, 43.2, 43.6, 44.0, 47.7, 48.2, 48.6, 48.8, 50.4, 52.5, 53.7, 55.3, 89.0, 121.6, 126.0, 126.1, 128.4, 128.9, 129.8, 130.0, 132.7, 136.9, 147.0, 168.1, 169.8, 170.3, 170.7, 171.6, 173.9, 175.7. HRMS (ESI) *m*/*z* calcd for C_50_H_75_N_7_O_8_Na^+^ (M + Na)^+^ 924.55693, found 924.55756.

#### 3.3.3. 3-Hydroxy-isoindolinone-cyclo-Gly-Leu-N-Me-Leu-Leu-N-Me-Leu-Leu (**3**)

White solid (yield 45%). ^1^HNMR(CDCl_3_) ***δ***: 0.69~1.19 (m, 30H, CH_3_), 1.44~1.53 (m, 5H, CH(CH_3_)_2_), 1.57~1.82 (m, 10H, CH_2_CH(CH_3_)_2_), 3.11~3.31 (m, 6H, NCH_3_), 3.73~3.84 (m, 2H, NCH_2_C(OH)), 4.14~4.16 (m, 2H, NCH_2_CO), 4.92~5.31 (m, 7H, CHCH_2_CH(CH_3_)_2_ and CH_2_Ph), 7.30~7.40 (m, 5H, ArH), 7.48~7.56 (m, 4H, Phthaloyl); ^13^CNMR(CDCl_3_)***δ***: 20.9, 21.0, 21.4, 21.5, 21.7, 22.3, 22.7, 23.1, 23.4, 23.5, 24.8, 25.6, 29.6, 31.7, 40.6, 43.2, 47.7, 48.2, 48.2, 48.3, 50.4, 50.7, 52.5, 55.3, 67.3, 70.8, 89.0, 121.6, 127.8, 128.4, 128.8, 128.9, 129.5, 130.0, 132.1, 132.6, 136.9, 167.6, 168.0, 168.7, 170.3, 170.7, 175.7. HRMS (ESI) *m*/*z* calcd for C_50_H_75_N_7_O_8_Na^+^ (M + Na)^+^ 924.55693, found 924.55750.

#### 3.3.4. 3-Hydroxy-isoindolinone-cyclo-Gly-Leu-N-Me-Leu-Leu-N-Me-Leu-Leu (**4**)

White solid (yield 43%). ^1^HNMR(CDCl_3_) ***δ***: 0.88~1.03 (m, 30H, CH_3_), 1.26~1.29 (m, 5H, CH(CH_3_)_2_), 1.44~1.69 (m, 10H, CH_2_CH(CH_3_)_2_), 2.91~3.19 (m, 6H, NCH_3_), 4.13~4.15 (m, 4H, NCH_2_C(OH)), 4.43~5.32 (m, 7H, CHCH_2_CH(CH_3_)_2_ and CH_2_Ph), 7.29~7.37 (m, 5H, ArH), 7.50~7.60 (m, 2H, Phthaloyl), 7.76~7.88 (m, 2H, Phthaloyl); ^13^CNMR(CDCl_3_) ***δ***: 21.5, 21.8, 22.0, 22.2, 22.7, 22.9, 23.4, 23.7, 24.8, 24.8, 25.0, 25.1, 29.6, 30.4, 39.1, 38.4, 39.7, 40.6, 40.9, 42.5, 46.7, 49.2, 49.8, 60.4, 67.1, 89.1, 123.6, 125.9, 126.4, 128.9, 130.0, 130.6, 132.1, 134.1, 147.2, 167.0, 167.7, 169.0, 170.2, 171.0, 172.7, 173.8. HRMS (ESI) *m*/*z* calcd for C_50_H_75_N_7_O_8_Na^+^ (M + Na)^+^ 924.55693, found 924.55688.

#### 3.3.5. 3-Hydroxy-isoindolinone-cyclo-Gly-Leu-Leu-Leu-N-Me-Leu-Leu (**5**)

White solid (yield 24%). ^1^HNMR(CDCl_3_) ***δ***: 0.79~1.08 (m, 30H, CH_3_), 1.28~1.32 (m, 5H, CH(CH_3_)_2_), 1.42~1.81 (m, 10H, CH_2_CH(CH_3_)_2_), 3.03~3.06 (m, 3H, NCH_3_), 4.10~4.31 (m, 4H, NCH_2_C(OH)), 4.74~5.01 (m, 7H, CHCH_2_CH(CH_3_)_2_ and CH_2_Ph), 7.29~7.37 (m, 6H, ArH), 7.60~7.65 (m, H, Phthaloyl), 7.74~7.88 (m, 2H, Phthaloyl); ^13^CNMR(CDCl_3_)***δ***: 21.5,21.7, 22.7, 22.8, 22.9, 22.9, 23.3, 24.6, 24.7, 25.0, 29.5, 37.1, 38.1, 39.8, 41.1, 41.3, 43.5, 48.0, 50.7, 53.4, 54.5, 54.6, 58.7, 58.8, 90.6, 124.1, 125.9, 129.0, 129.1, 129.2, 129.4, 130.5, 133.4, 134.9, 146.4, 169.8, 171.7, 172.7, 172.8, 174.3, 177.5. HRMS (ESI) *m*/*z* calcd for C_49_H_73_N_7_O_8_Na^+^ (M + Na)^+^ 910.54128, found 910.54150.

#### 3.3.6. 3-Hydroxy-isoindolinone-cyclo-Gly-Leu-N-Me-Leu-Leu-Leu-Leu (**6**)

White solid (yield 25%). ^1^HNMR(CDCl_3_) ***δ***: 0.93~1.05 (m, 30H, CH_3_), 1.28~1.46 (m, 5H, CH(CH_3_)_2_), 1.53~1.75 (m, 10H, CH_2_CH(CH_3_)_2_), 3.16~3.29 (m, 3H, NCH_3_), 4.13~4.19 (m, 4H, NCH_2_C(OH)), 4.49~4.74 (m, 7H, CHCH_2_CH(CH_3_)_2_ and CH_2_Ph), 7.29~7.66 (m, 9H, ArH); ^13^CNMR(CDCl_3_) ***δ***: 21.3, 22.3, 22.4, 23.1, 23.4, 24.5, 25.1, 25.3, 25.3, 29.7, 37.1, 39.6, 39.9, 40.4, 40.6, 44.3, 48.8, 49.1, 51.7, 54.2, 62.7, 65.8, 90.3, 123.9, 125.7, 127.9, 128.9, 129.0, 130.1, 130.5, 132.6, 135.7, 146.3, 168.3, 170.1, 170..6, 172.8, 172.9, 173.6, 174.0. HRMS (ESI) *m*/*z* calcd for C_49_H_73_N_7_O_8_Na^+^ (M + Na)^+^ 910.54128, found 910.54163.

#### 3.3.7. 3-Hydroxy-isoindolinone-cyclo-Gly-Leu-Leu-Leu-N-Me-Leu-Leu (**7**)

White solid (yield 28%). ^1^HNMR(CDCl_3_) ***δ***: 0.91~1.01 (m, 30H, CH_3_), 1.29~1.43 (m, 5H, CH(CH_3_)_2_), 1.53~1.89 (m, 10H, CH_2_CH(CH_3_)_2_), 3.08~3.22 (m, 3H, NCH_3_), 4.11~4.17 (m, 4H, NCH_2_C(OH)), 4.53~5.97 (m, 7H, CHCH_2_CH(CH_3_)_2_ and CH_2_Ph), 7.29~7.39 (m, 5H, ArH), 7.55~7.63 (m, 2H, Phthaloyl), 7.74~7.82 (m, 2H, Phthaloyl); ^13^CNMR(CDCl_3_) ***δ***: 22.7, 22.9, 23.1, 23.3, 23.4, 23.5, 24.6, 24.7, 24.9, 31.9, 37.1, 40.2, 40.4, 40.7, 40.9, 43.7, 47.7, 50.7, 51.7, 53.4, 53.4, 54.4, 54.6, 58.8, 89.0, 125.9, 126.4, 127.4, 129.0, 130.0, 130.3, 131.9, 135.8, 147.2, 168.6, 169.7, 171.8, 172.2, 172.9, 173.1, 173.2. HRMS (ESI) *m*/*z* calcd for C_49_H_73_N_7_O_8_Na^+^ (M + Na)^+^ 910.54128, found 910.54193.

#### 3.3.8. 3-Hydroxy-isoindolinone-cyclo-Gly-Leu-N-Me-Leu-Leu-Leu-Leu (**8**)

White solid (yield 23%). ^1^HNMR(CDCl_3_) ***δ***: 0.70~1.05 (m, 30H, CH_3_), 1.28~1.34 (m, 5H, CH(CH_3_)_2_), 1.46~1.91 (m, 10H, CH_2_CH(CH_3_)_2_), 2.77~3.14 (m, 3H, NCH_3_), 3.90~4.20 (m, 4H, NCH_2_C(OH)), 4.55~5.97 (m, 7H, CHCH_2_CH(CH_3_)_2_ and CH_2_Ph), 7.22~7.57 (m, 9H, ArH); ^13^CNMR(CDCl_3_) ***δ***: 21.4, 21.5, 21.7, 22.3, 22.4, 23.0, 23.1, 23.4, 24.4, 24.5, 25.0, 25.2, 25.3, 31.9, 37.1, 37.8, 39.7, 40.0, 40.6, 44.3, 48.6, 48.9, 51.8, 53.3, 54.2, 65.7, 90.3, 121.7, 123.8, 125.7, 127.8, 129.0, 130.1, 130.6, 132.5, 135.8, 146.3, 168.4, 169.9, 170.4, 171.1, 172.7, 173.6, 173.8. HRMS (ESI) *m*/*z* calcd for C_50_H_75_N_7_O_8_Na^+^ (M + Na)^+^ 924.55693, found 924.55688.

### 3.4. MTT Experiment

Samples were dissolved in DMSO and diluted into different concentrations (0, 9.375, 12.25, 18.75, 25, 37.5, 50, 75, 100 and 250 μM) and stored at 4 °C. Cells in the logarithmic growth phase were digested with trypsin and then seeded in 96-well plates at a density of 2 × 10^4^ cells/well in 100 μL of culture medium. The cells were treated with different concentrations of the tested compound, and a blank control group without the drug was also set up. Six wells were repeated for each dose. After 48 h of co-incubation, MTT solution (100 μL, 0.5 mg/mL) was added, followed by incubation for another 4 h. After discarding the supernatant, 100 μL of DMSO was added per well, and the absorbance (OD) values were measured at 490 nm using an enzyme marker. Cell viability was calculated by the formula: Cell viability (%) = A_s_/A_b_ × 100% (where A_s_ is the absorbance value of the experimental group and A_b_ is the absorbance value of the blank group). The obtained data were processed using SPSS19 software to obtain IC_50_ values.

### 3.5. Detecting Apoptosis by Flow Cytometry

HepG-2 cells were seeded in a 6-well plate (2 × 10^5^ cells per well). The different concentrations of compound **8** (0, 5.5, 11, 22 μg/mL) were added and incubated for 48 h to induce apoptosis. The collected cells were centrifuged (2000 r/min) for 5 min and resuspended in PBS (200 μL). Then, 5 μL of Annexin V-FITC was added and incubated for 10 min at room temperature away from light. Finally, 5 µL propidium iodide solution was added and incubated for 5 min away from light. The fluorescence intensity was detected by flow cytometry and analyzed by utilizing FlowJo_V10 software (FlowJo X 10.0.7r2, BD Biosciences, Franklin Lakes, NJ, USA).

### 3.6. Detecting the Effect on the Cell Cycle by Flow Cytometry

HepG-2 cells were inoculated in 6-well plates and incubated for 24 h. Then, different concentrations of compound **8** were added to the wells and incubated for the required time. The collected cells in the experimental and control groups were digested with trypsin, centrifuged (1000 r/min, 5 min), resuspended in PBS and fixed by 70% ice ethanol (3 mL) at −20 °C for 24 h. Then, cells were washed twice with PBS and resuspended in PBS (0.9 mL), then treated with 100 µL RNase (100 µg/mL) for 30 min at 37 °C. After centrifuging the mixture for 5 min, the cells were resuspended in 50 µg/mL PBS and stained with 600 µL of PI solution (Beyotime Biotechnology, Shanghai, China) for 60 min at 4 °C to stain. Finally, the physical properties of individual cells at different mitotic stages were analyzed by flow cytometry (BD Accuri C6, BD Biosciences, Franklin Lakes, NJ, USA).

### 3.7. Detecting the Effect on Mitochondrial Membrane Potential by Flow Cytometry

Cells in the logarithmic phase were seeded in 6-well plates and incubated for 24 h. After refreshing the culture medium, different concentrations of compound **8** (0, 5.5, 11, 22 μM) were added and co-incubated for 48 h. Meanwhile, the cells were also treated with 11 μM of compound **8** within different time periods (12 h, 24 h, 48 h). Then, the cells were collected by trypsin digestion and washed with phosphate-buffered solution. After centrifuging (1000 r/min) for 5 min, the obtained cell precipitate was suspended in PBS (100 µL); then, 100 µL of rhodamine 123 (1 µM) was added and incubated for 20 min. The cells were centrifuged, resuspended in 600 µL PBS, and analyzed by flow cytometry (maximum excitation wavelength 507 and maximum emission wavelength 529 nm). At least 10,000 events were counted in the experiment. 

### 3.8. Detecting the Effect on Intracellular Ca^2+^ Concentration

Cells incubated in 6-well plates were treated in a similar way as mentioned above, and then 100 µL of Fluo-3 AM (5 µM) was added and incubated for 1 h at 37 °C away from light. After rewashing the cells, the suspended precipitate in PBS was checked by flow cytometry (excitation at 488 nm and emission at 525 nm).

### 3.9. Lactate Dehydrogenase (LDH) Activity Assay

Lactate dehydrogenase activity of HepG-2 cells treated with compound **8** was examined by using the Lactate Dehydrogenase Kit (Beijing Solarbio Science & Technology, Beijing, China) according to the instructions for use. Cells in the logarithmic phase were seeded in a 96-well plate (2 × 10^5^ cells per well) and incubated for 24 h. The cells were divided into experimental and control groups. The experimental group cells were treated with different concentrations (37.5, 50, 75, 100, 150, and 200 µM of PBS solution) of compound **8** for 48 h. Then, cells were digested, centrifuged, resuspended in PBS and re-centrifuged (1000 r/min) in the same way as mentioned earlier. After that, 100 µL of LDH extract was added to each well. Then, the cells were sonicated for 10 s (repeated 30 times) and centrifuged (8000 r/min) for 10 min. The supernatant was taken and placed on ice for testing. A test tube and a control tube were set up at the same time. Then the other reagents in the Lactate Dehydrogenase Kit (Beyotime Biotechnology, Shanghai, China) were added according to the instructions, and the absorption of the mixture was measured by the spectrophotometer. Meanwhile, sodium pyruvate was dissolved in acetone to prepare the standard solutions at the concentrations of 0, 0.125, 0.25, 0.5, 1, and 2 μM. The absorption of the standard solution at 450 nm was measured to plot the standard curve. The previously measured value of each sample was substituted into the regression equation of the standard curve to calculate the y-value (μM), and the lactate dehydrogenase activity was calculated according to the equation: LDH_active_ (U/10^4^ cells) = 0.133 y.

### 3.10. Effect on Protein Levels of MDM2 and p53 by Western Blot

HepG-2 cells (5 × 10^6^ cells) in the medium were treated with different concentrations of compounds for the required time and were washed twice with ice-cold PBS. The cells were scraped into cell lysate buffer containing phenylmethanesulfonyl fluoride (PMSF) and transferred to a 1.5 mL centrifuge tube, then lysed for 20 min at 4 °C. After centrifuging (12,000 r/min) for 20 min, the supernatant was collected and measured using the BCA Protein Assay Kit (Beyotime Biotechnology Co. Ltd., Shanghai, China)). Protein samples were then electrophoresed on 12% SDS-polyacrylamide gels and transferred to polyvinylidene difluoride (PVDF) membranes. The membranes were then closed with 5% skim milk in Tris-buffered saline Tween 20 (TBST) (containing 50 mM Tris-HCl, 150 mM NaCl and 0.02% Tween-20) for 2 h. The membranes were co-incubated overnight at 4 °C with a primary Akt antibody (1:1000), followed by another incubation with the secondary antibody (1:5000). After that, the membranes were washed 5 times with TBST buffer (5 min each time). Finally, the chemiluminescent enhancer was added drop-wise, and the image was captured by the chemiluminescent imaging system. Β-actin was used as the control.

### 3.11. Theoretical Computational Details

The compounds were firstly drawn by ChemBio3D Ultra 14.0 (CambridgeSoft Corporation, Waltham, MA, USA) and optimized by the MM2 method. Then, the stable conformations were searched with the MMFF94 molecular mechanic force field. The obtained conformers were optimized at the DFT/B3LYP/6-31G (d, p) level, and the ECD was calculated by time-dependent DFT calculations on the lowest energy conformations (>5% population) at the level of TDDFT/ωb97xd/cc-pVDZ using a polarizable continuum model (PCM) to consider the solvent. ECD curves were simulated based on rotatory strengths by Specdis 1.71 [38]. The calculated spectra were UV-corrected for comparison to the experimental data. In the docking studies, the cyclopeptide molecules were firstly optimized by molecular mechanics (MM2) methods to establish the low energy conformation, which were then placed in the binding site of receptors to find the configuration with the lowest binding energy. A semi-flexible docking was used to study the binding mode. The amino acid residues from the protein (MDM2) were set as “rigid”, while the small molecules were set as “random” (flexible). The docking experiments were carried out by AutoDock 4.26 (The scripps research institute, La Jolla, CA, USA). The crystal structure of the target protein was obtained from the RCSB database. The hydrogen was added and charge calculated by AutoDock Tools 1.5.6 software (The scripps research institute, La Jolla, CA, USA) [39].

## 4. Conclusions

Various analogs of the natural cyclic peptide galaxamide containing different chiral and methyl-substituted structural fragments were synthesized by intramolecular photoinduced single electron transfer cyclization reactions, and their stereochemistry was studied by circular dichroism with the aid of theoretical calculations. Systematical in vitro experiments displayed that the galaxamide analogs with isoindolinone fragments in the skeleton could efficiently induce apoptosis, decrease mitochondrial membrane potential, increase intracellular Ca^2+^ concentration and inhibit lactate dehydrogenase activity, implying a promising inhibitory effect on the growth of HepG-2 cells. Molecular docking has disclosed that the introduction of isoindolinone into galaxamide could effectively improve the intermolecular interactions and facilitate the binding of cyclic peptide analogs to MDM2 protein. While the compounds discussed in this paper have relatively modest activity, the research has disclosed some useful guides for peptide-based drug design, that is, that methylation is not always effective in enhancing the bioactivity of small cyclic peptides, and also provided evidence that the broader molecular surface area would lead to stronger binding force, which might give useful clues for the study of protein-protein interactions.

## Figures and Tables

**Table 1:** IC_50_ values (μM) of different compounds on HepG-2, MDA-MB-231 and L929 cells.

Compounds	IC_50_ (μM)		
	HepG-2	MDA-MB-231	L929 ^[a]p^
**1**	38.4 ± 2.2	59.3 ± 2.3	>400
**2**	72.1 ± 5.8	67.6 ± 3.3	363.6 ± 15.6
**3**	90.9 ± 7.69	73.2 ± 2.8	>400
**4**	80.99 ± 4.29	95.3 ± 8.6	310.4 ± 20.1
**5**	28.2 ± 6.2	60.8 ± 8.2	153.1 ± 10.7
**6**	36.1 ± 2.2	67.6 ± 2.3	120.5 ± 5.3
**7**	30.4 ± 1.6	111.5 ± 9.0	>400
**8**	11.1± 0.7	41.7 ± 3.2	>400
Taxol	40.9 ± 5.2	-	140.6 ± 9.3

^[a]p^ L929 cells were used to evaluate the toxicity of cyclopeptides to normal cell lines.

**Table 2:** Molecular docking binding energy towards MDM2 protein (PDB: 3EQS) (kcal·mol^−1^).

Compounds	1	2	3	4	5	6	7	8
E_Binding_	−5.59	−5.87	−5.91	−5.5	−5.2	−6.43	−6.7	−10.33

## Data Availability

Not applicable.

## Supplementary Materials

The supplementary experiment details, including the NMR, MS, and docking information, can be found online at: https://www.mdpi.com/article/10.3390/md20060379/s1. Table S1 shows the absolute configurations, relative free energies and populations of the conformers as determined in methanol; Figure S1 shows the most stable low energy conformers of cyclic compounds in methanol (DFT/B3LYP/6-31G**); Table S2. Docking process parameter settings for those with relative high binding energy; Table S3. The forces between compound **4**, compound **8** and MDM2 protein obtained by molecular docking.
