# Supplementary material for: Photoinduced Synthesis of Methylated Marine Cyclopeptide Galaxamide Analogs with Isoindolinone as Anticancer Agents
Source: Mar Drugs. 2022 Jun 5;20(6):379. doi: 10.3390/md20060379 (PMC9227305; doi:10.3390/md20060379)
Supplement: Supplementary file 1 [file marinedrugs-20-00379-s001.zip › marinedrugs-1732655-supplementary.pdf]

Supporting Information

Photoinduced synthesis of methylated marine cyclopeptide Galaxamide analogs with isoindolinone as anticancer agents

Shimei Xiao, Zhiqiang Wang*, Huanli Zhang, Lei Zhao, Qingran Chang, Xiong Zhang, Rui Yan, Xiaodan Wu, and Yingxue Jin*

Key Laboratory for Photonic and Electronic Bandgap Materials, Ministry of Education, College of Chemistry & Chemical Engineering, Harbin Normal University, Harbin, 150025, China.

***Corresponding author.** *E-mail: wangzq@hrbnu.edu.cn (Z.W.); yenghak@hrbnu.edu.cn (Y.J.)*

Contents

1. Table S1. The absolute configurations, relative free energies and populations of the conformations as determined in methanol	S2
2. The most stable conformers of the prepared compounds in methanol	S2
3. Computational details and the docking	S3-S4
4. ^{1}H, ^{13}C-NMR and HRMS of linear peptides.	S4-S16
5. ^{1}H, ^{13}C-NMR, HRMS and HPLC spectra of and cyclic peptides.	S16-S32

1. **Table S1**. The absolute configurations, relative free energies and populations of the conformations as determined in methanol

Compounds	Stable conformers*	ΔG**	P%***	Determined absolute configuration of C-3
1	1S	0	100	***S***
2	2S-1	0	50.5	***S***
	3S-2	0.01	49.5	
3	3S-1	0	98.7	***S***
	3S-2	2.55	1.3	
4	4R-1	0	69.0	***R***
	4R-2	0.48	31.0	
5	5R-1	0	99.8	***R***
	5R-2	3.61	0.2	
6	6R-1	0.00	91.3	***R***
	6R-2	1.39	83.7	
7	7R-1	0	70.0	***R***
	7R-2	0.59	25.7	
	7R-3	1.65	4.3	
8	8R-1	0	8.03	***R***
	8R-2	0.83	19.7	

*See Fig.S1 for the structures of the most stable conformers.

**The unit for ΔG is kcal mol^{-1}.

***Populations are based on ΔG values.

2. The most stable conformers of the prepared compounds in methanol

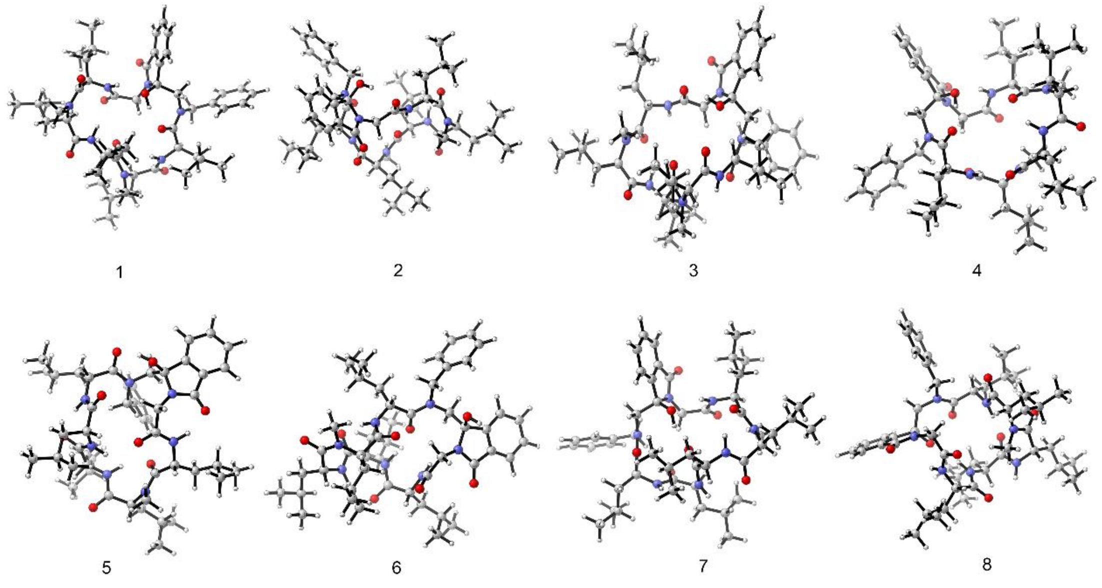

Fig. S1. The most stable low energy conformers of cyclic compounds in methanol (DFT/B3LYP/6-31G**).

3. Computational details

Conformational analysis was performed by fixing the absolute configuration of C-3 with the MMFF94 method. The obtained conformers were optimized at the DFT/B3LYP/6-31G(d,p) level by Gaussian 09 (Gaussian 09, Revision C.01; Gaussian, Inc.: Wallingford, CT, 2010.). The excitation energy (denoted by wavelength in nm) and rotatory strength R of the lowest energy conformations (>5% population) were calculated at the level of TDDFT/ωB97XD/cc-PVDZ using polarizable continuum model (PCM) to consider the solvent effect. ECD curves were obtained based on rotatory strengths using half bandwidth of 0.30~0.40 eV by Specdis 1.71(T. Bruhn, et. al. *Chirality*, 2013, **25**, 243). UV correction was used to facilitate comparison between the calculated and experimental data.

Docking experiments were performed by AutoDock 4.26 (ADT). The structure of target protein MDM2 was downloaded from the RCSB protein database (RCSB PDB, http://www.pdb.org/), and the modified ligand and water were separated using PyMol software. The cyclic structure was based on the optimized structure at the level of DFT/B3LYP/6-31G(d,p). The hydrogen was added and charge calculated by AutoDock Tools 1.5.6 software, and the docking results were processed by Discovery Studio 2019 Client. The docking parameters were shown in supplements Table S1 and S2.

Table S2. Docking process parameter settings for those with relative high binding energy.

Parameter	4R	8R
Grid box (nm)	60 × 60 × 60	60 × 60 × 60
spacing	0.375	0.375
grid center x	-7.442	-7.442
grid center y	0.621	0.621
grid center z	6.892	6.892
GA runs	20	20
population size	150	150

Table S3. The forces between compound **4**, compound **8** and MDM2 protein obtained by molecular docking.

Compounds	Vander Waals	Conventional H-bond	Carbon H-bond	Alkyl, π-Alkyl	π-Sigma,	Amide-Pi Stacked
**4**	Leu^{54}, Leu^{57}, Gly^{58}, Gln^{72}, His^{73}, Val^{75}, Phe^{86}, Ile^{103}		Gly^{58}	Phe^{55}, Ile^{61}, Met^{62}, Val^{93} , Ile^{99}	Tyr^{67}, His^{96}	
**8**	Lys^{51}, Phe^{55}, Gly^{58}, Gln^{72}, Phe^{91}, Tyr^{100}, Ile^{103}	His^{96}	His^{73}	Leu^{57}, Val^{57}, Ile^{61}, Met^{62}, Tyr^{67}, Val^{93}, Ile^{99}	Val^{93}	Leu^{54}

4. ^{1}H, ^{13}C-NMR and HRMS of linear peptides.

(1) ^{1}H, ^{13}C-NMR and HRMS of **9.**

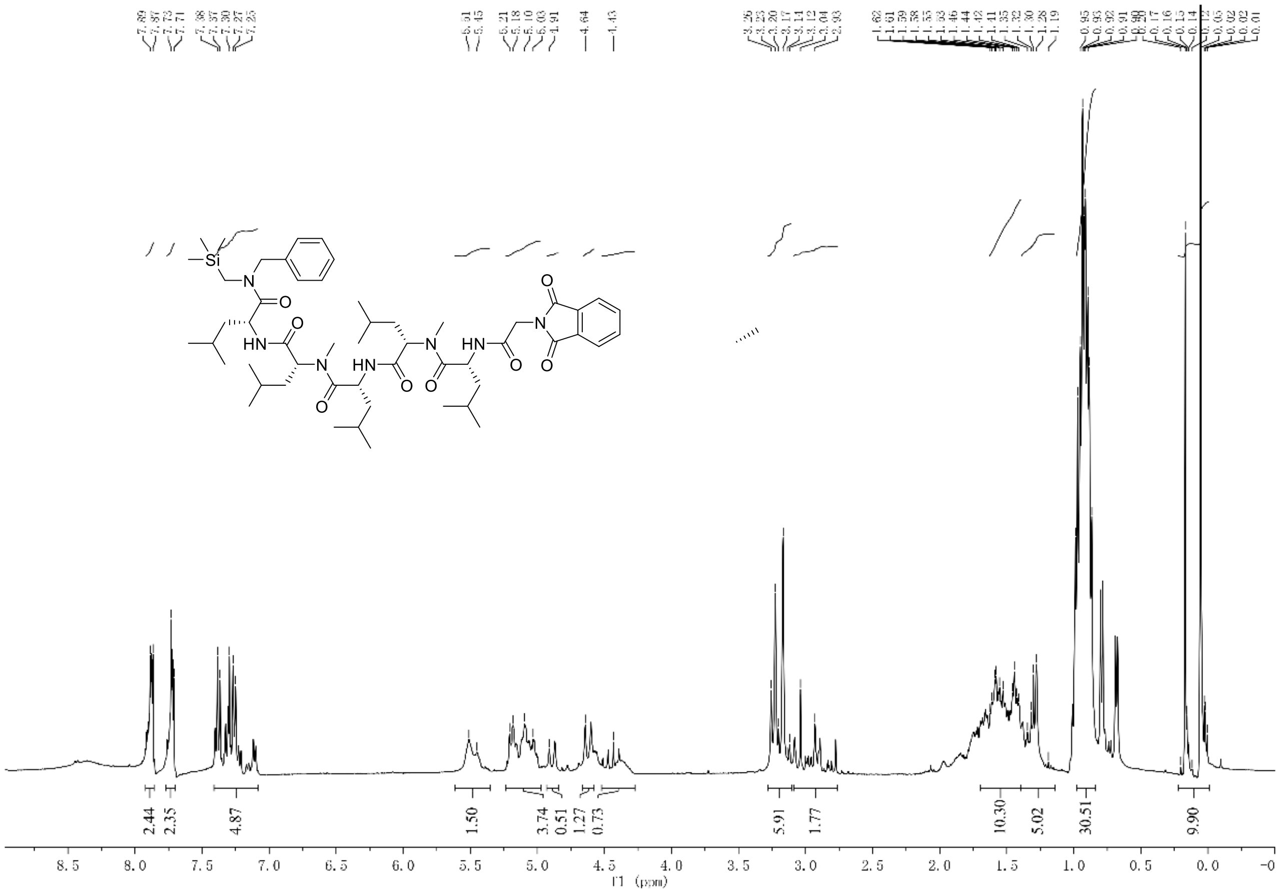

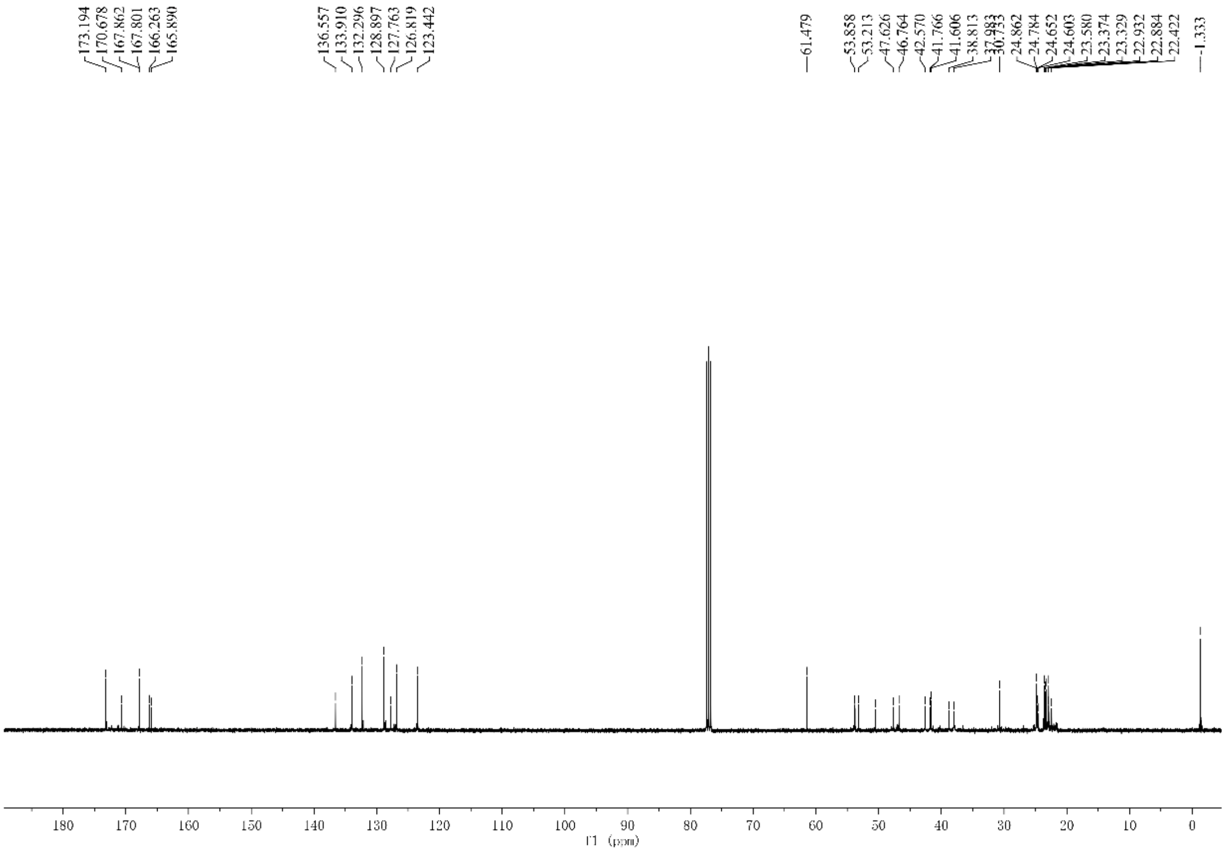
173.194
170.678
167.862
167.801
166.263
165.890
136.557
133.910
132.296
128.897
127.763
126.819
123.442
61.479
53.858
53.213
47.626
46.764
42.570
41.766
41.606
38.813
24.862
24.784
24.652
24.603
23.580
23.374
23.329
22.932
22.884
22.422
1.333
180 170 160 150 140 130 120 110 100 90 80 70 60 50 40 30 20 10 0
f1 (ppm)

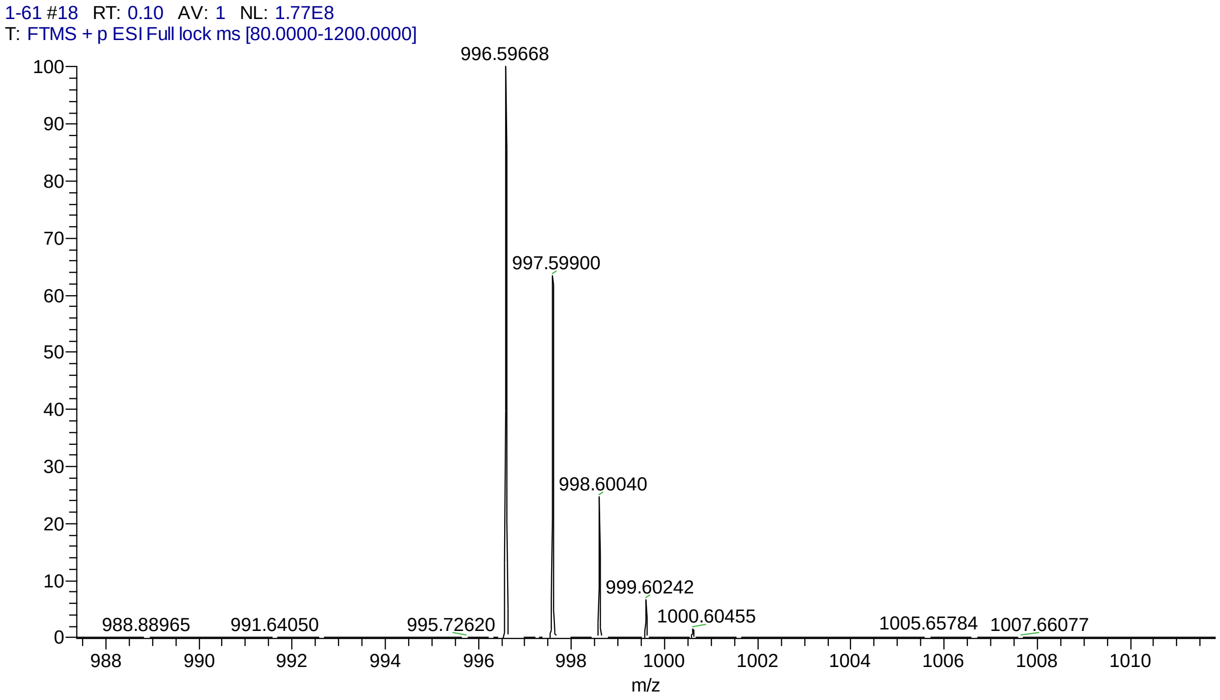
1-61 #18 RT: 0.10 AV: 1 NL: 1.77E8
T: FTMS + p ESI Full lock ms [80.0000-1200.0000]
996.59668
997.59900
998.60040
999.60242
1000.60455
988.88965
991.64050
995.72620
1005.65784
1007.66077
m/z

(2) ^{1}H, ^{13}C-NMR and HRMS of **10**.

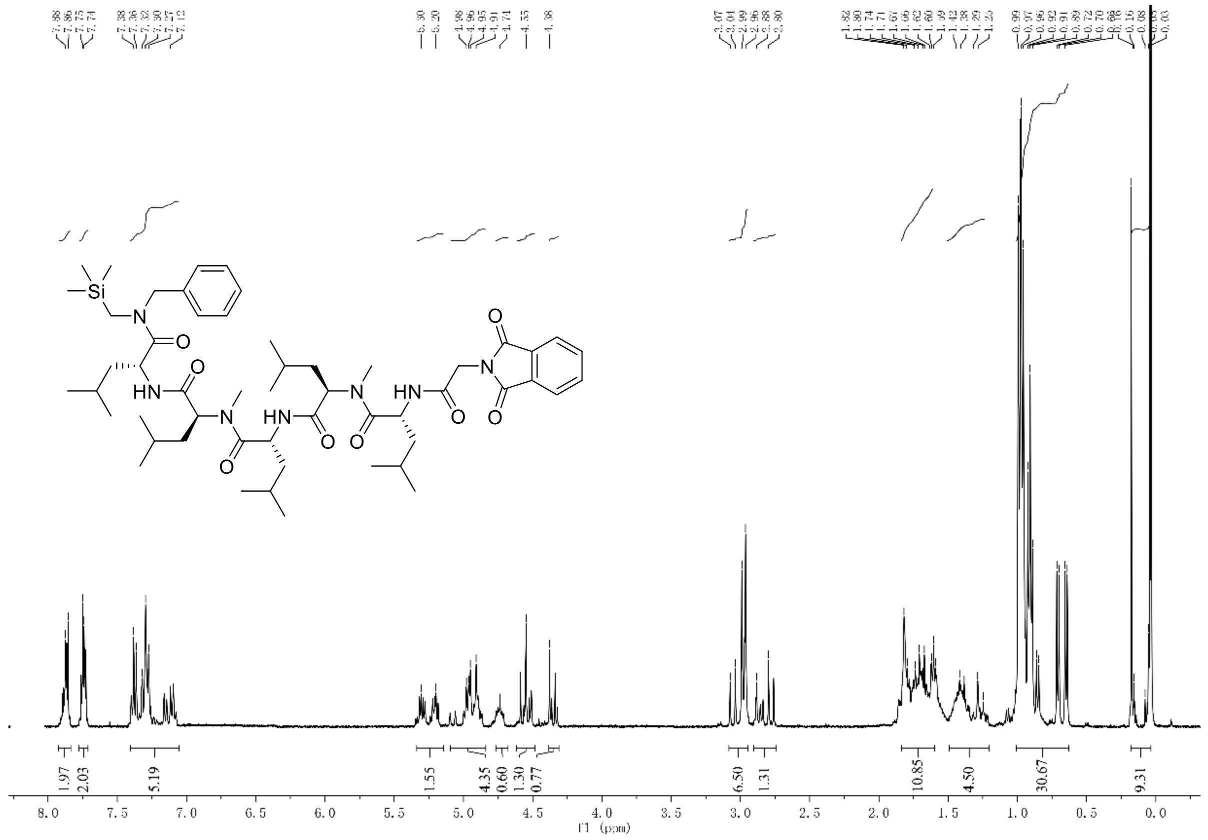

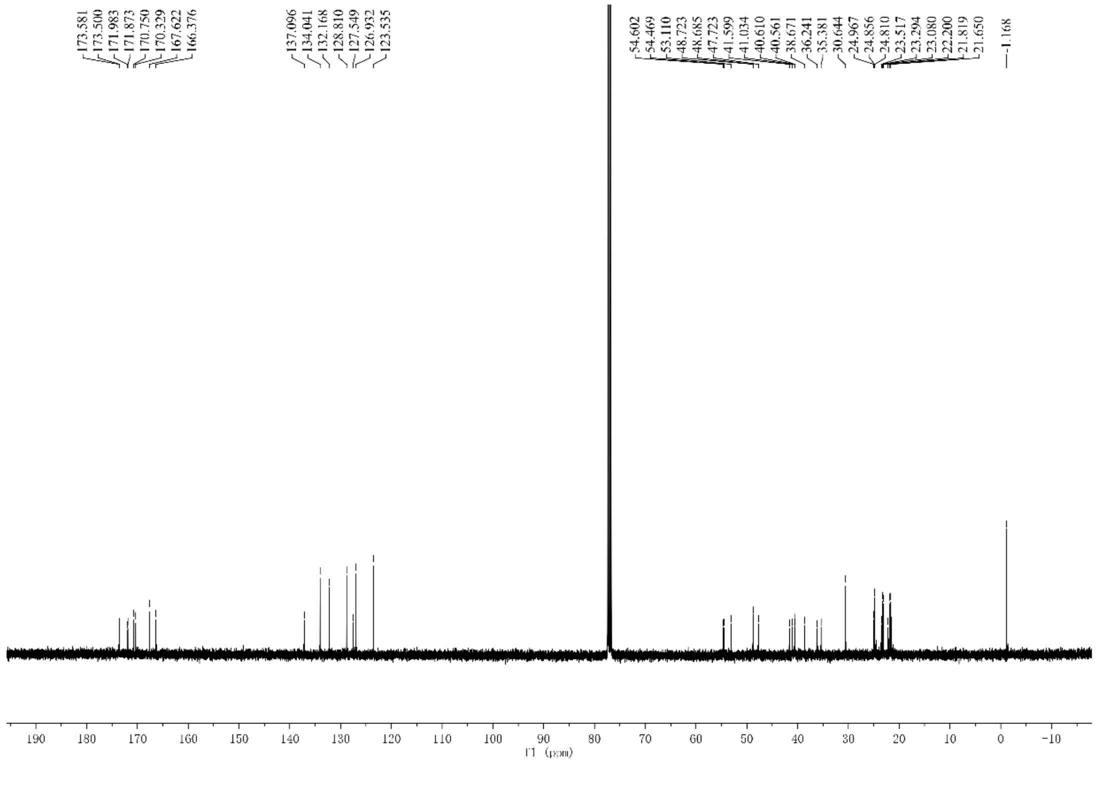

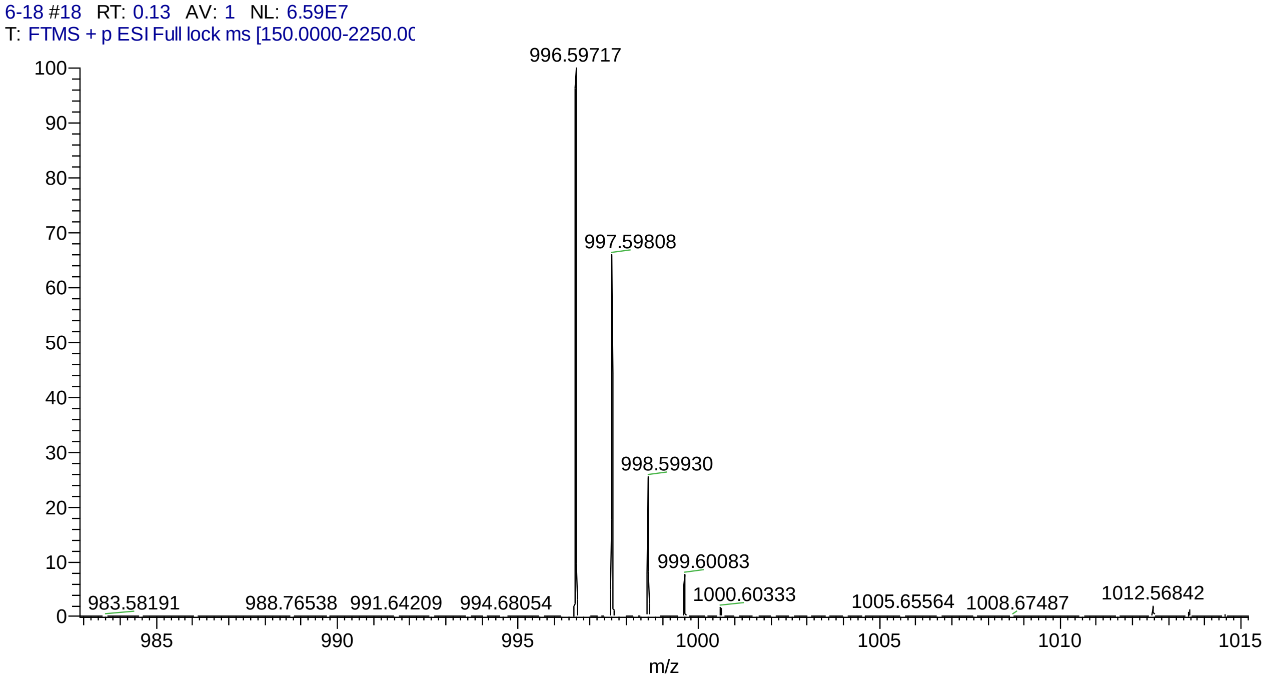

(3) ^{1}H, ^{13}C-NMR and HRMS of **11**.

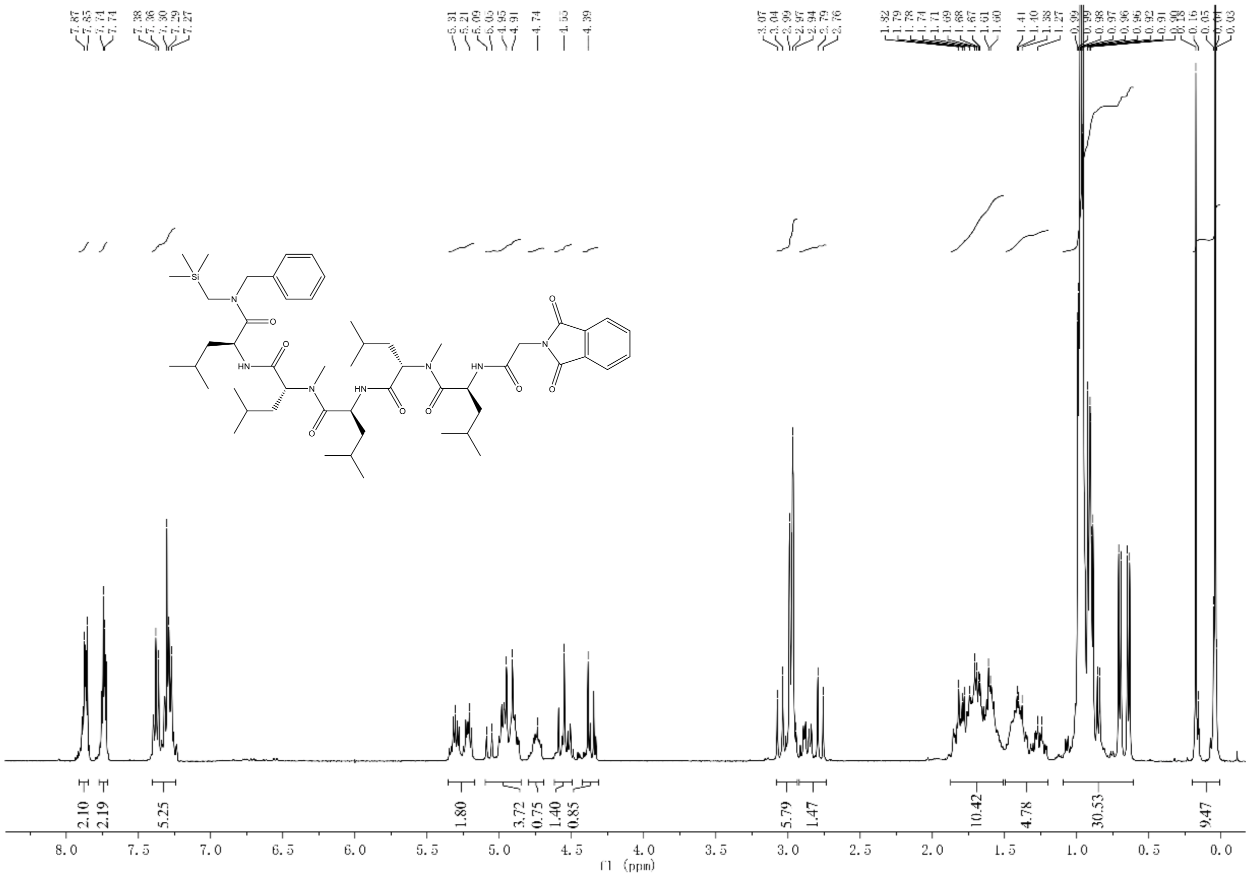

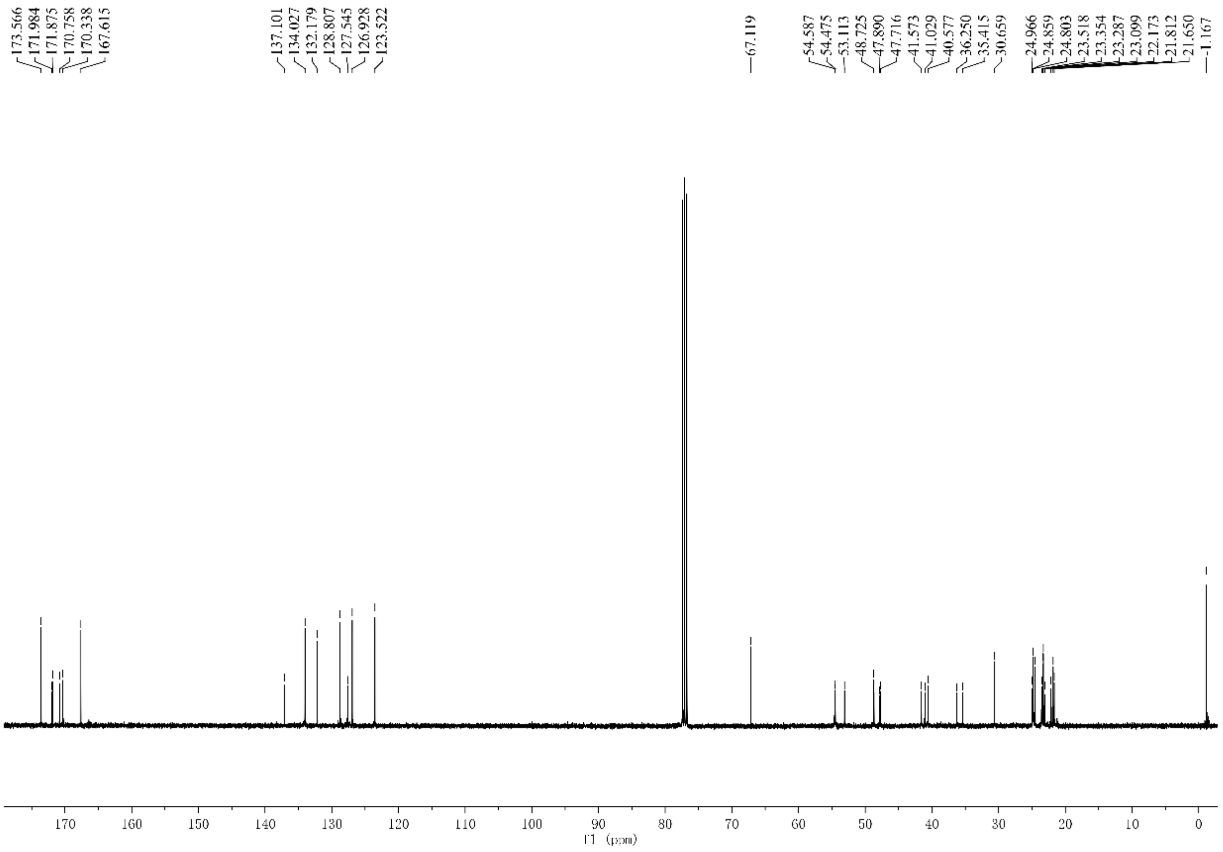
173.566
171.984
171.875
170.758
170.338
167.615
137.101
134.027
132.179
128.807
127.545
126.928
123.522
67.119
54.587
54.475
53.113
48.725
47.890
47.716
41.573
41.029
40.577
36.250
35.415
30.659
24.966
24.859
24.803
23.518
23.354
23.287
23.099
22.173
21.812
21.650
-1.167
f1 (ppm)

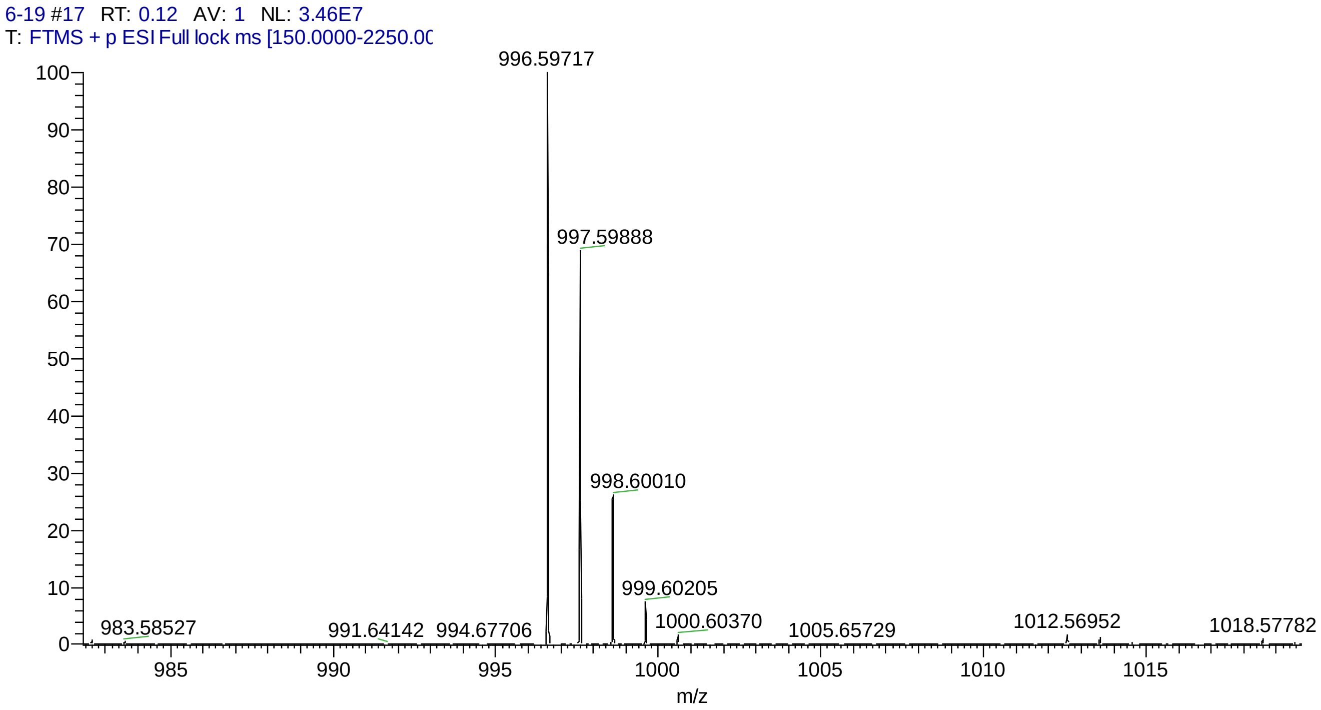
6-19 #17 RT: 0.12 AV: 1 NL: 3.46E7
T: FTMS + p ESI Full lock ms [150.0000-2250.00
996.59717
997.59888
998.60010
999.60205
1000.60370
983.58527
991.64142
994.67706
1005.65729
1012.56952
1018.57782
m/z

(4) ^{1}H, ^{13}C-NMR and HRMS of **12**.

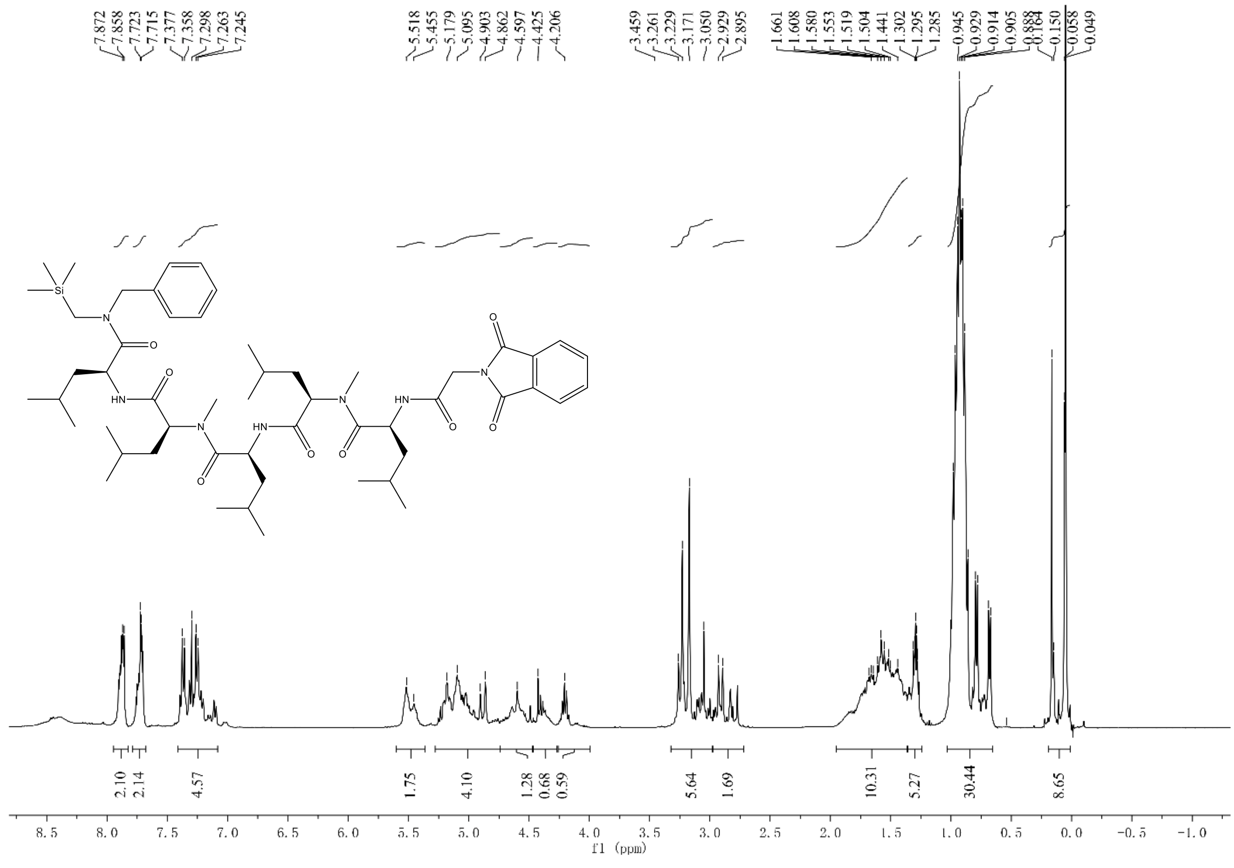

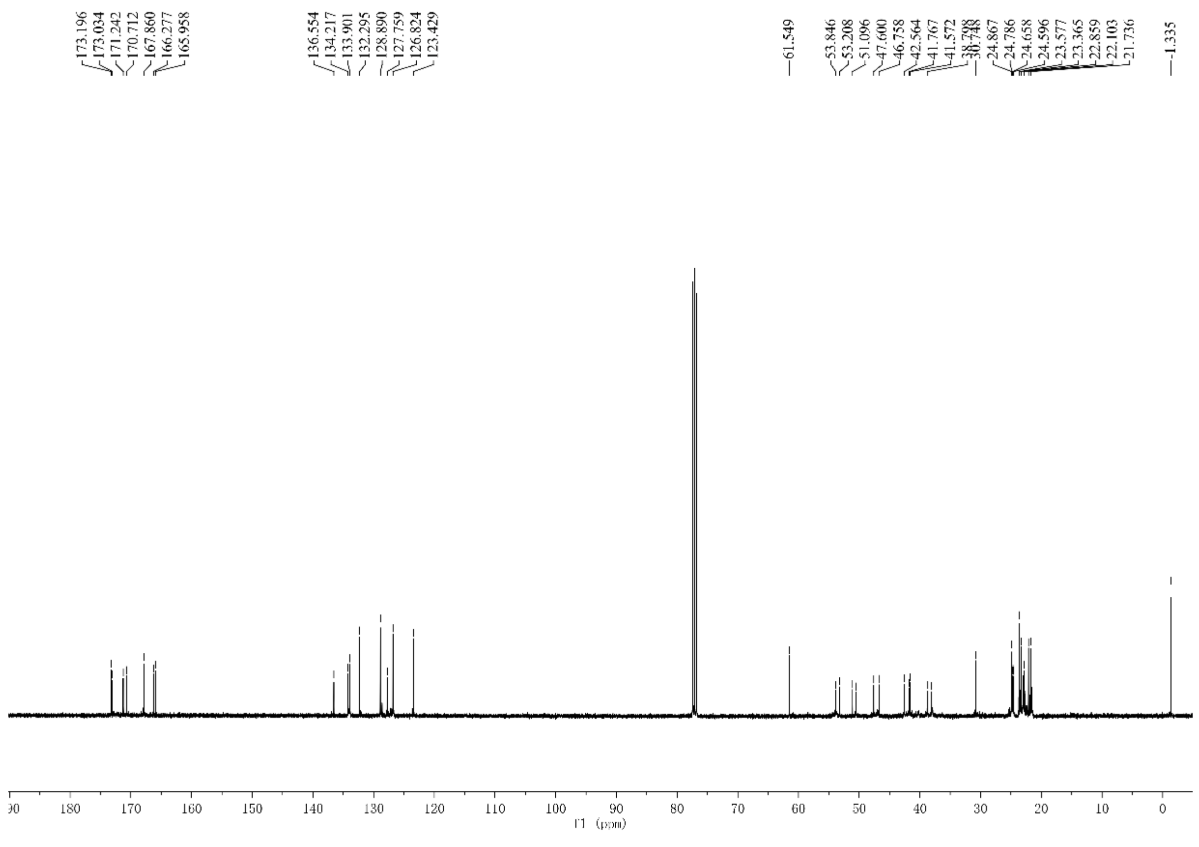

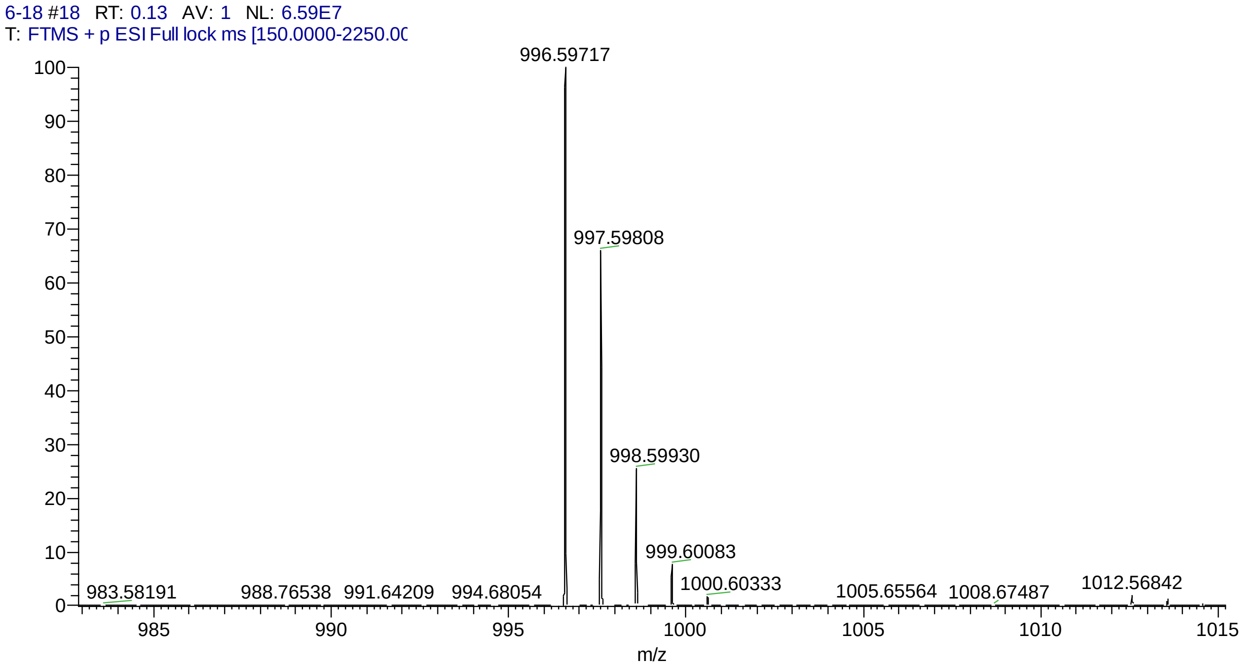

(5) ^{1}H, ^{13}C-NMR and HRMS of **13**.

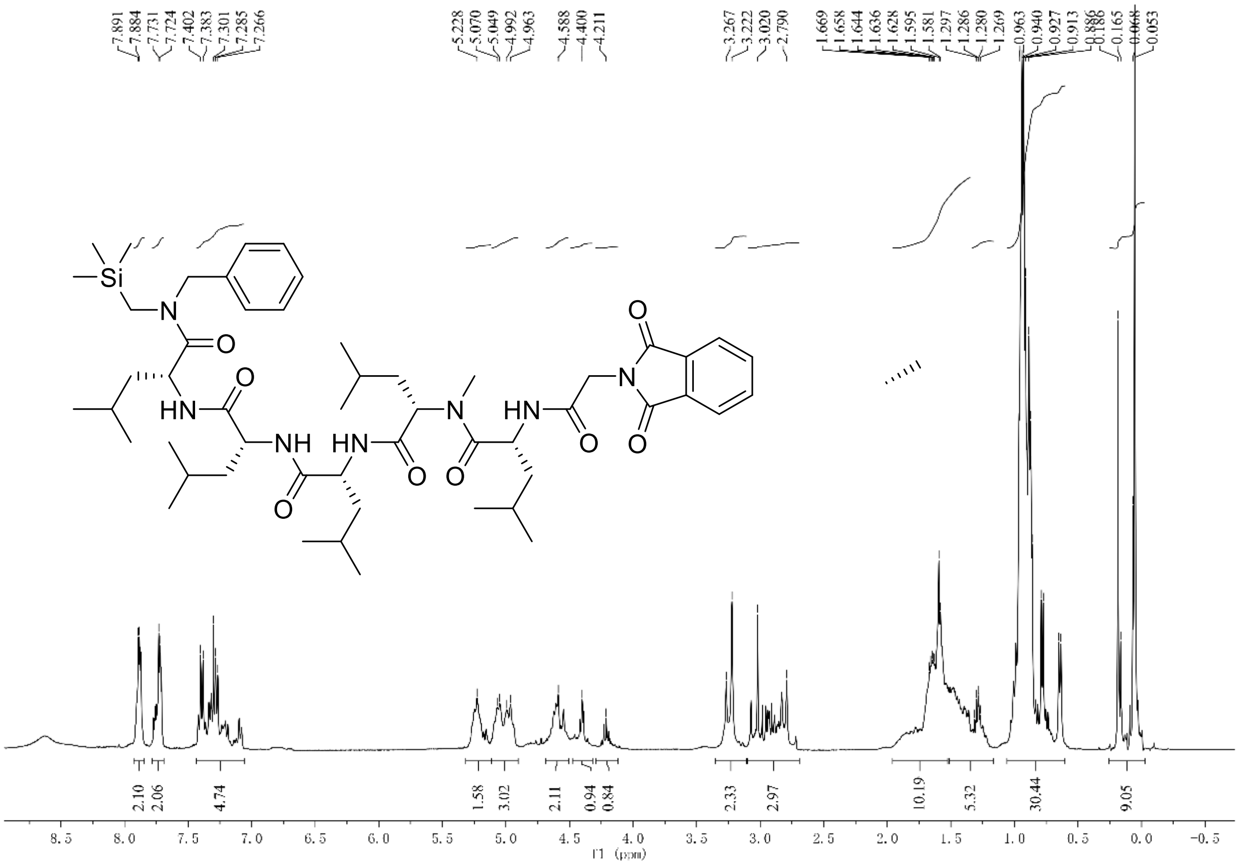

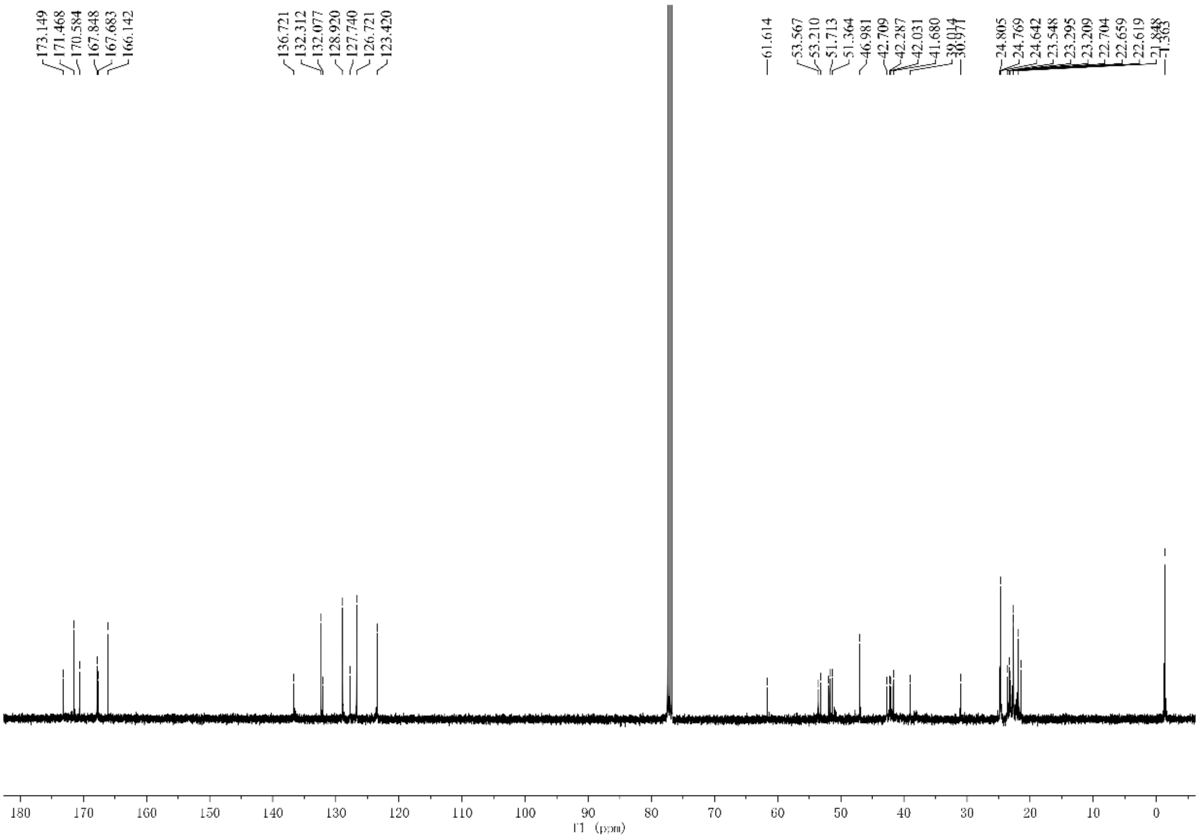
173.149
171.468
170.584
167.848
167.683
166.142
136.721
132.312
132.077
128.920
127.740
126.721
123.420
61.614
53.567
53.210
51.713
51.364
46.981
42.709
42.287
42.031
41.680
39.014
30.971
24.805
24.769
24.642
23.548
23.295
23.209
22.704
22.659
22.619
21.848
-1.363
180
170
160
150
140
130
120
110
100
90
80
70
60
50
40
30
20
10
0
f1 (ppm)

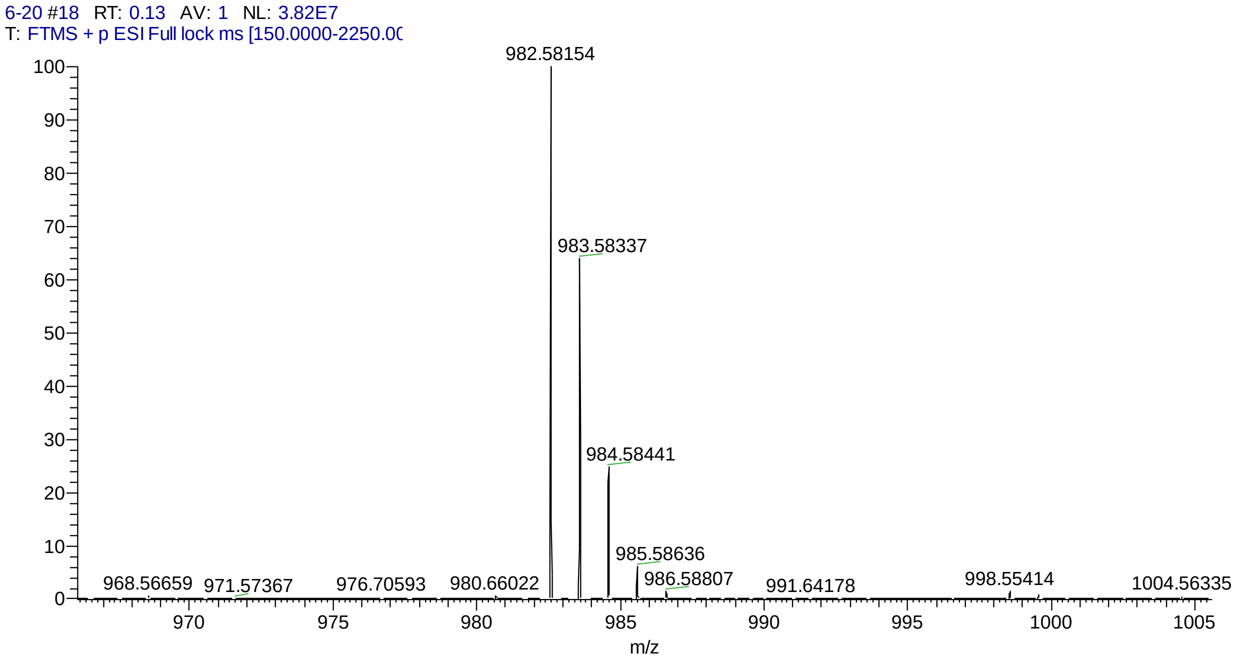
6-20 #18 RT: 0.13 AV: 1 NL: 3.82E7
T: FTMS + p ESI Full lock ms [150.0000-2250.00
982.58154
983.58337
984.58441
985.58636
986.58807
968.56659
971.57367
976.70593
980.66022
991.64178
998.55414
1004.56335
100
90
80
70
60
50
40
30
20
10
0
970
975
980
985
990
995
1000
1005
m/z

(6) ^{1}H, ^{13}C-NMR and HRMS of **14**.

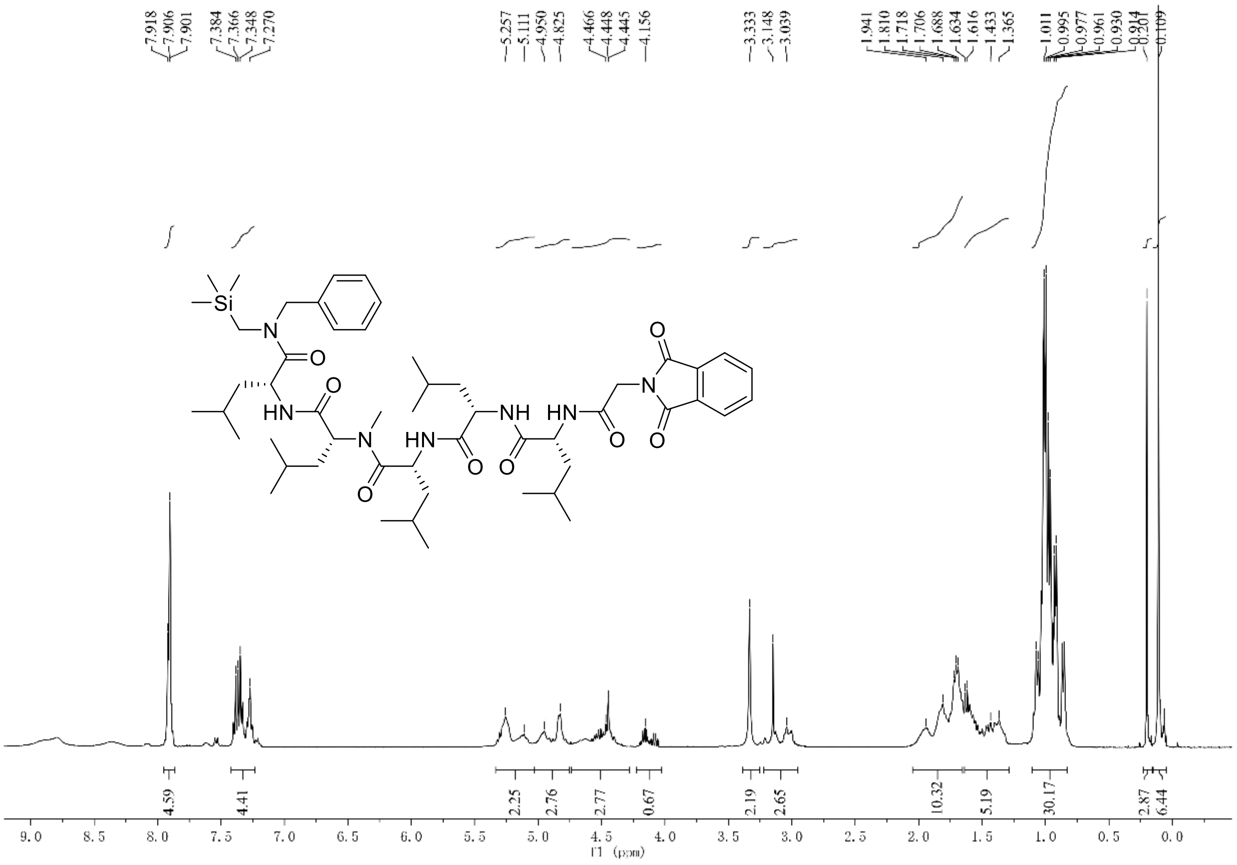

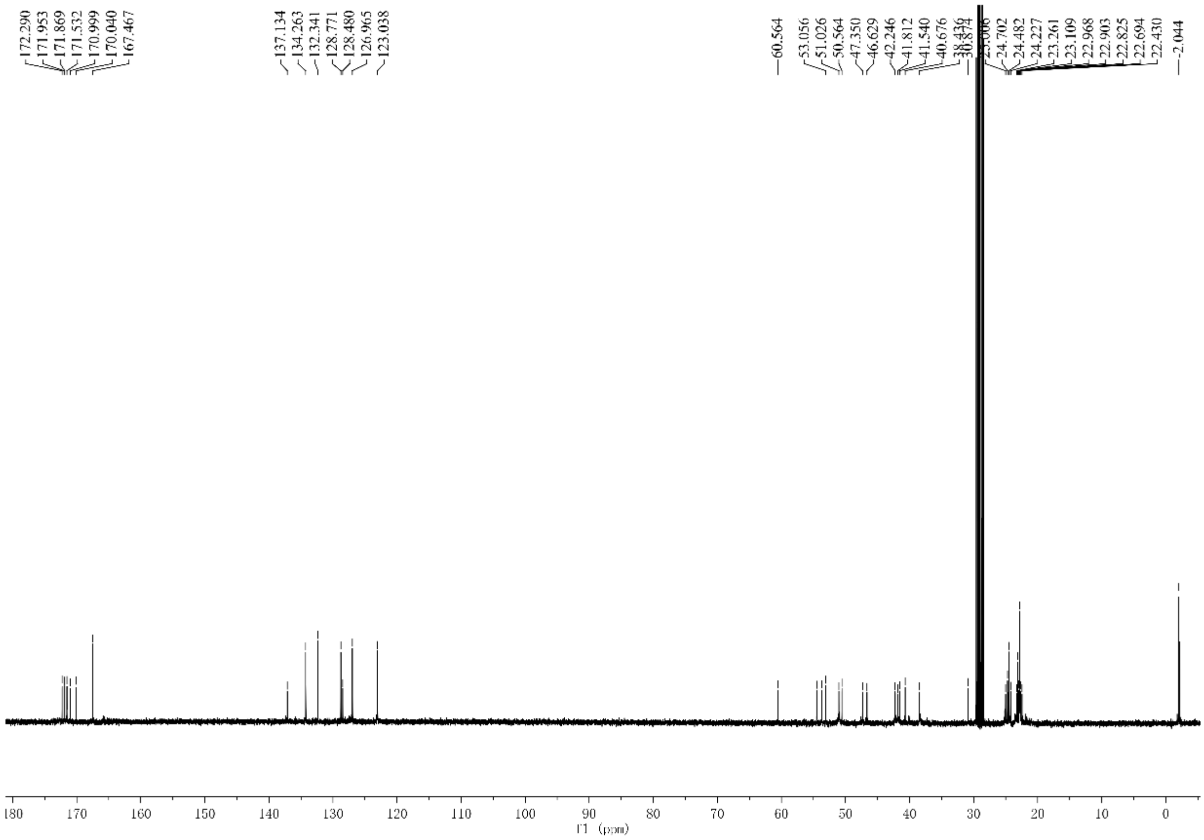

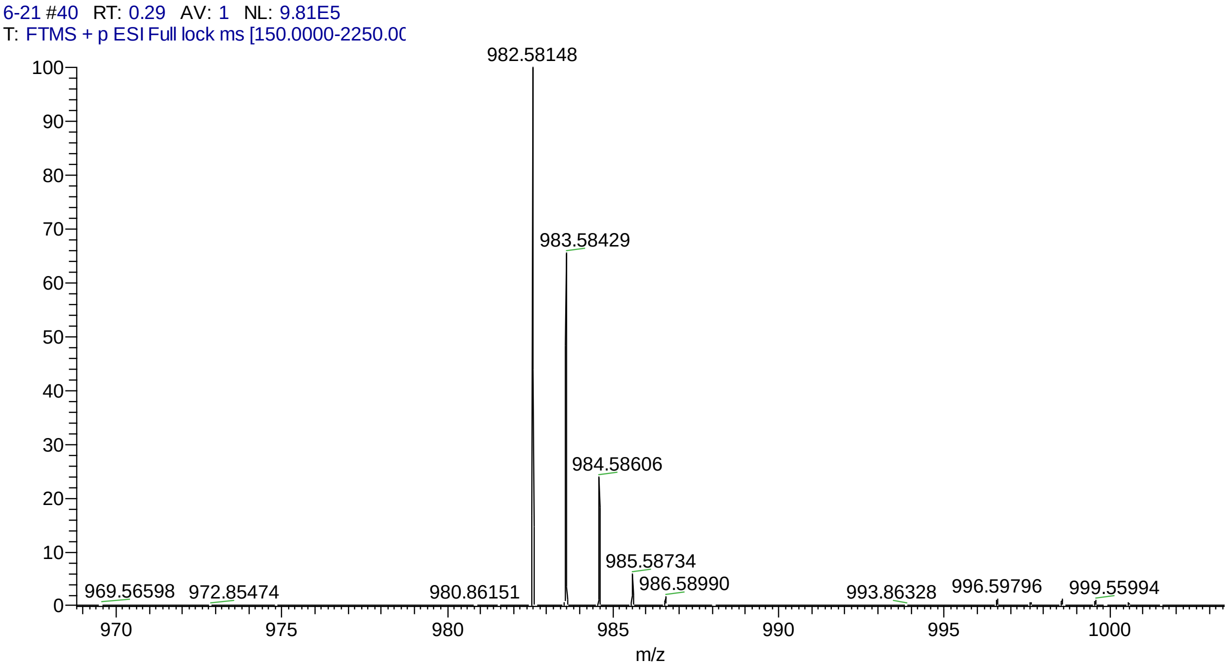

(7) ^{1}H, ^{13}C-NMR and HRMS of **15**.

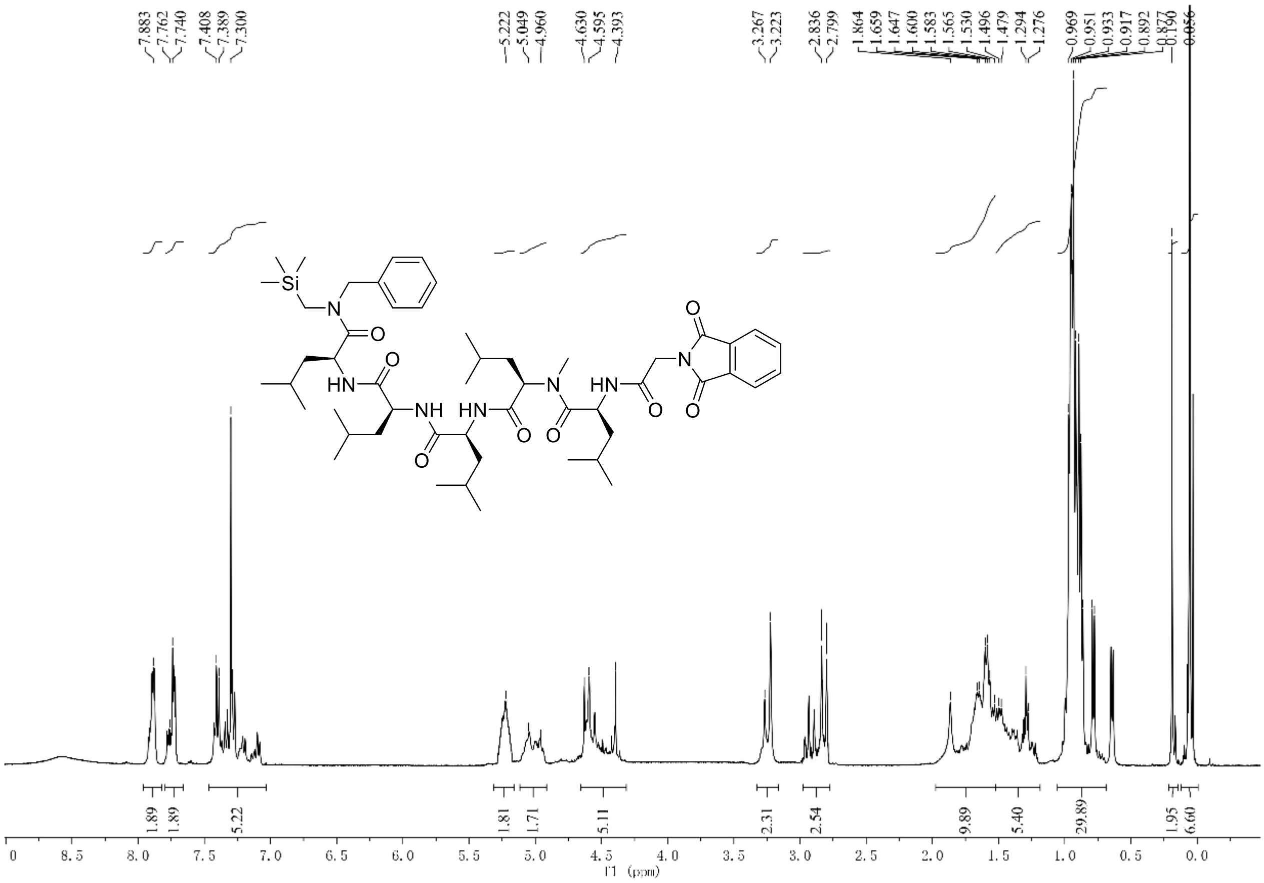

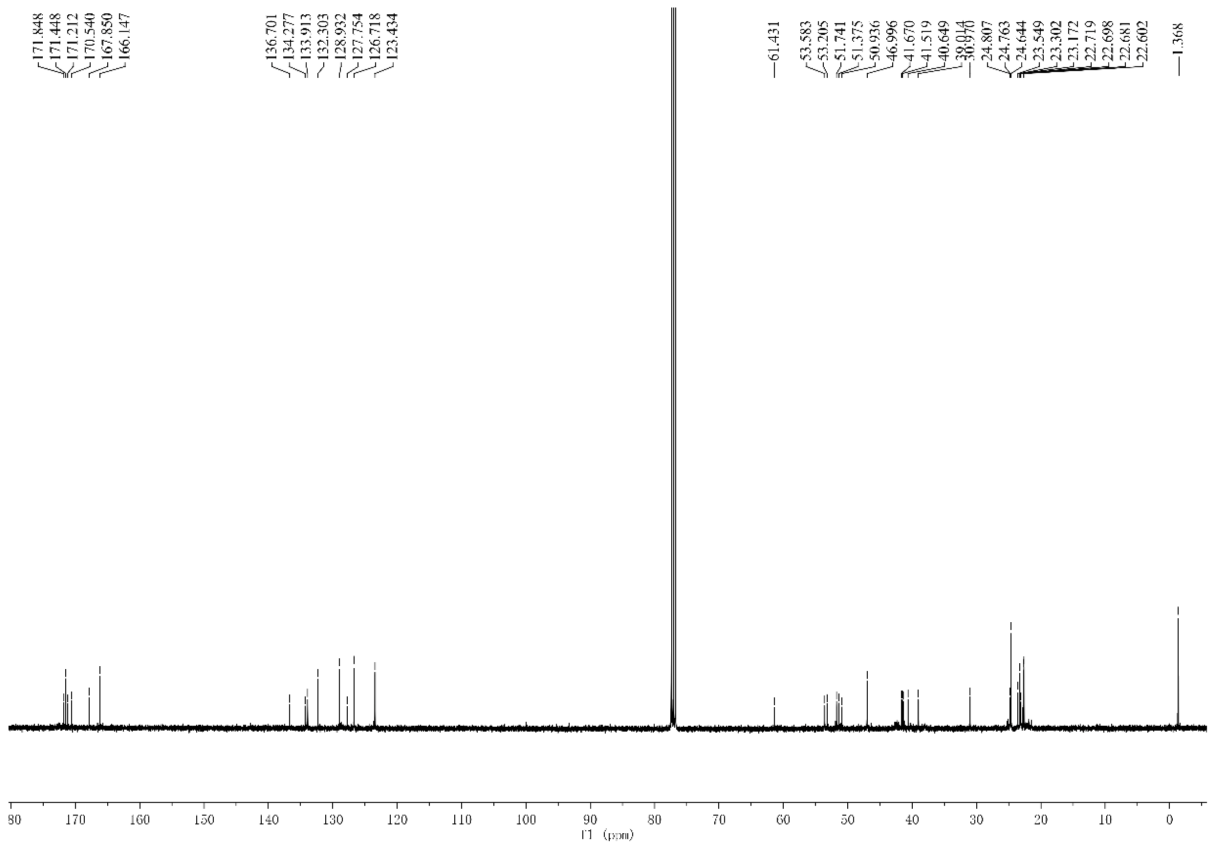
171.848
171.448
171.212
170.540
167.850
166.147
136.701
134.277
133.913
132.303
128.932
127.754
126.718
123.434
61.431
53.583
53.205
51.741
51.375
50.936
46.996
41.670
41.519
40.649
39.014
30.970
24.807
24.763
24.644
23.549
23.302
23.172
22.719
22.698
22.681
22.602
1.368
f1 (ppm)

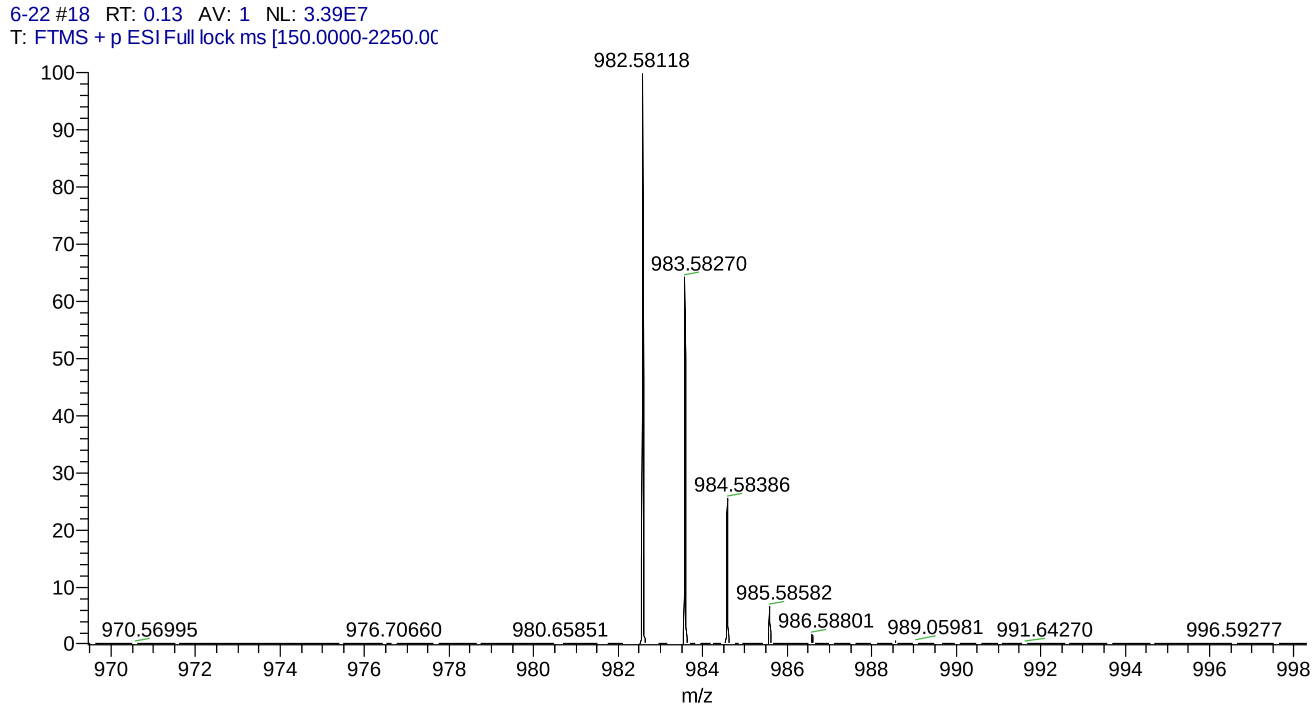
6-22 #18 RT: 0.13 AV: 1 NL: 3.39E7
T: FTMS + p ESI Full lock ms [150.0000-2250.00
982.58118
983.58270
984.58386
985.58582
986.58801
970.56995
976.70660
980.65851
989.05981
991.64270
996.59277
m/z

(8) ^{1}H, ^{13}C-NMR and HRMS of **16**.

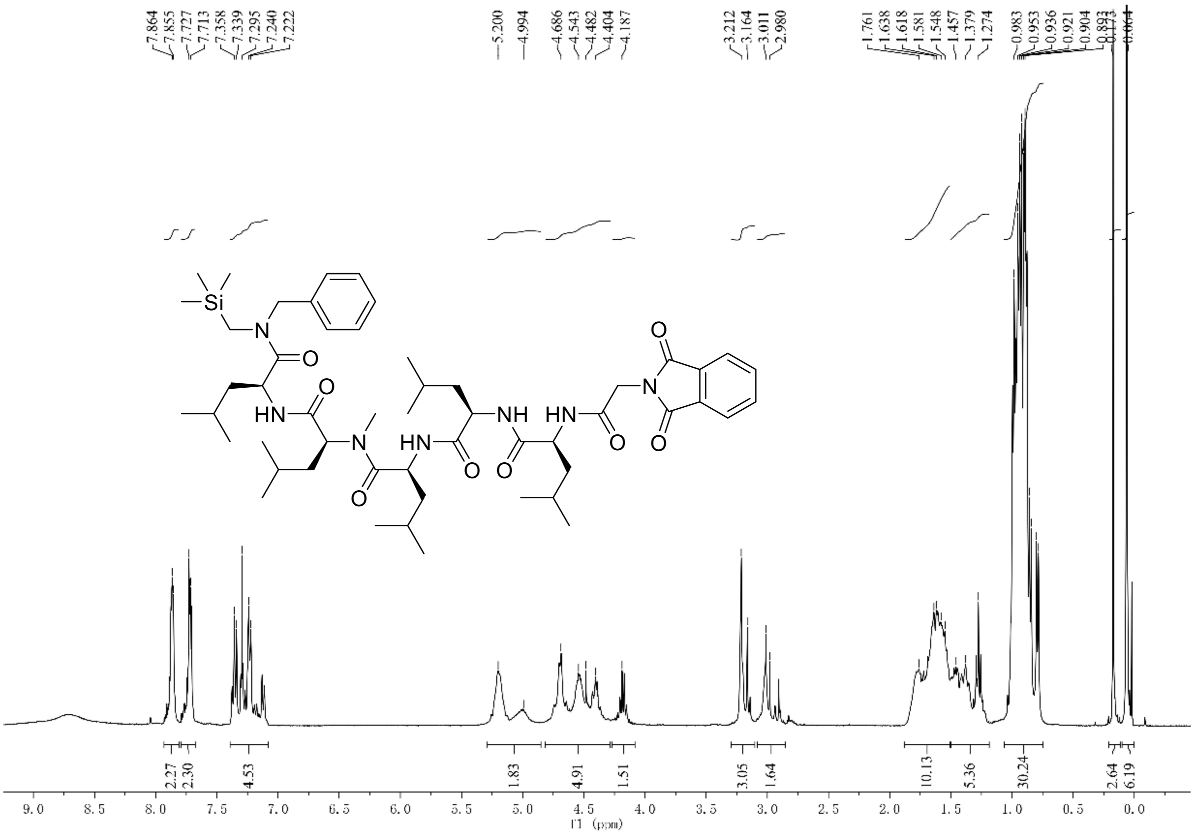

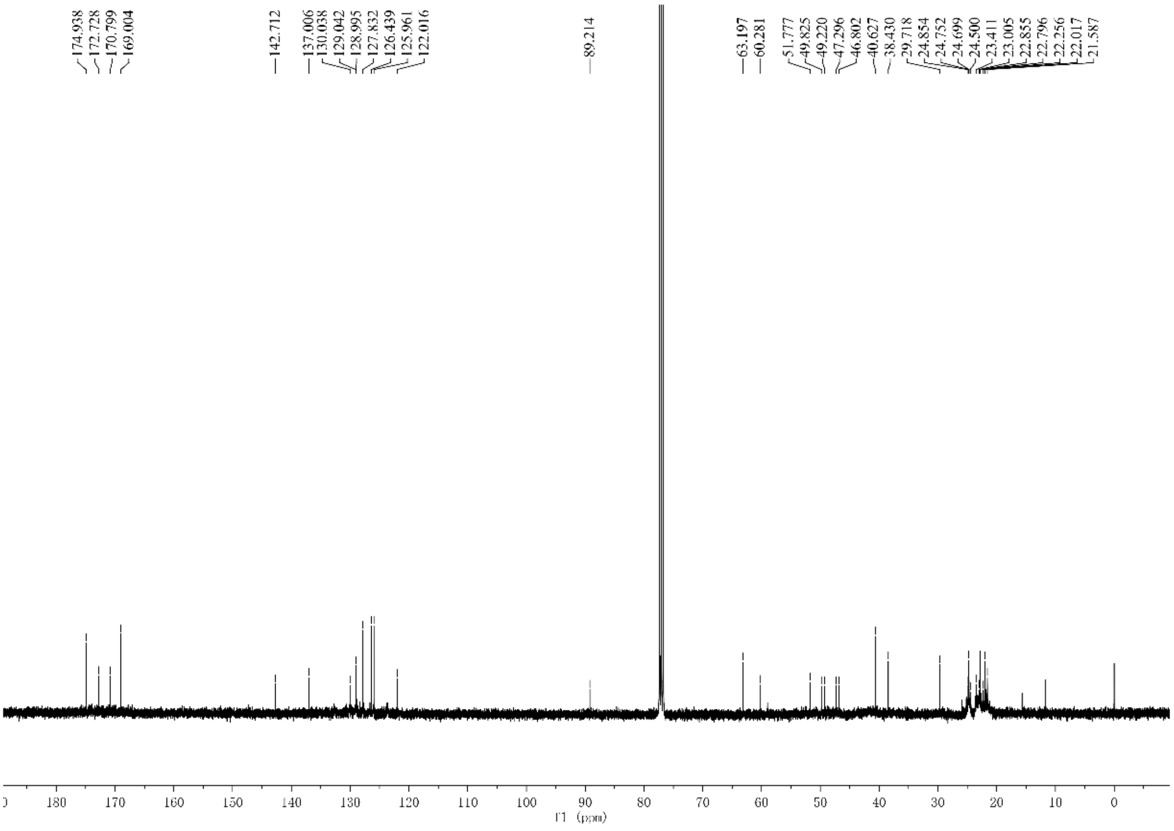

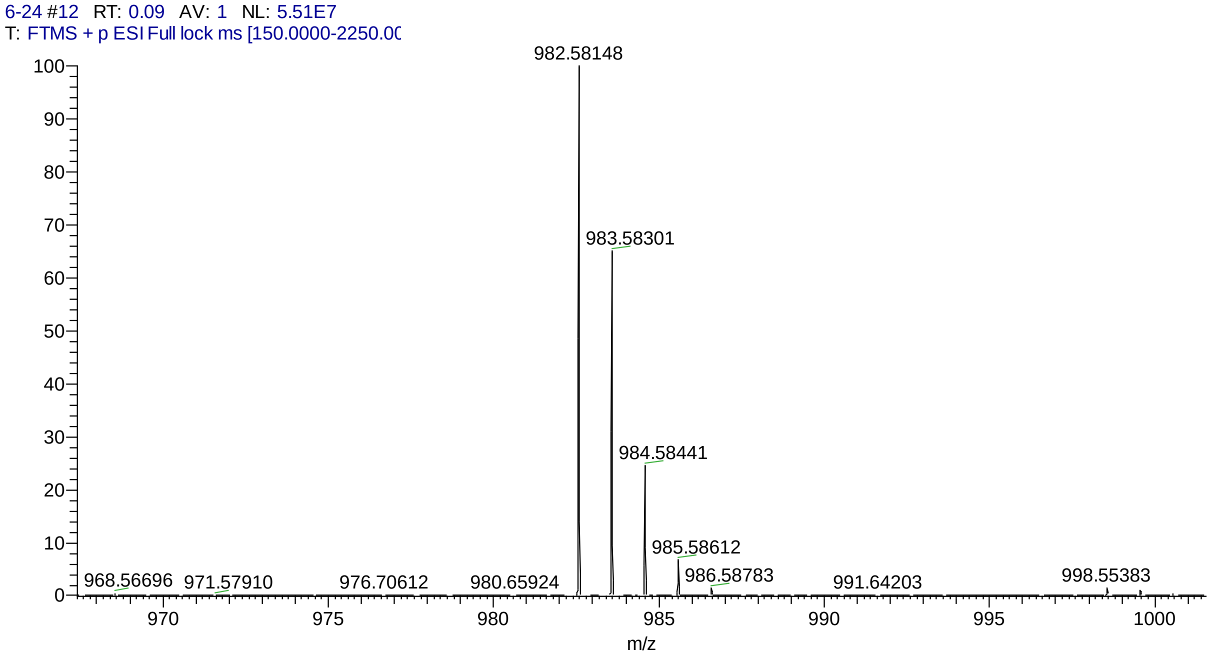

5. ^{1}H, ^{13}C-NMR, HRMS and HPLC of cyclic peptides.

(1) ^{1}H, ^{13}C-NMR, HRMS and HPLC of **1**.

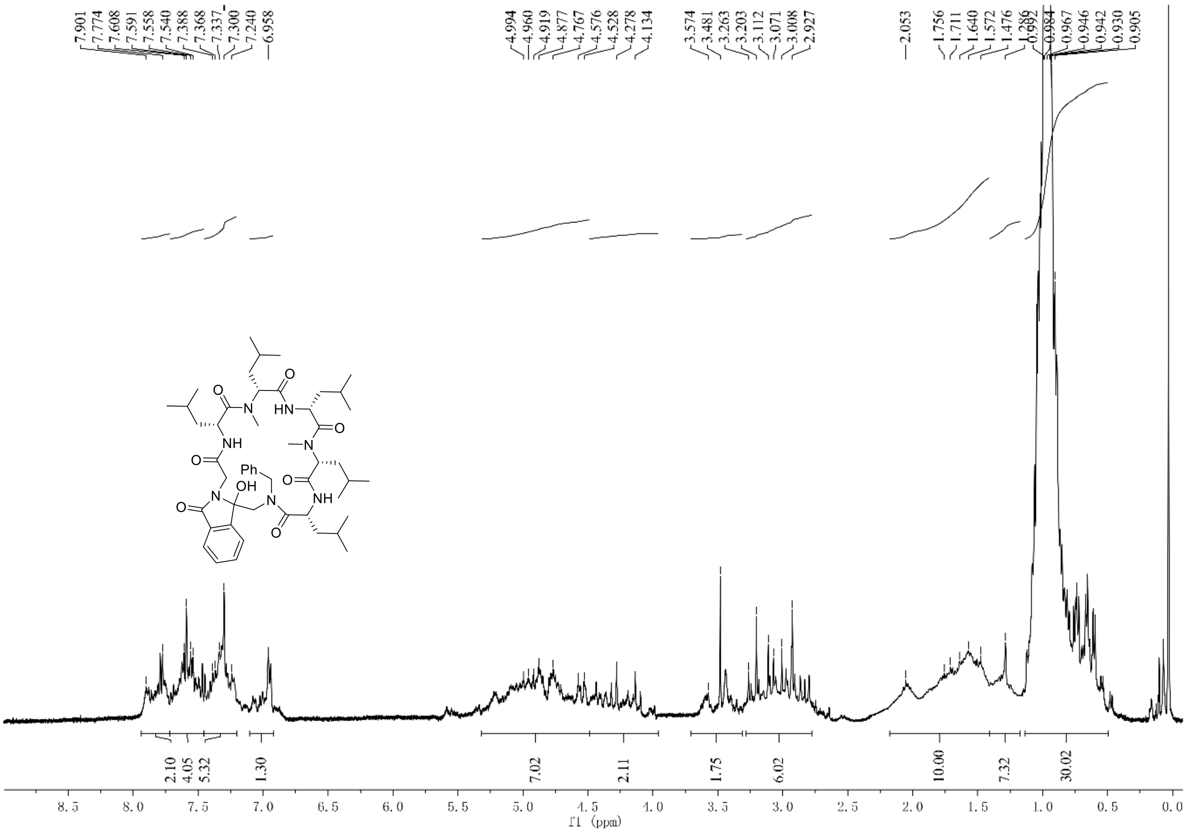

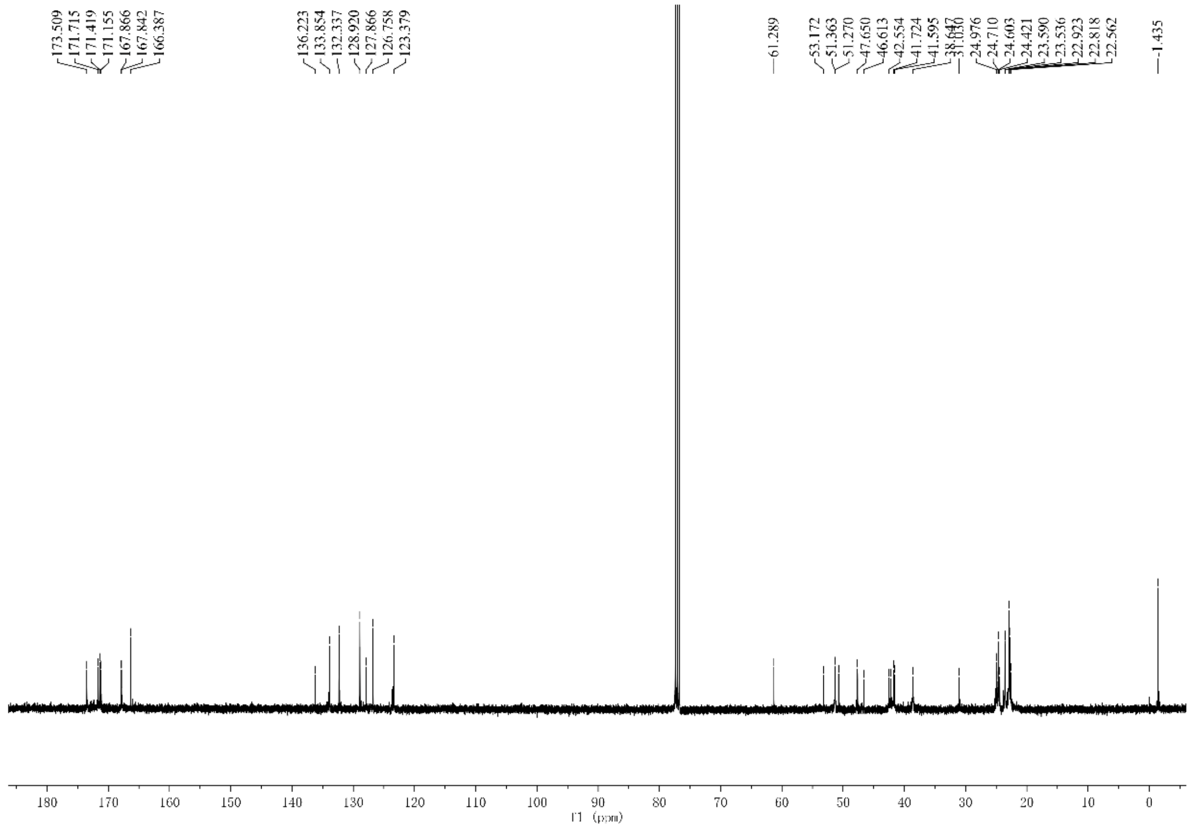
173.509
171.715
171.419
171.155
167.866
167.842
166.387
136.223
133.854
132.337
128.920
127.866
126.758
123.379
61.289
53.172
51.363
51.270
47.650
46.613
42.554
41.724
41.595
38.647
31.030
24.976
24.710
24.603
24.421
23.590
23.536
22.923
22.818
22.562
-1.435
f1 (ppm)

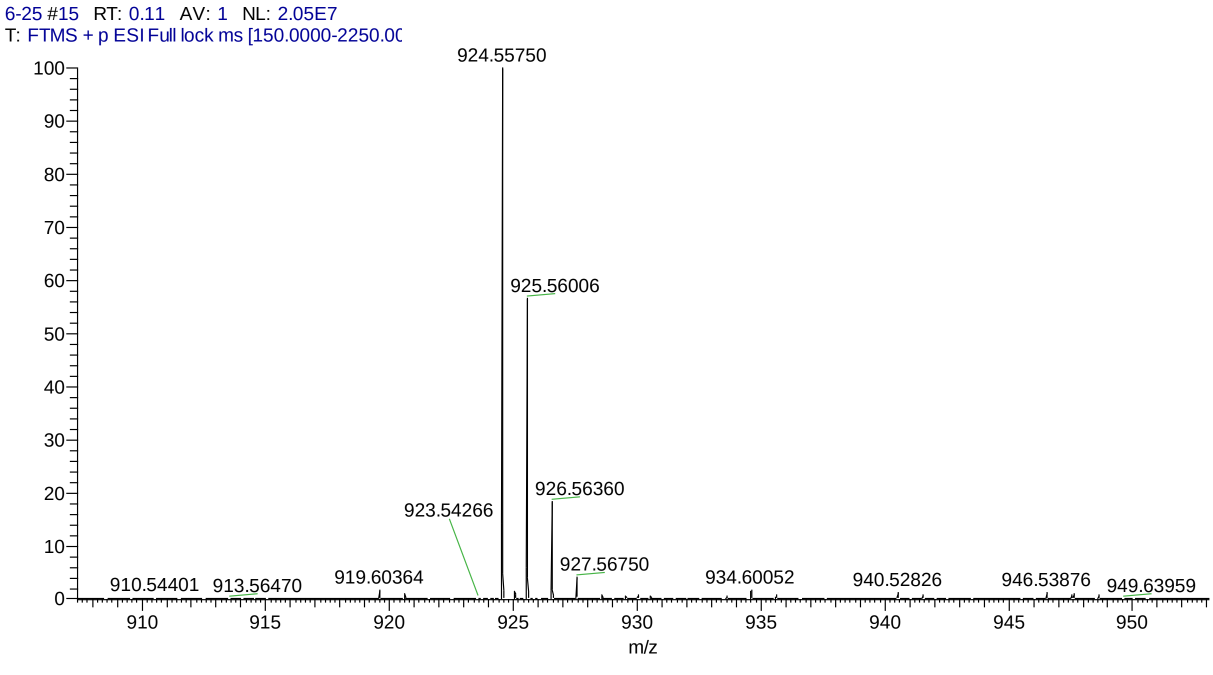
6-25 #15 RT: 0.11 AV: 1 NL: 2.05E7
T: FTMS + p ESI Full lock ms [150.0000-2250.00
924.55750
925.56006
926.56360
923.54266
927.56750
910.54401
913.56470
919.60364
934.60052
940.52826
946.53876
949.63959
m/z

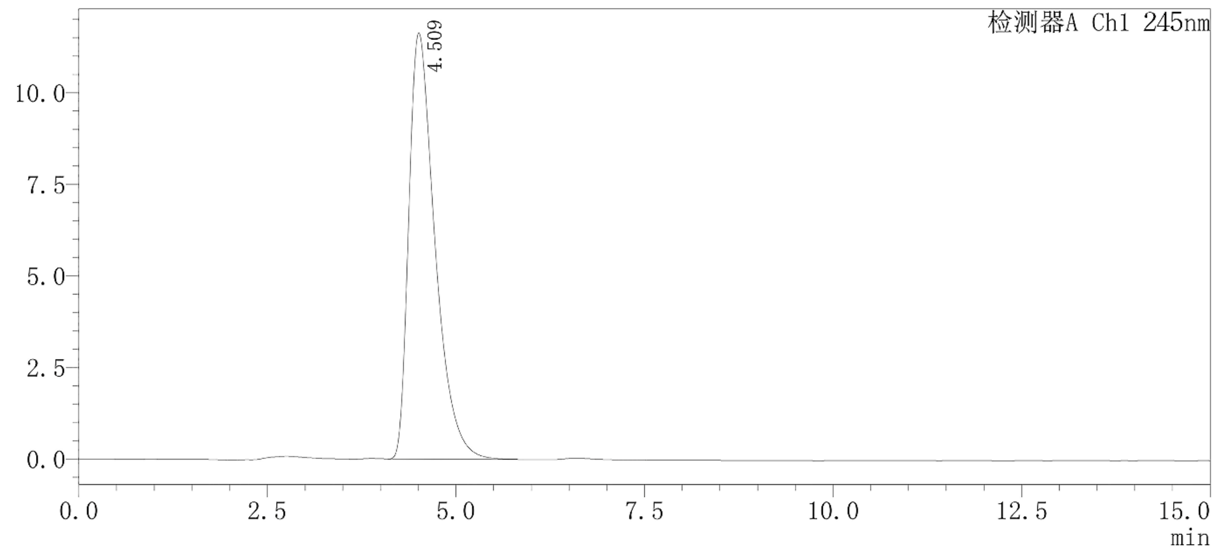

HPLC conditions: Shiseido Capcell PAK C18 (150×4.0 mm, 5 μm) was used as the column at 30 ˚C, and the mobile phase flow rate was 1 mL/min. During the analytical run, the elution was carried out using mobile phases A (Ultrapure water) and B (acetonitrile), the percentage of mobile phases B was 30%, while the detection wavelength was 245 nm.

(2) ^{1}H, ^{13}C-NMR, HRMS and HPLC of **2**.

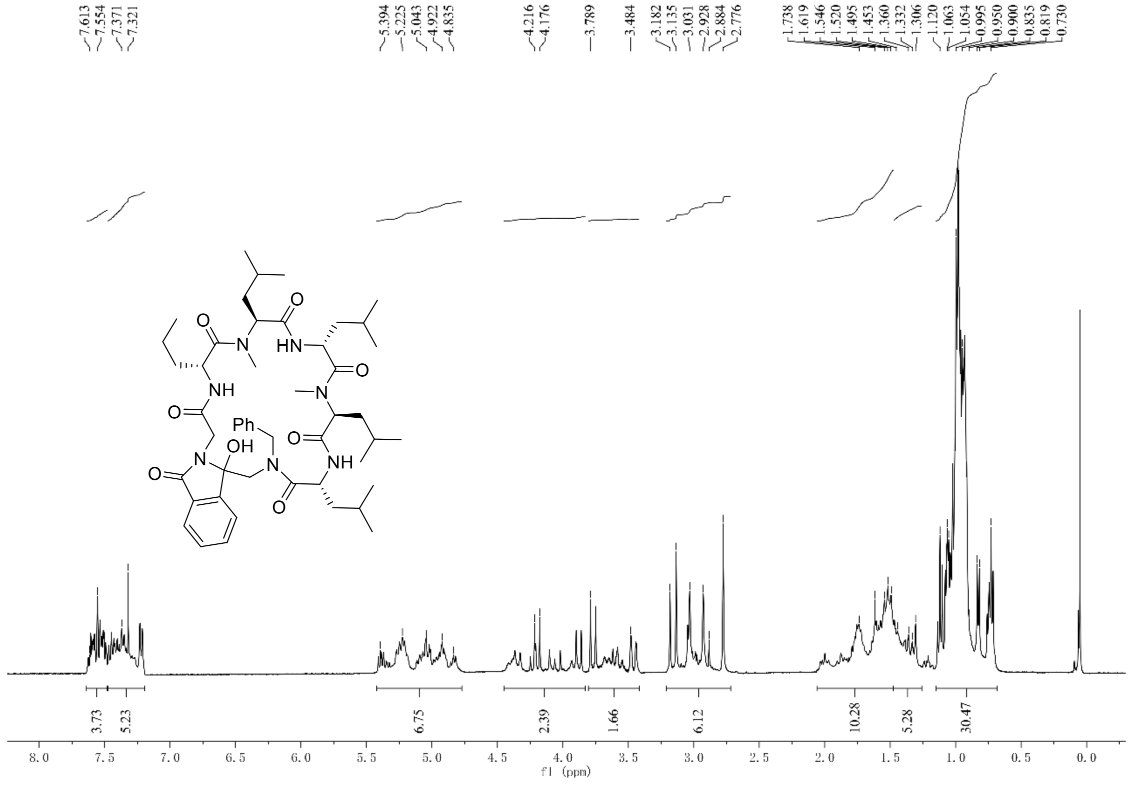

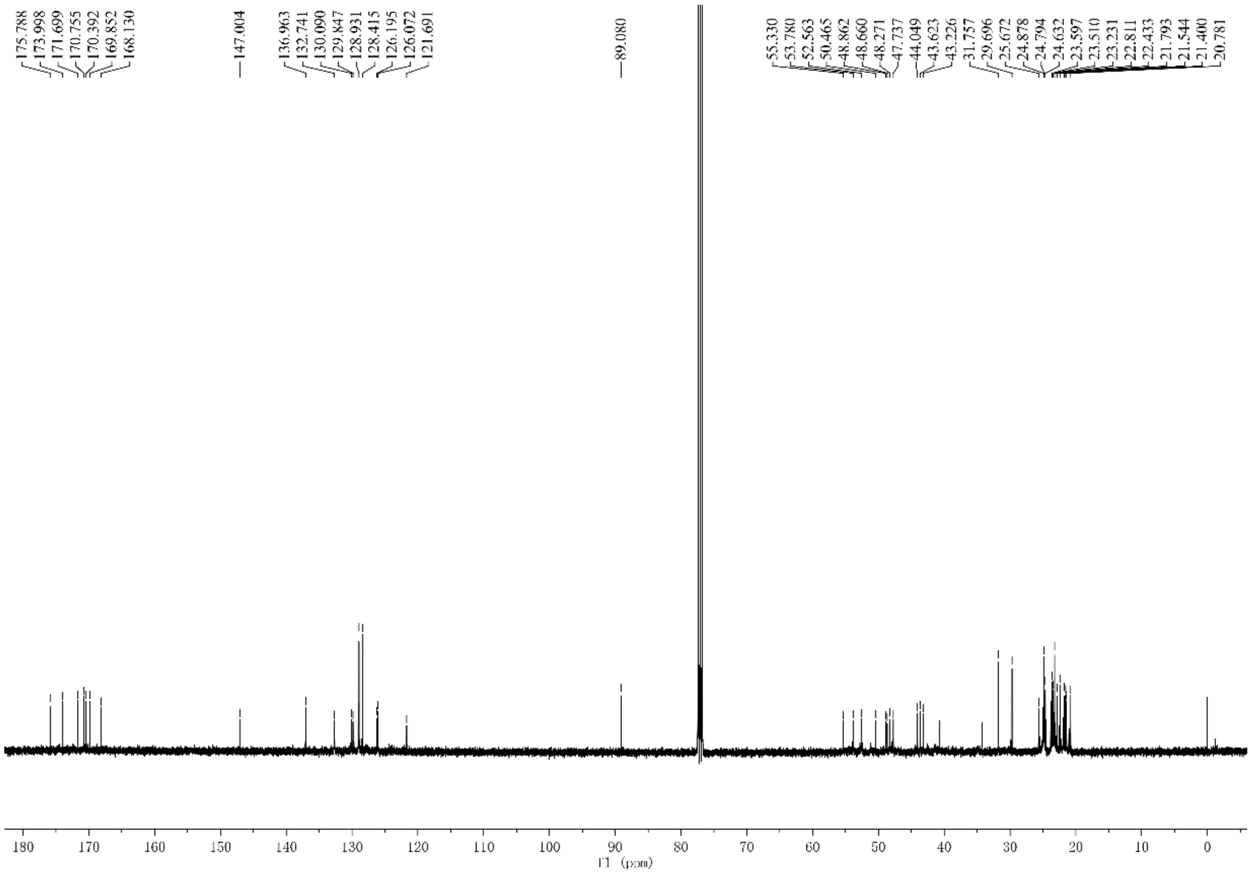
175.788
173.998
171.699
170.755
170.392
169.852
168.130
147.004
136.963
132.741
130.090
129.847
128.931
128.415
126.195
126.072
121.691
89.080
55.330
53.780
52.563
50.465
48.862
48.660
48.271
47.737
44.049
43.623
43.226
31.757
29.696
25.672
24.878
24.794
24.632
23.597
23.510
23.231
22.811
22.433
21.793
21.544
21.400
20.781
f1 (ppm)

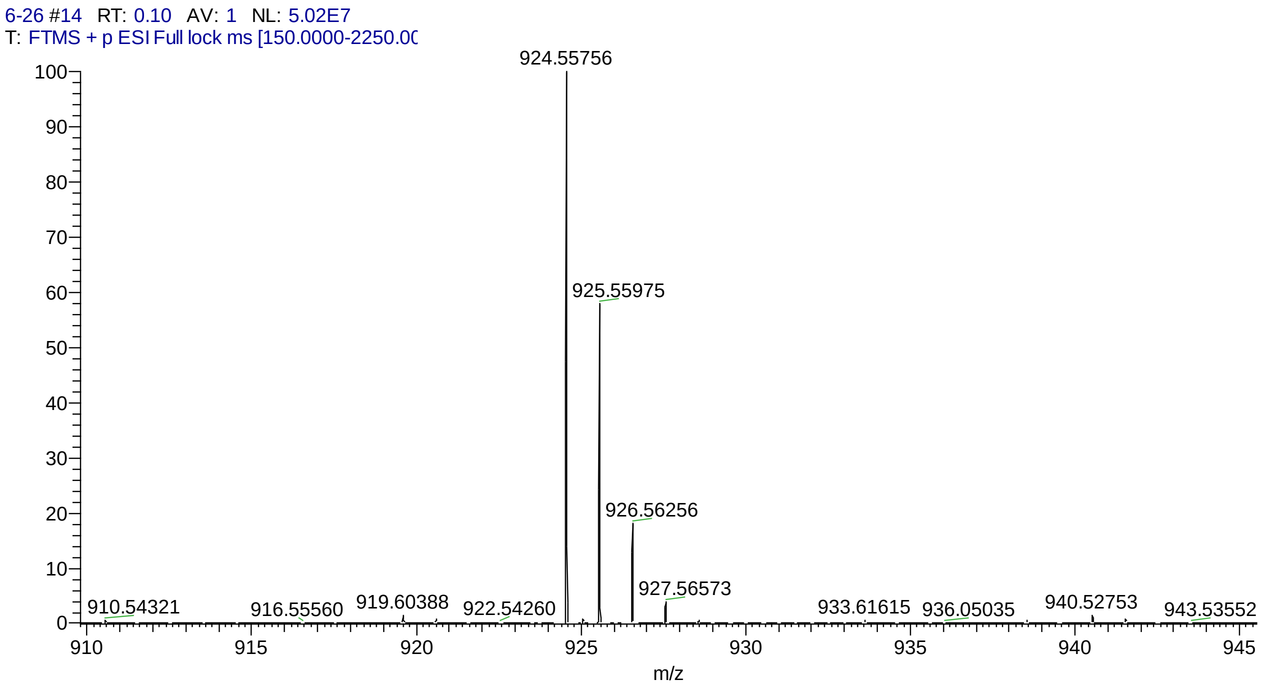
6-26 #14 RT: 0.10 AV: 1 NL: 5.02E7
T: FTMS + p ESI Full lock ms [150.0000-2250.00
924.55756
925.55975
926.56256
927.56573
910.54321
916.55560
919.60388
922.54260
933.61615
936.05035
940.52753
943.53552
m/z

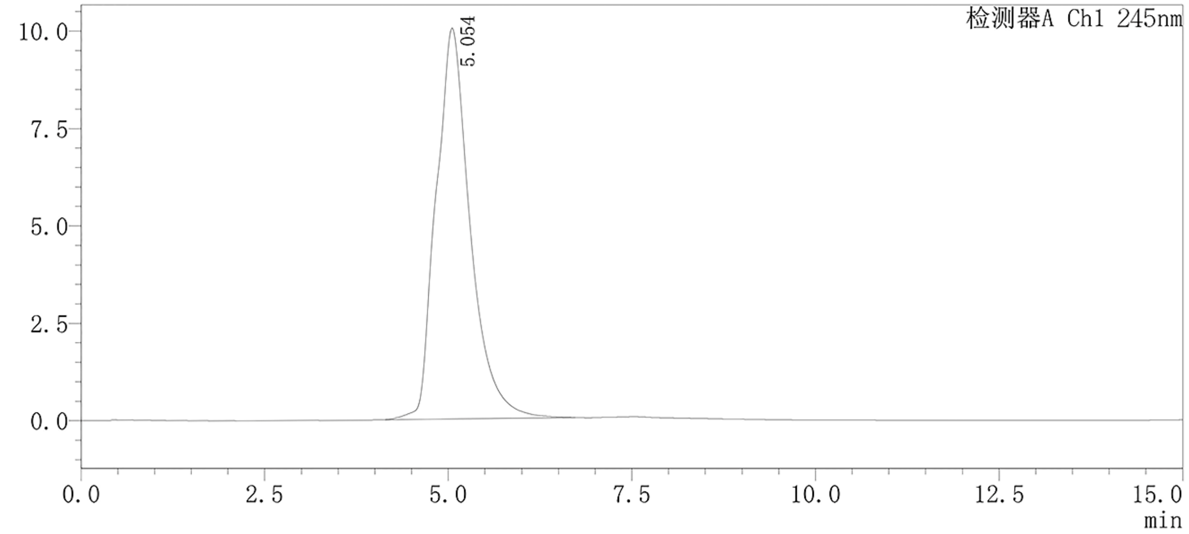

HPLC conditions: Shiseido Capcell PAK C18 (150×4.0 mm, 5 μm) was used as the column at 30 ˚C, and the mobile phase flow rate was 1 mL/min. During the analytical run, the elution was carried out using mobile phases A (Ultrapure water) and B (acetonitrile), the percentage of mobile phases B was 30%, while the detection wavelength was 245 nm.

(3) ^{1}H, ^{13}C-NMR, HRMS and HPLC of **3**.

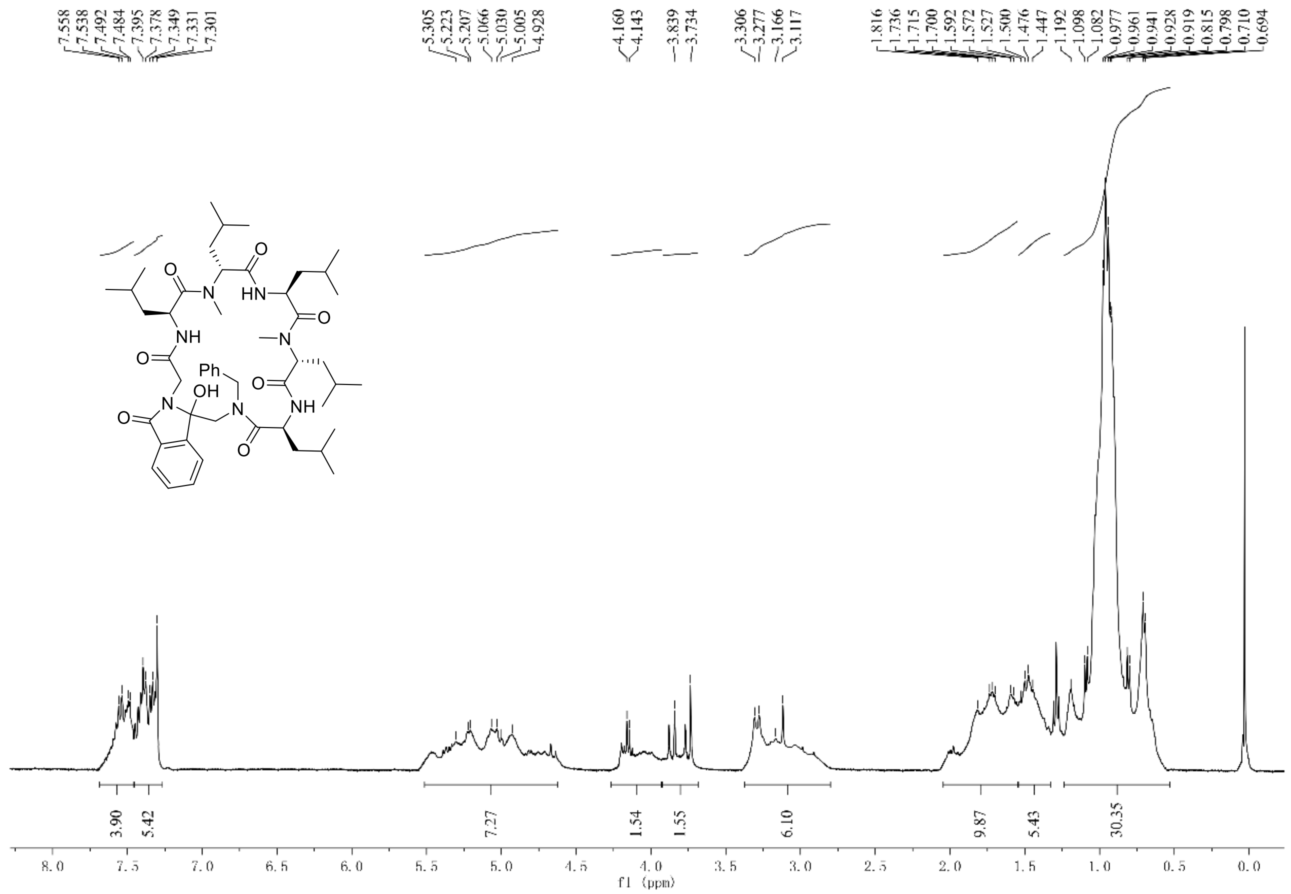

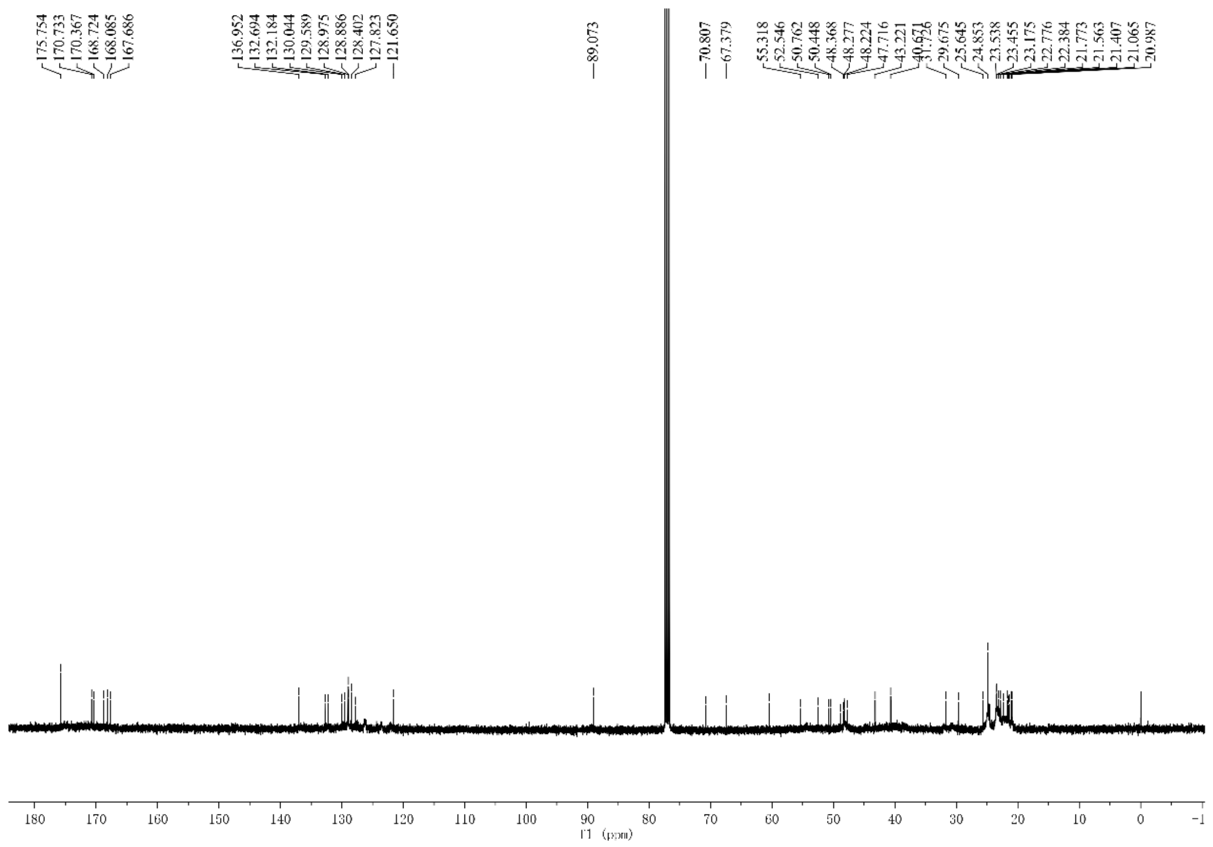
175.754
170.733
170.367
168.724
168.085
167.686
136.952
132.694
132.184
130.044
129.589
128.975
128.886
128.402
127.823
121.650
89.073
70.807
67.379
55.318
52.546
50.762
50.448
48.368
48.277
48.224
47.716
43.221
40.671
31.726
29.675
25.645
24.853
23.538
23.455
23.175
22.776
22.384
21.773
21.563
21.407
21.065
20.987
f1 (ppm)

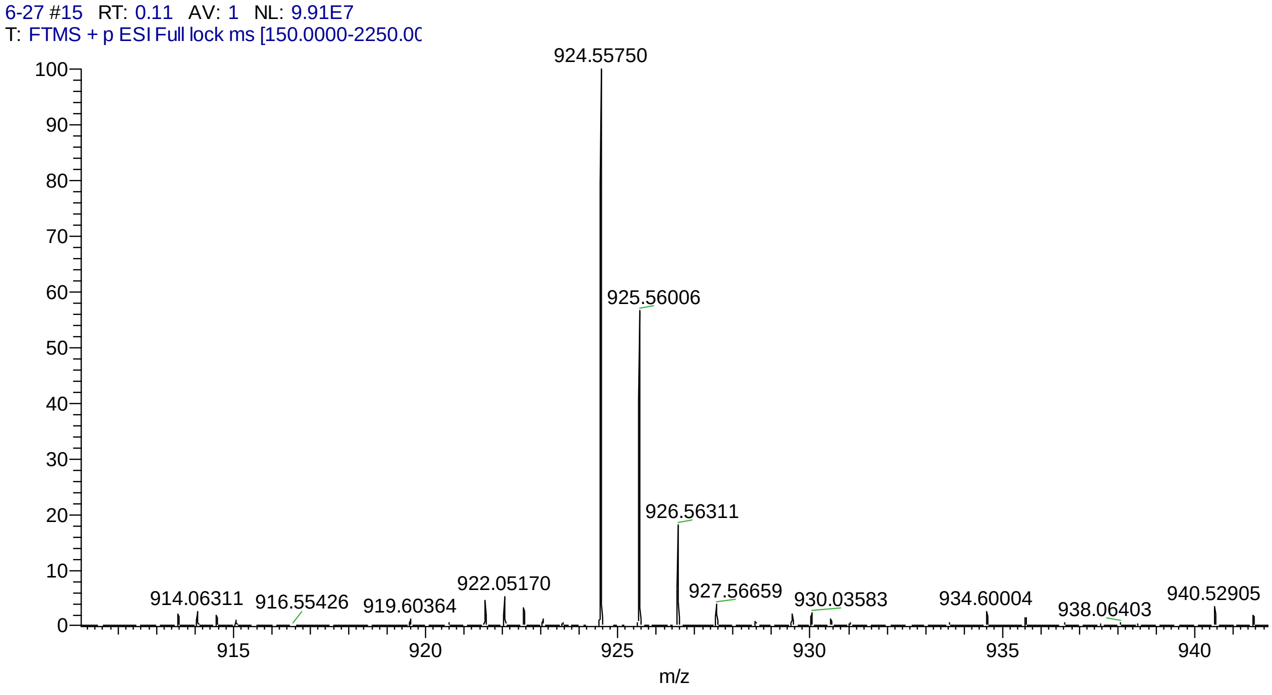
6-27 #15 RT: 0.11 AV: 1 NL: 9.91E7
T: FTMS + p ESI Full lock ms [150.0000-2250.0(
924.55750
925.56006
926.56311
927.56659
914.06311
916.55426
919.60364
922.05170
930.03583
934.60004
938.06403
940.52905
m/z

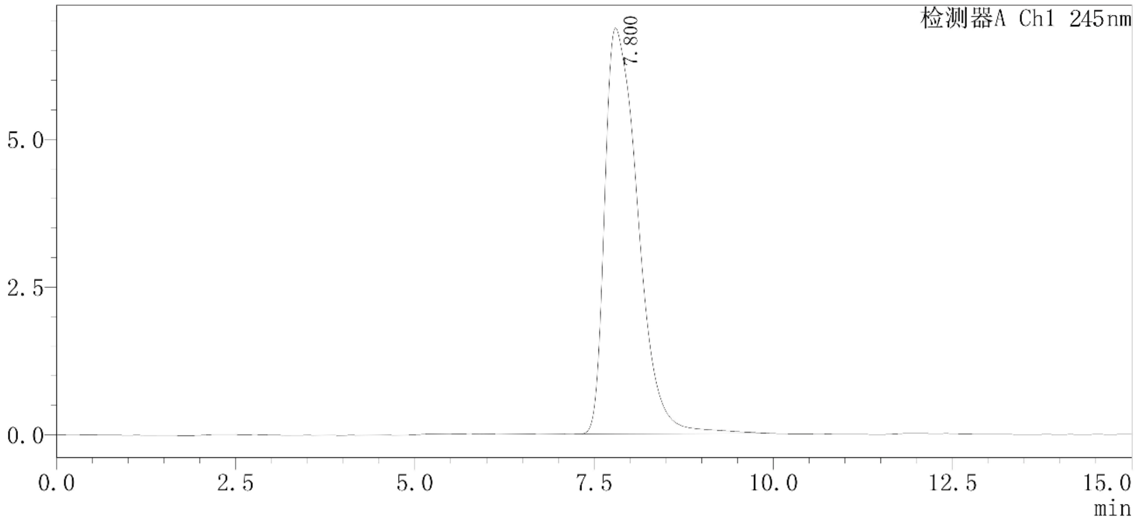

HPLC conditions: Shiseido Capcell PAK C18 (150×4.0 mm, 5 μm) was used as the column at 30 ˚C, and the mobile phase flow rate was 1 mL/min. During the analytical run, the elution was carried out using mobile phases A (Ultrapure water) and B (acetonitrile), the percentage of mobile phases B was 30%, while the detection wavelength was 245 nm.

(4) ^{1}H, ^{13}C-NMR, HRMS and HPLC of **4**.

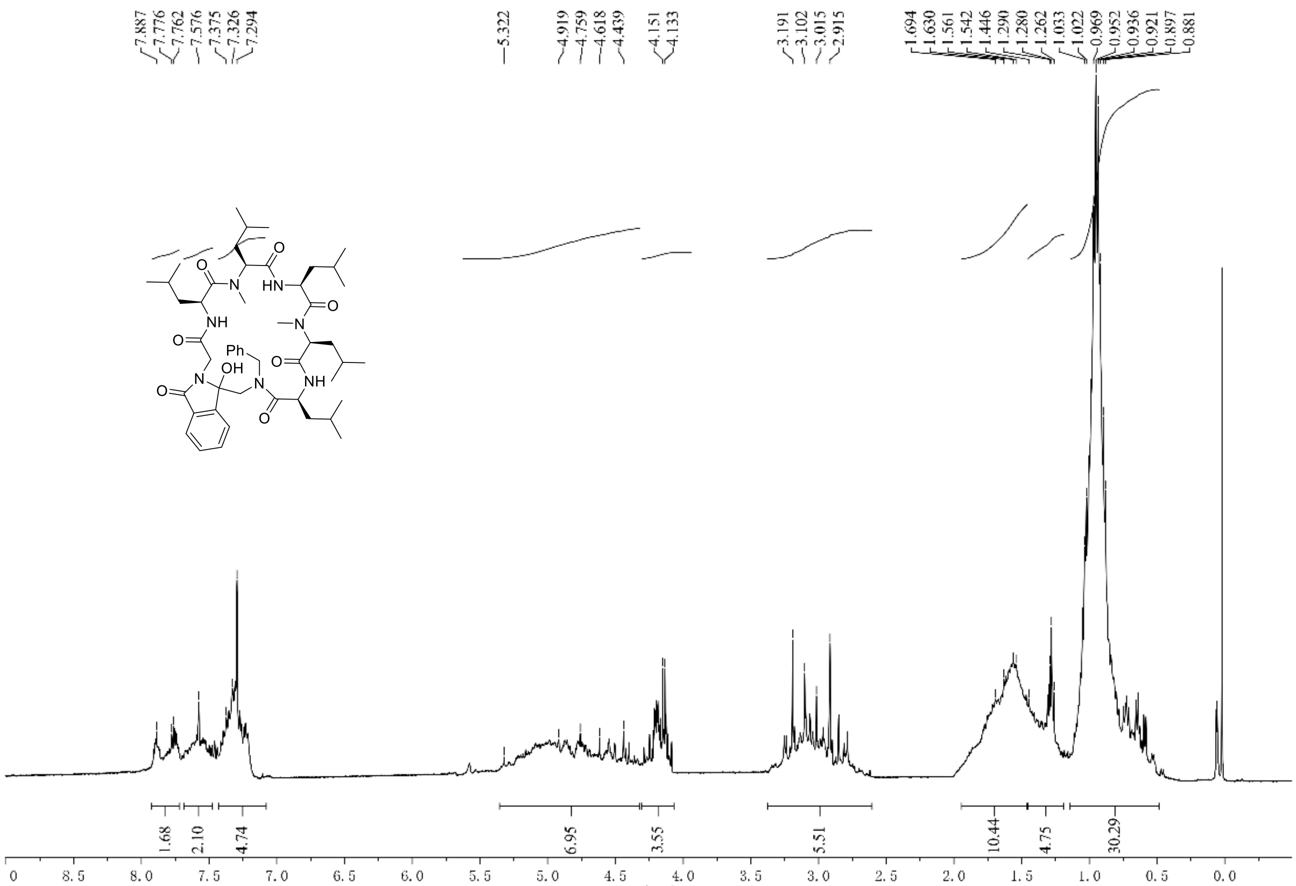

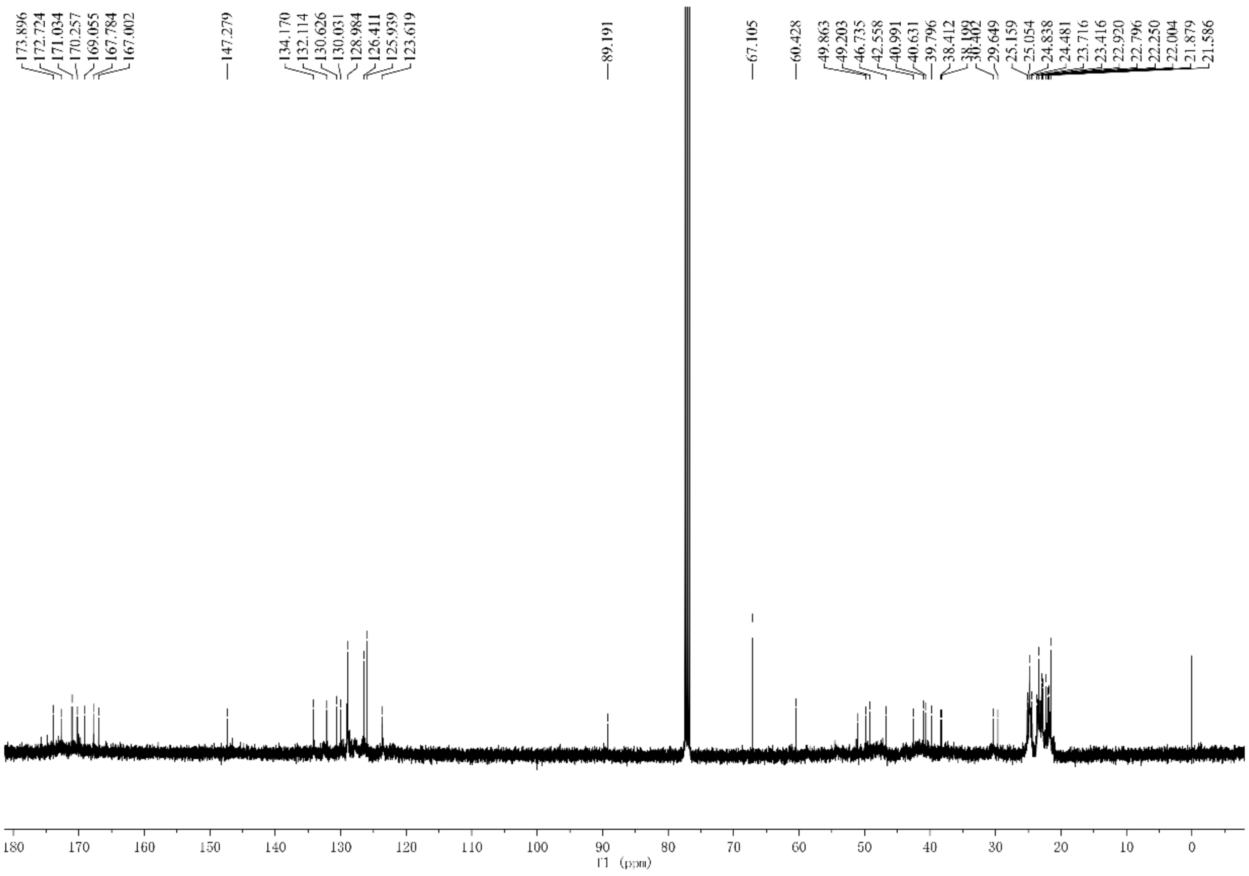
173.896
172.724
171.034
170.257
169.055
167.784
167.002
147.279
134.170
132.114
130.626
130.031
128.984
126.411
125.939
123.619
89.191
67.105
60.428
49.863
49.203
46.735
42.558
40.991
40.631
39.796
38.412
30.402
29.649
25.159
25.054
24.838
24.481
23.716
23.416
22.920
22.796
22.250
22.004
21.879
21.586
f1 (ppm)

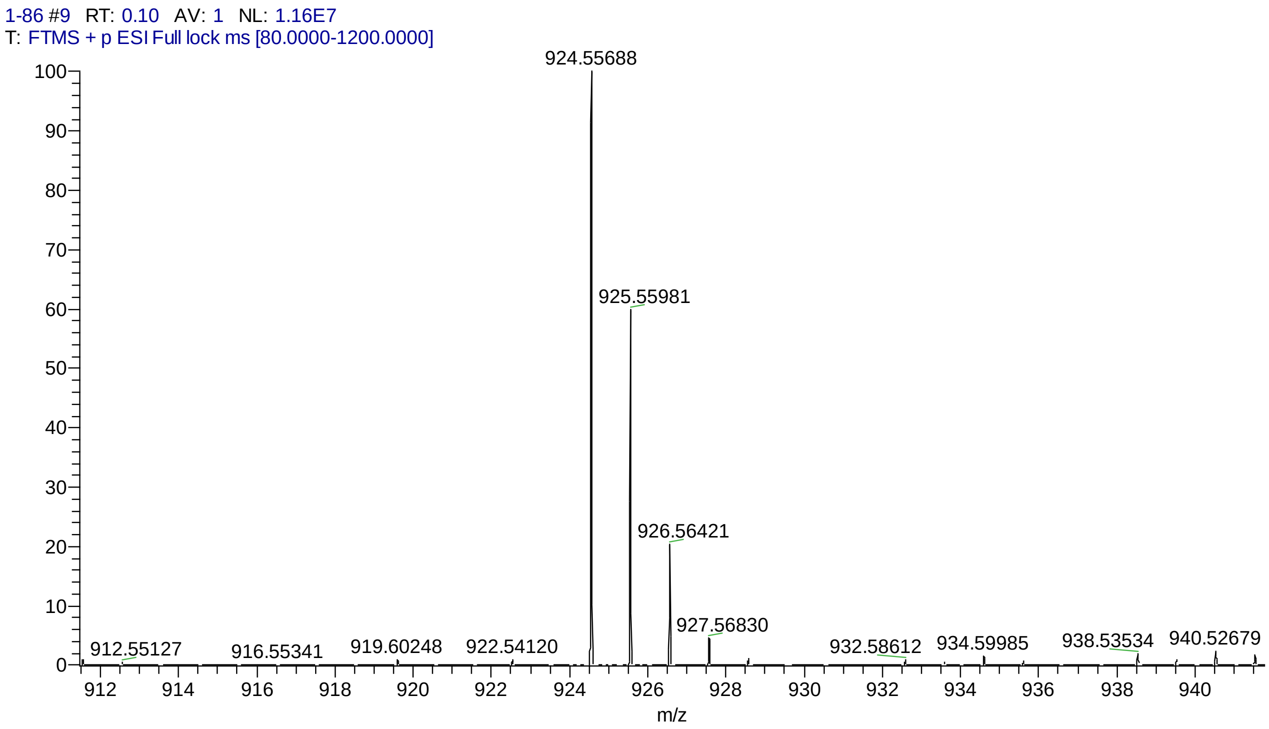
1-86 #9 RT: 0.10 AV: 1 NL: 1.16E7
T: FTMS + p ESI Full lock ms [80.0000-1200.0000]
924.55688
925.55981
926.56421
927.56830
912.55127
916.55341
919.60248
922.54120
932.58612
934.59985
938.53534
940.52679
m/z

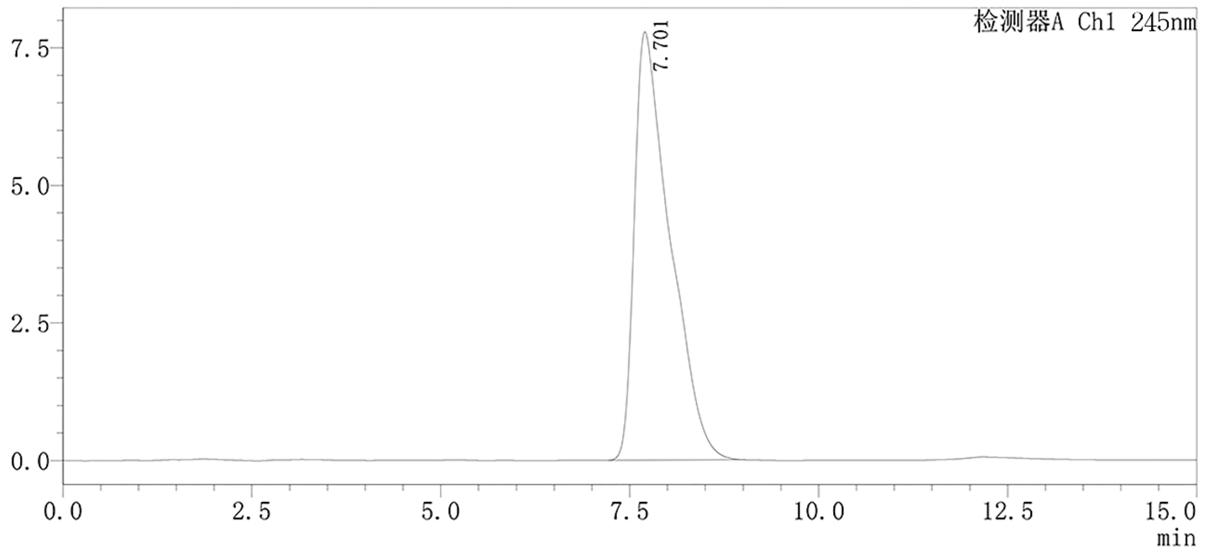

HPLC conditions: Shiseido Capcell PAK C18 (150×4.0 mm, 5 μm) was used as the column at 30 ˚C, and the mobile phase flow rate was 1 mL/min. During the analytical run, the elution was carried out using mobile phases A (Ultrapure water) and B (acetonitrile), the percentage of mobile phases B was 30%, while the detection wavelength was 245 nm.

(5) ^{1}H, ^{13}C-NMR, HRMS and HPLC of **5**.

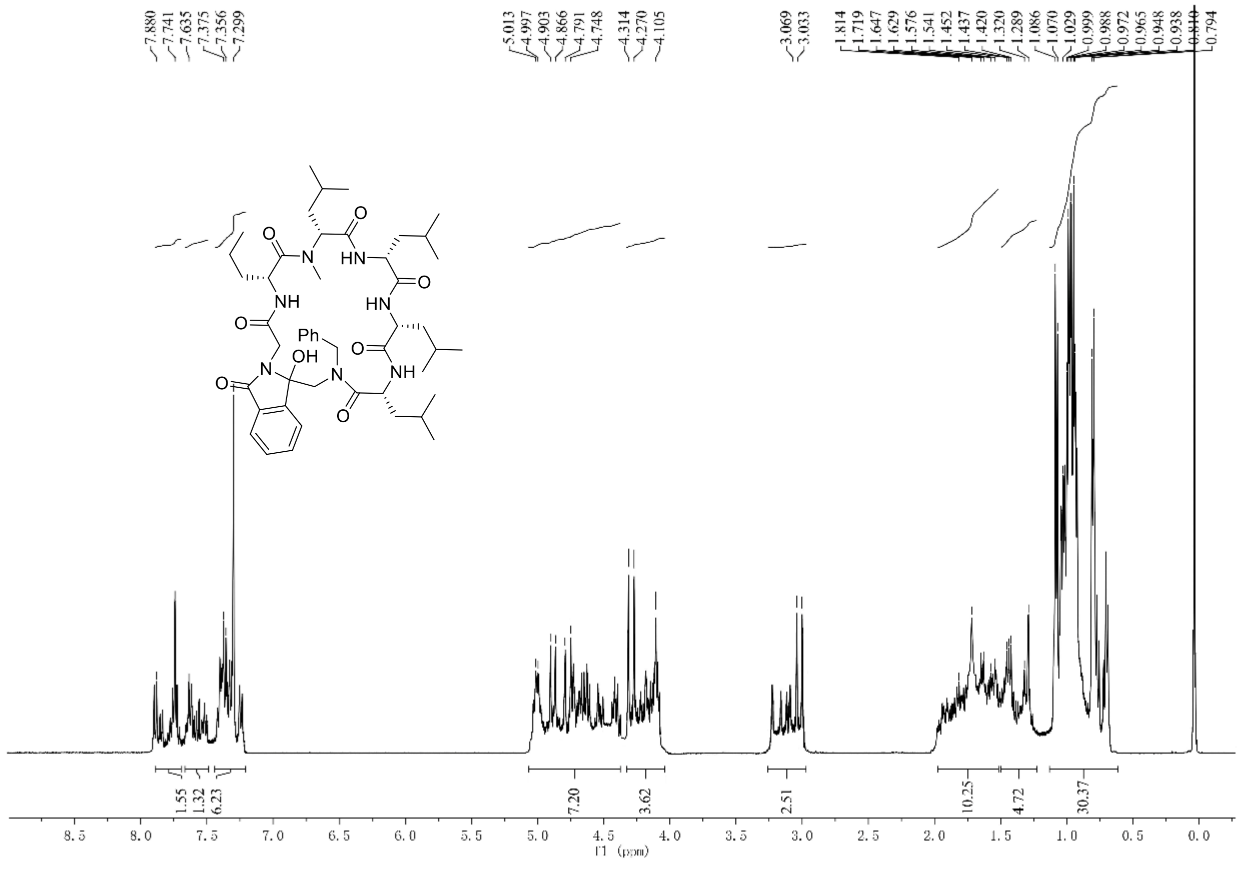

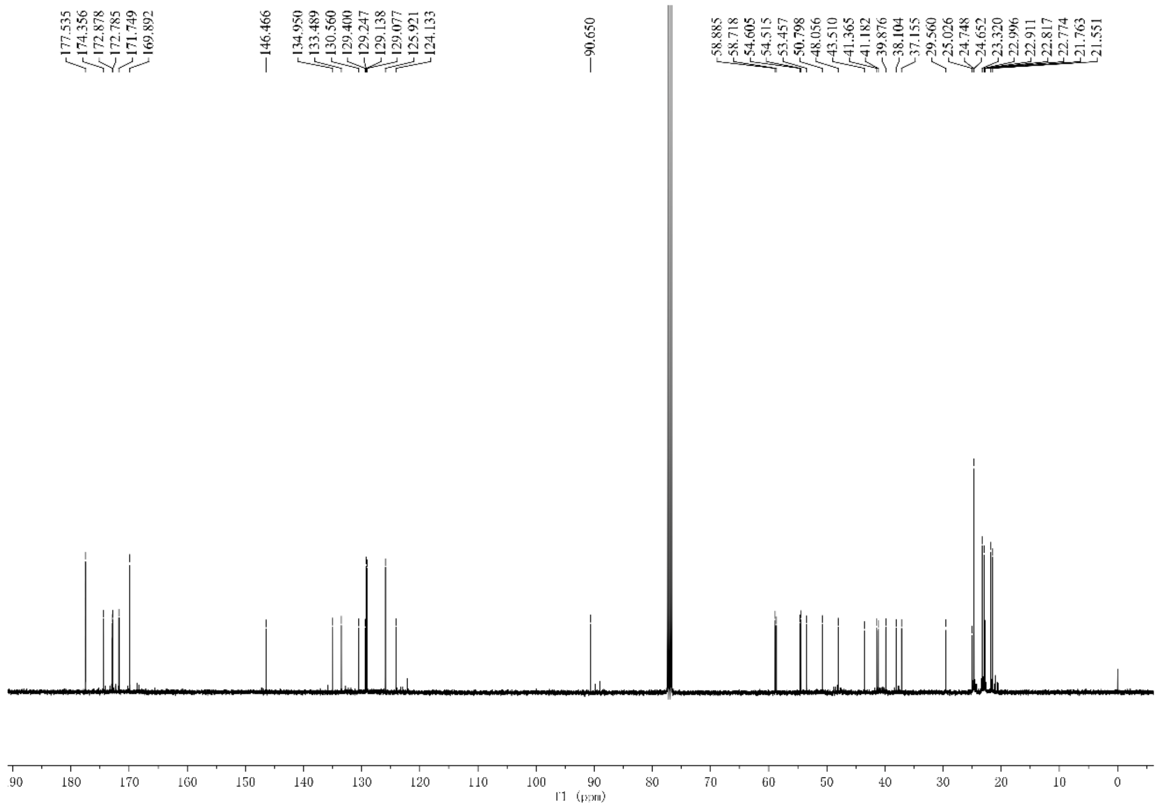
177.535
174.356
172.878
172.785
171.749
169.892
146.466
134.950
133.489
130.560
129.400
129.247
129.138
129.077
125.921
124.133
90.650
58.885
58.718
54.605
54.515
53.457
50.798
48.056
43.510
41.365
41.182
39.876
38.104
37.155
29.560
25.026
24.748
24.652
23.320
22.996
22.911
22.817
22.774
21.763
21.551
f1 (ppm)

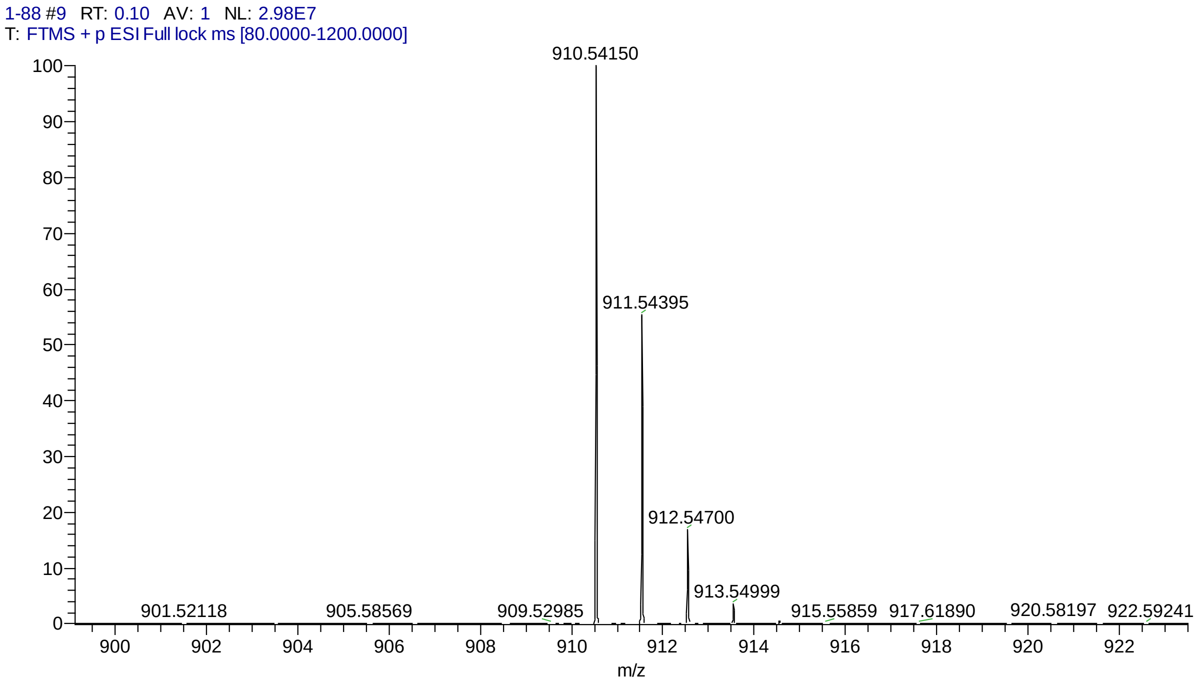
1-88 #9 RT: 0.10 AV: 1 NL: 2.98E7
T: FTMS + p ESI Full lock ms [80.0000-1200.0000]
910.54150
911.54395
912.54700
913.54999
901.52118
905.58569
909.52985
915.55859
917.61890
920.58197
922.59241
m/z

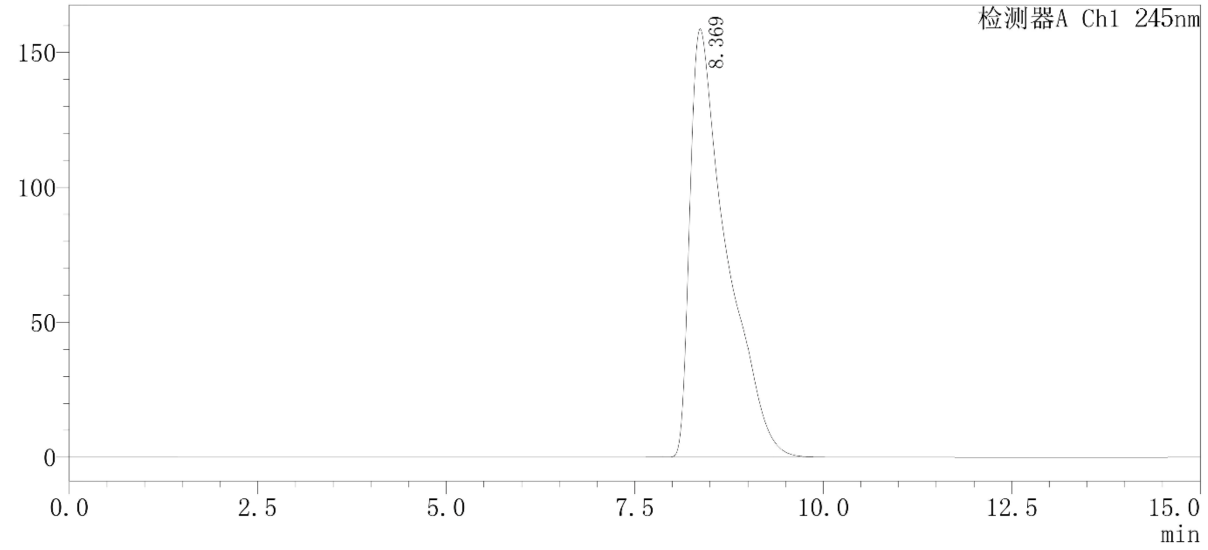

HPLC conditions: Shiseido Capcell PAK C18 (150×4.0 mm, 5 μm) was used as the column at 30 ˚C, and the mobile phase flow rate was 1 mL/min. During the analytical run, the elution was carried out using mobile phases A (Ultrapure water) and B (acetonitrile), the percentage of mobile phases B was 30%, while the detection wavelength was 245 nm.

(6) ^{1}H, ^{13}C-NMR, HRMS and HPLC of **6**.

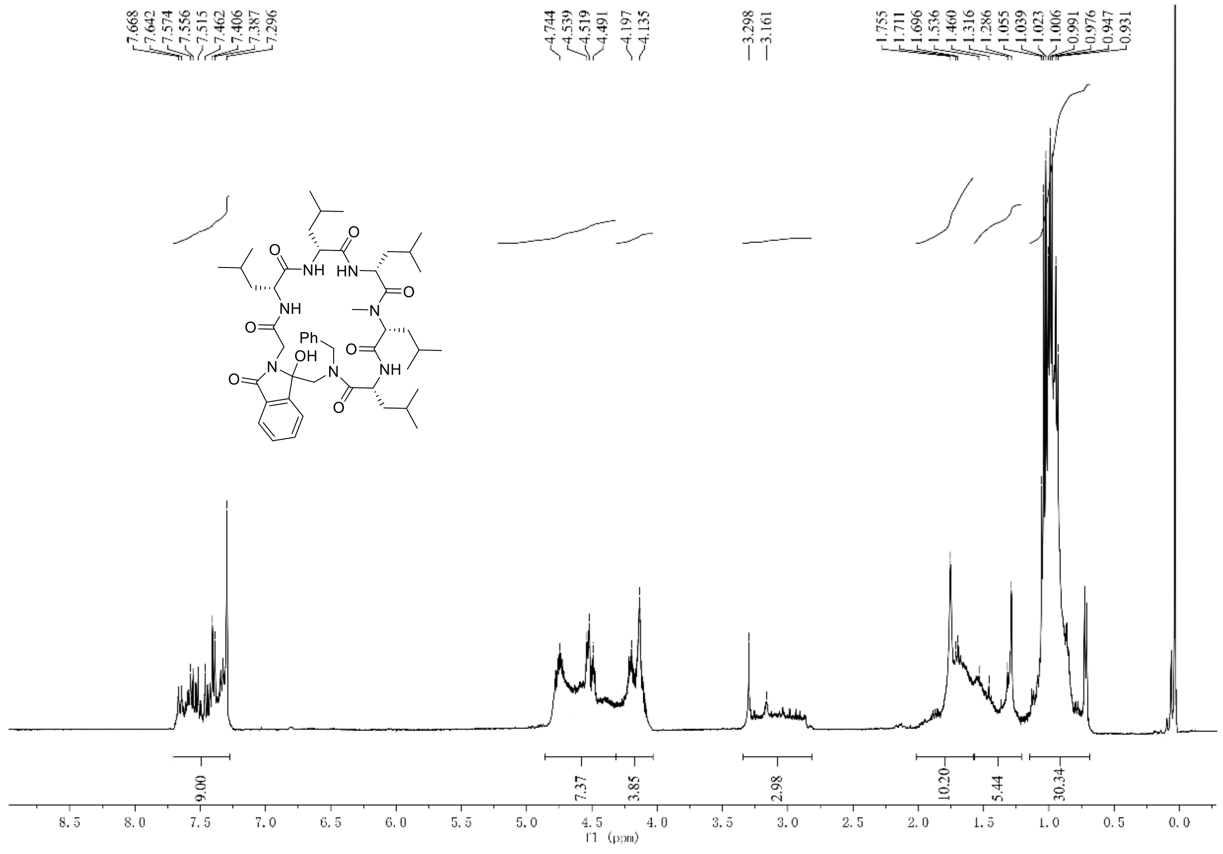

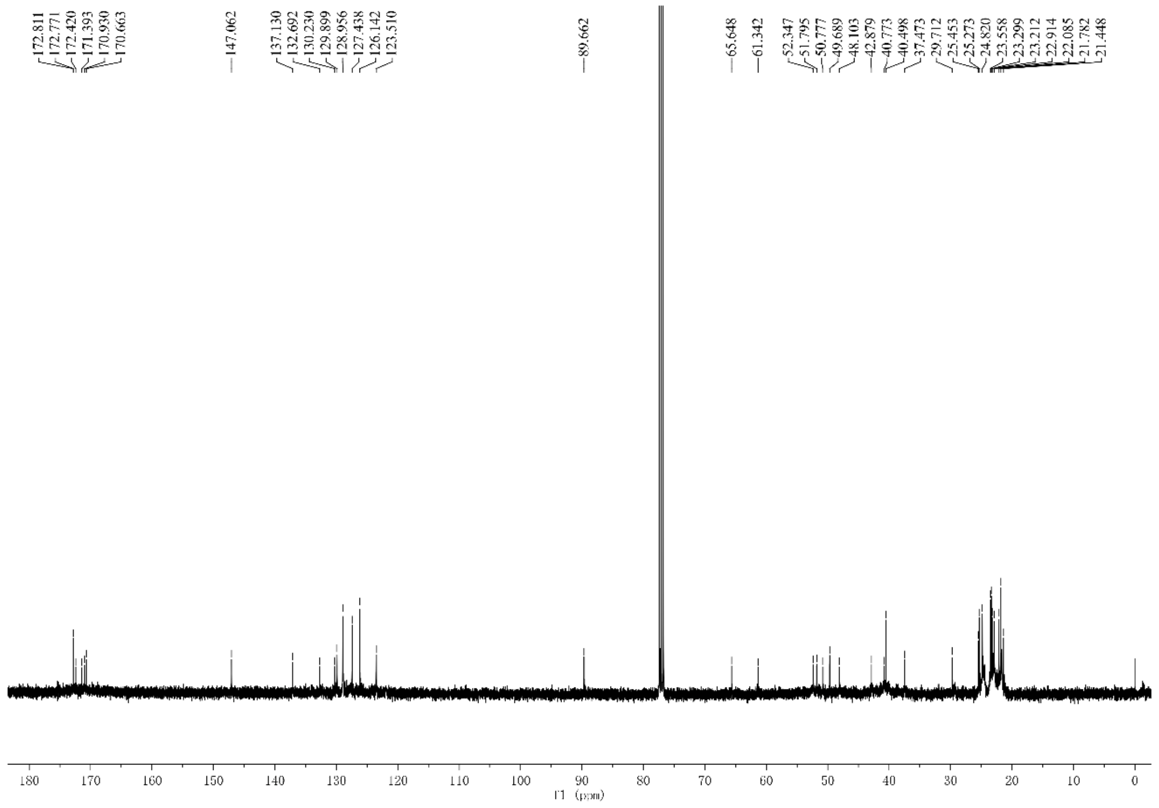

172.811
172.771
172.420
171.393
170.930
170.663
147.062
137.130
132.692
130.230
129.899
128.956
127.438
126.142
123.510
89.662
65.648
61.342
52.347
51.795
50.777
49.689
48.103
42.879
40.773
40.498
37.473
29.712
25.453
25.273
24.820
23.558
23.299
23.212
22.914
22.085
21.782
21.448
f1 (ppm)

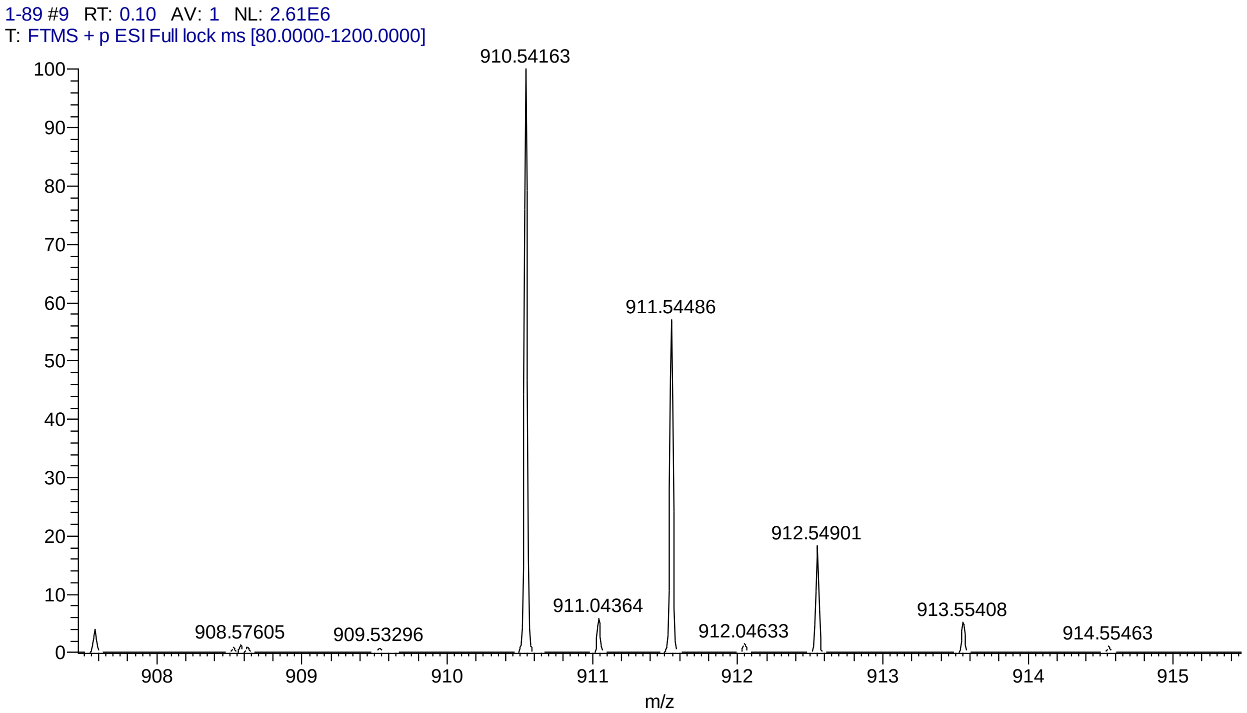

1-89 #9 RT: 0.10 AV: 1 NL: 2.61E6
T: FTMS + p ESI Full lock ms [80.0000-1200.0000]
910.54163
911.54486
912.54901
911.04364
913.55408
908.57605
909.53296
912.04633
914.55463
m/z

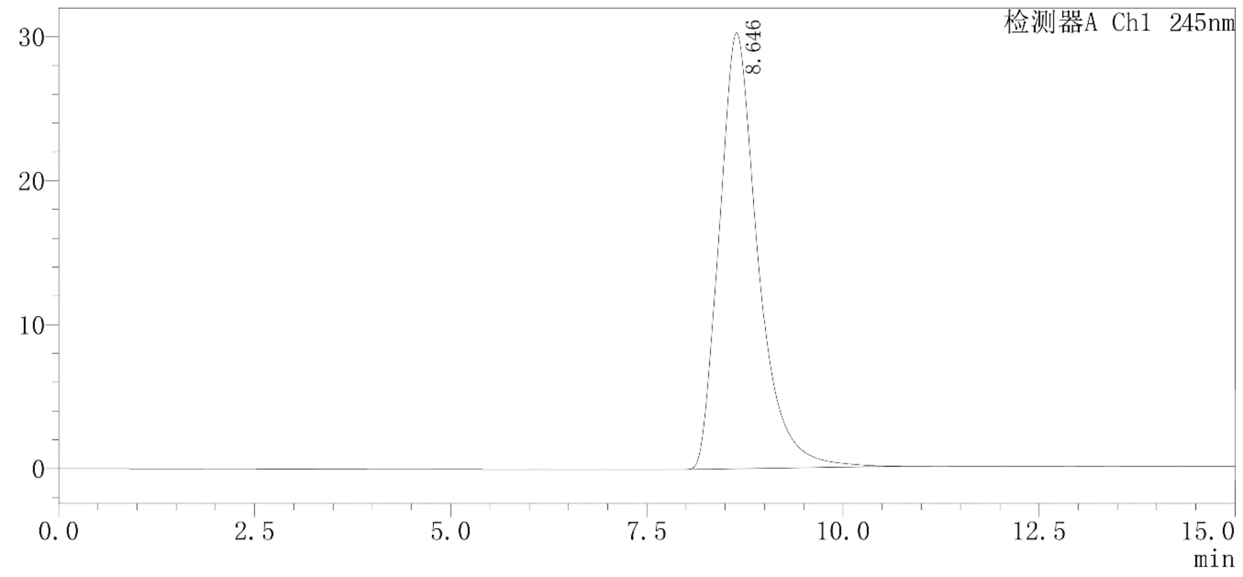

HPLC conditions: Shiseido Capcell PAK C18 (150×4.0 mm, 5 μm) was used as the column at 30 ˚C, and the mobile phase flow rate was 1 mL/min. During the analytical run, the elution was carried out using mobile phases A (Ultrapure water) and B (acetonitrile), the percentage of mobile phases B was 30%, while the detection wavelength was 245 nm.

(7) ^{1}H, ^{13}C-NMR, HRMS and HPLC of **7**.

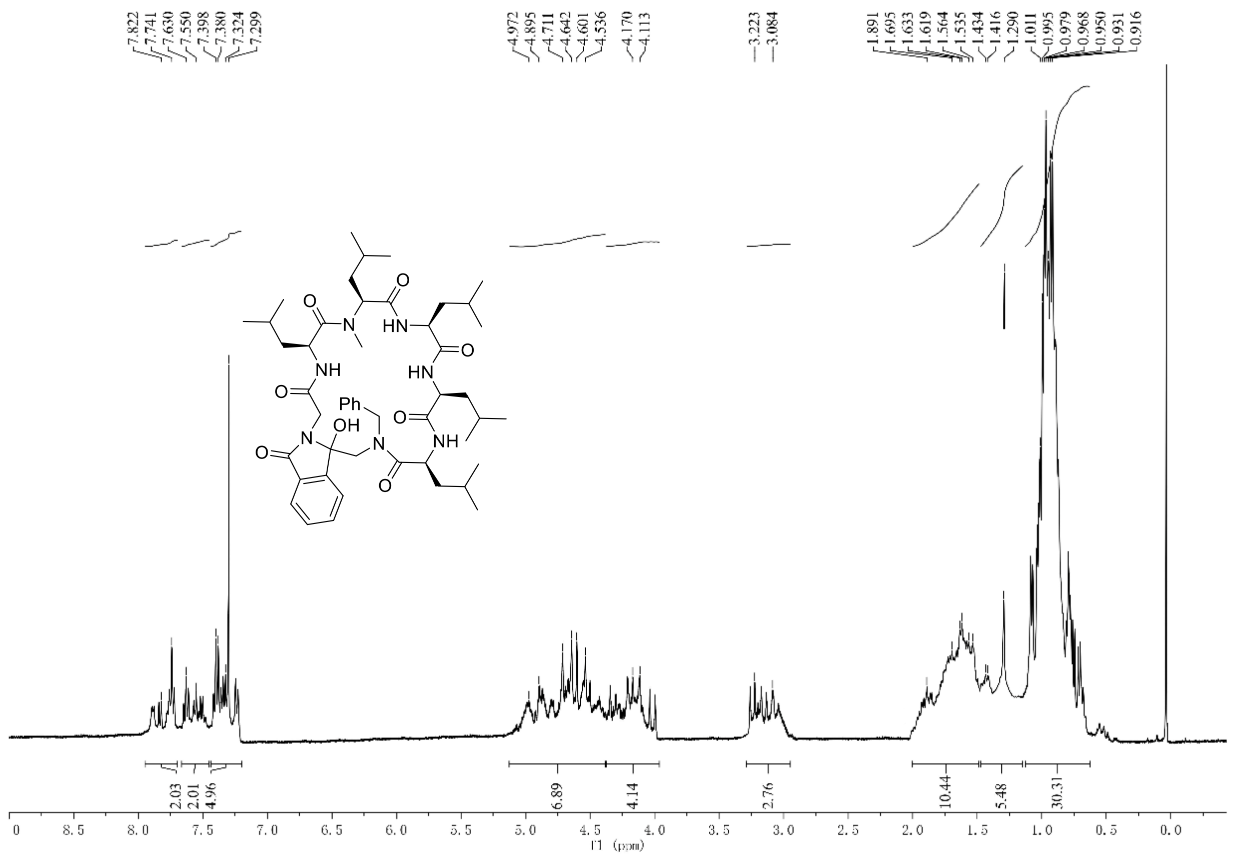

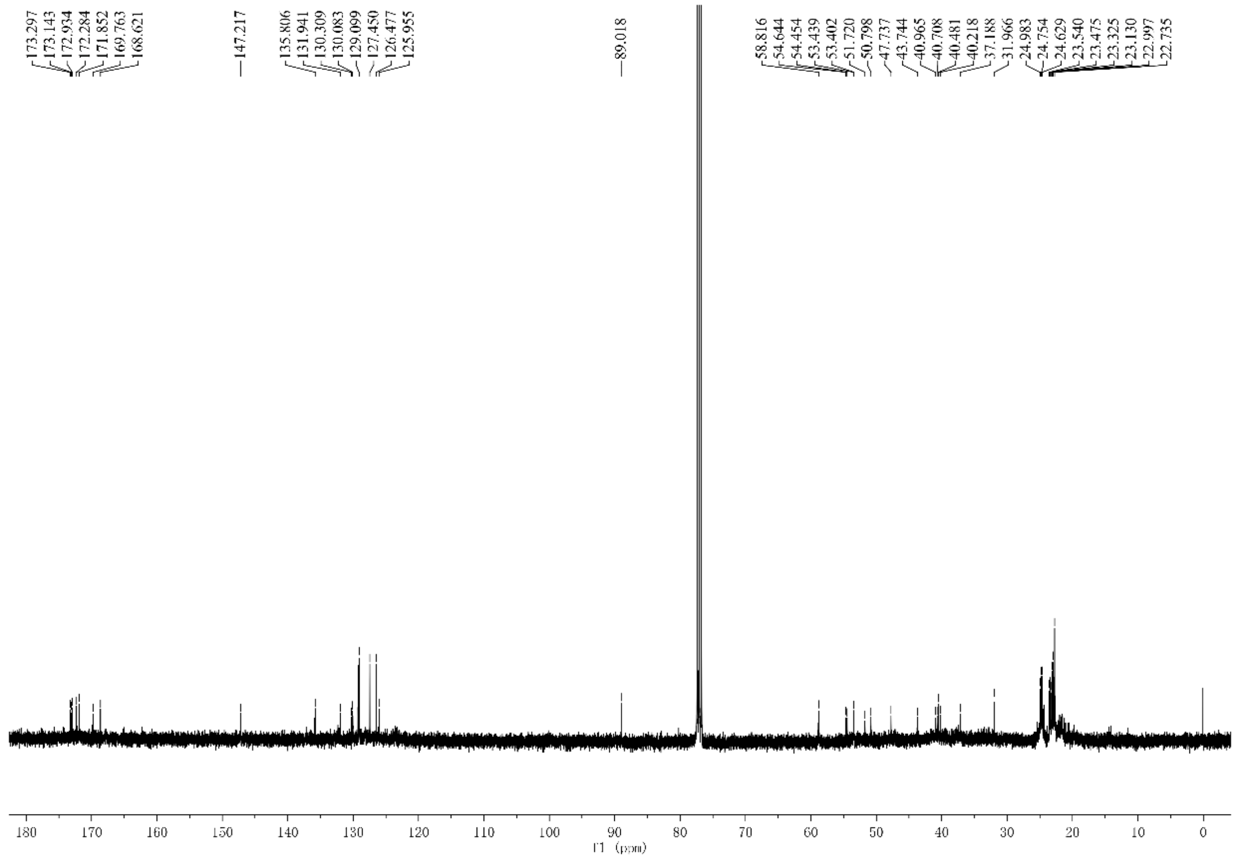
173.297
173.143
172.934
172.284
171.852
169.763
168.621
147.217
135.806
131.941
130.309
130.083
129.099
127.450
126.477
125.955
89.018
58.816
54.644
54.454
53.439
53.402
51.720
50.798
47.737
43.744
40.965
40.708
40.481
40.218
37.188
31.966
24.983
24.754
24.629
23.540
23.475
23.325
23.130
22.997
22.735
f1 (ppm)

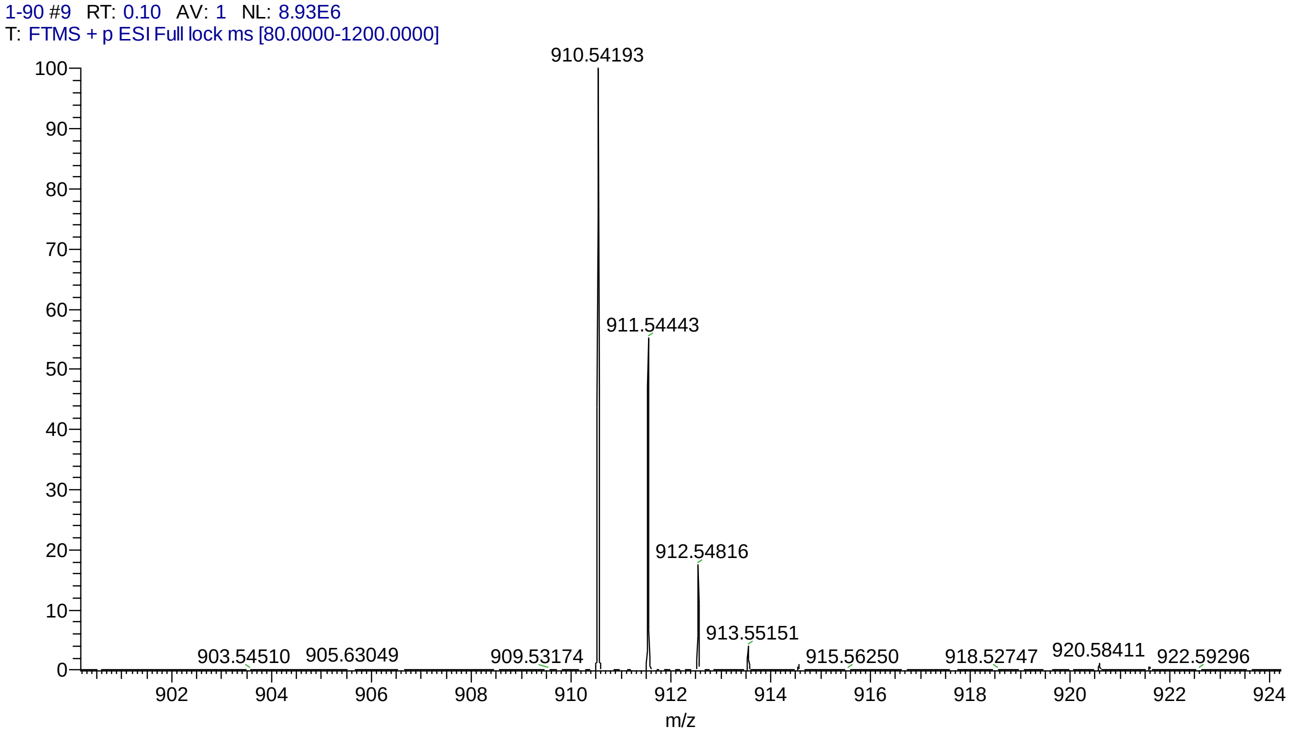
1-90 #9 RT: 0.10 AV: 1 NL: 8.93E6
T: FTMS + p ESI Full lock ms [80.0000-1200.0000]
910.54193
911.54443
912.54816
913.55151
903.54510
905.63049
909.53174
915.56250
918.52747
920.58411
922.59296
m/z

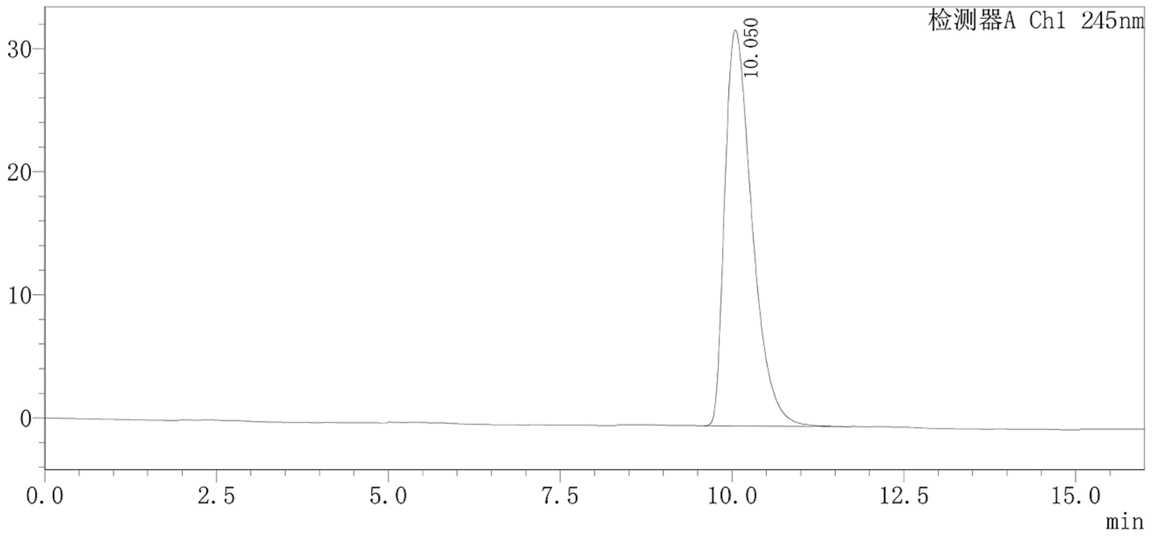

HPLC conditions: Shiseido Capcell PAK C18 (150×4.0 mm, 5 μm) was used as the column at 30 ˚C, and the mobile phase flow rate was 1 mL/min. During the analytical run, the elution was carried out using mobile phases A (Ultrapure water) and B (acetonitrile), the percentage of mobile phases B was 30%, while the detection wavelength was 245 nm.

(8) ^{1}H, ^{13}C-NMR, HRMS and HPLC of **8**.

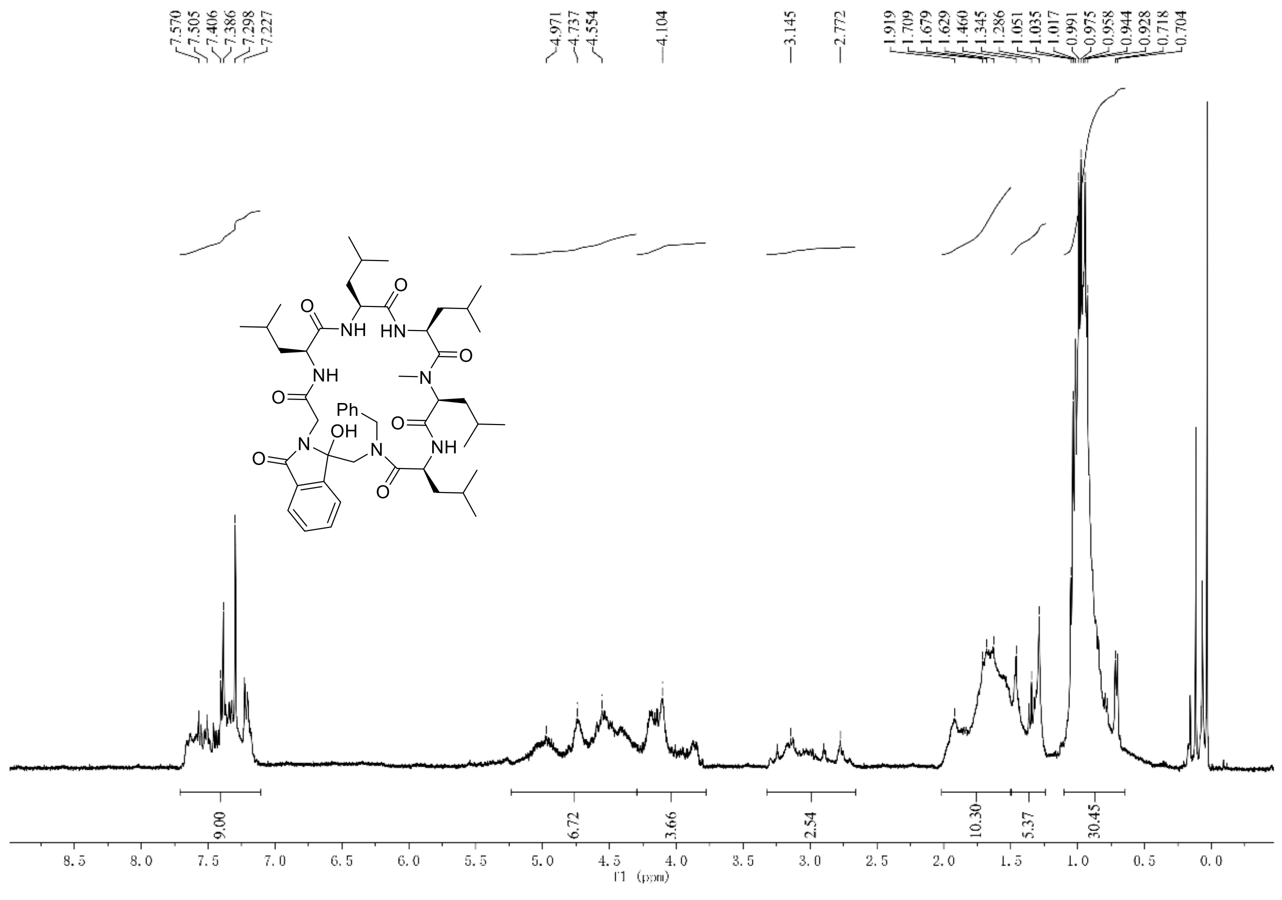

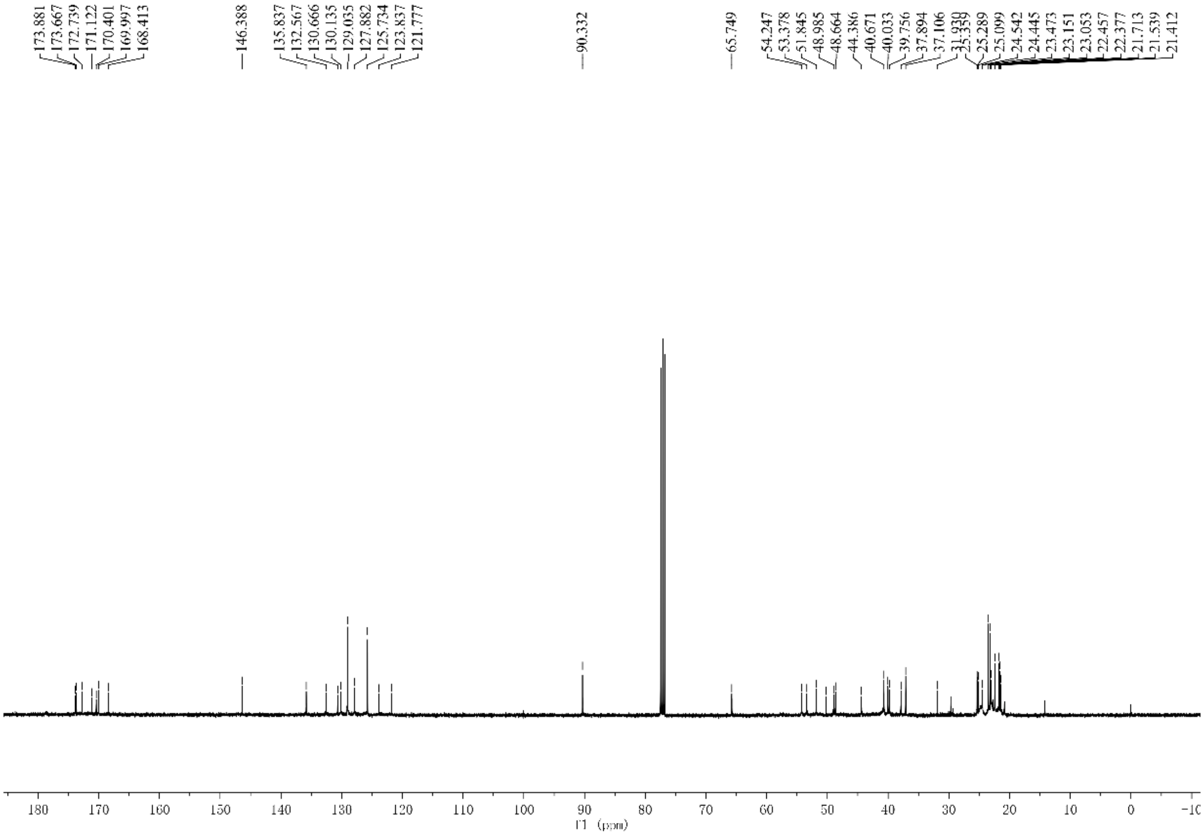
173.881
173.667
172.739
171.122
170.401
169.997
168.413
146.388
135.837
132.567
130.666
130.135
129.035
127.882
125.734
123.837
121.777
90.332
65.749
54.247
53.378
51.845
48.985
48.664
44.386
40.671
40.033
39.756
37.894
37.106
31.939
25.359
25.289
25.099
24.542
24.445
23.473
23.151
23.053
22.457
22.377
21.713
21.539
21.412
180 170 160 150 140 130 120 110 100 90 80 70 60 50 40 30 20 10 0 -10
f1 (ppm)

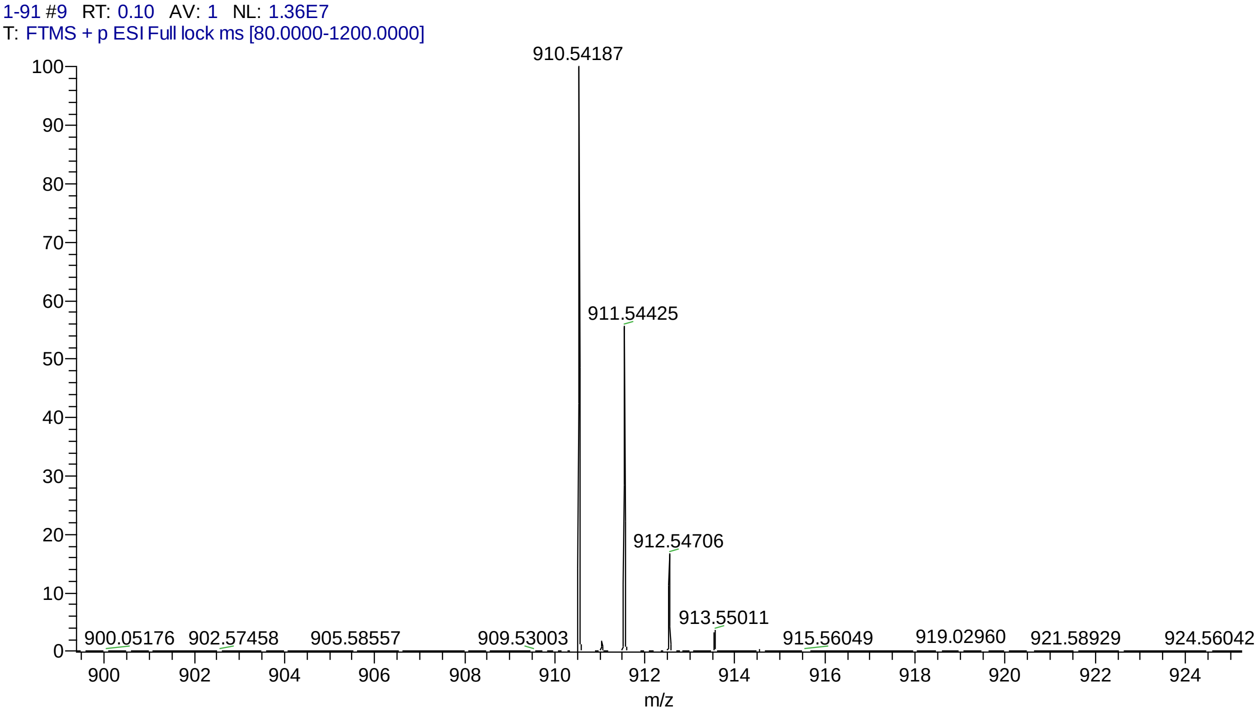
1-91 #9 RT: 0.10 AV: 1 NL: 1.36E7
T: FTMS + p ESI Full lock ms [80.0000-1200.0000]
910.54187
911.54425
912.54706
913.55011
900.05176
902.57458
905.58557
909.53003
915.56049
919.02960
921.58929
924.56042
m/z

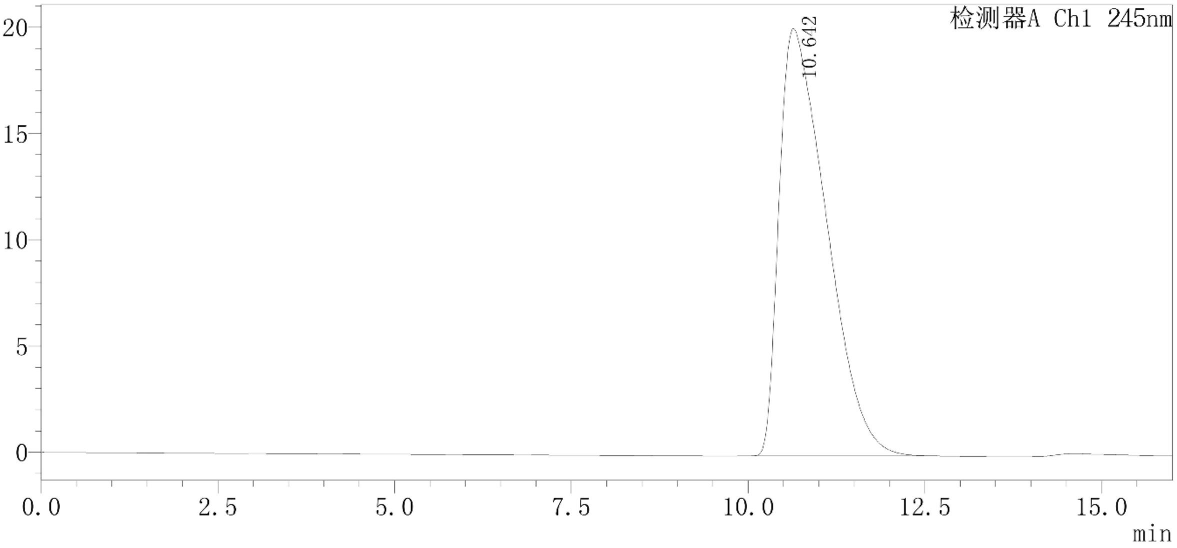

HPLC conditions: Shiseido Capcell PAK C18 (150×4.0 mm, 5 μm) was used as the column at 30 ˚C, and the mobile phase flow rate was 1 mL/min. During the analytical run, the elution was carried out using mobile phases A (Ultrapure water) and B (acetonitrile), the percentage of mobile phases B was 30%, while the detection wavelength was 245 nm.
